# Structure-Aided Computational Design of Triazole-Based Targeted Covalent Inhibitors of Cruzipain

**DOI:** 10.3390/molecules29174224

**Published:** 2024-09-05

**Authors:** Juan Pablo Cerutti, Lucas Abreu Diniz, Viviane Corrêa Santos, Salomé Catalina Vilchez Larrea, Guillermo Daniel Alonso, Rafaela Salgado Ferreira, Wim Dehaen, Mario Alfredo Quevedo

**Affiliations:** 1 Unidad de Investigación y Desarrollo en Tecnología Farmacéutica (UNITEFA-CONICET), Facultad de Ciencias Químicas, Universidad Nacional de Córdoba (FCQ-UNC), Haya de la Torre y Medina Allende, Córdoba 5000, Argentina; jpcerutti@unc.edu.ar; 2 Sustainable Chemistry for Metals and Molecules, Department of Chemistry, KU Leuven, Celestijnenlaan 200F, 3001 Leuven, Belgium; wim.dehaen@kuleuven.be; 3 Departamento de Bioquímica e Imunologia, Instituto de Ciências Biológicas, Universidade Federal de Minas Gerais, Av. Antônio Carlos 6627, Belo Horizonte 31270-901, Brazil; lucasdiniz1478@gmail.com (L.A.D.); vivicorreasantos@gmail.com (V.C.S.); rafaelasalgadoferreira@gmail.com (R.S.F.); 4 Instituto de Investigaciones en Ingeniería Genética y Biología Molecular (INGEBI-CONICET), Vuelta de Obligado 2490, Ciudad de Buenos Aires 1428, Argentina; vilchez.ingebi@gmail.com (S.C.V.L.); galonso@dna.uba.ar (G.D.A.)

**Keywords:** Chagas disease, Cruzipain, triazole derivatives, targeted covalent inhibitors, computer-aided drug design

## Abstract

Cruzipain (CZP), the major cysteine protease present in *T. cruzi*, the ethiological agent of Chagas disease, has attracted particular attention as a therapeutic target for the development of targeted covalent inhibitors (TCI). The vast chemical space associated with the enormous molecular diversity feasible to explore by means of modern synthetic approaches allows the design of CZP inhibitors capable of exhibiting not only an efficient enzyme inhibition but also an adequate translation to anti-*T. cruzi* activity. In this work, a computer-aided design strategy was developed to combinatorially construct and screen large libraries of 1,4-disubstituted 1,2,3-triazole analogues, further identifying a selected set of candidates for advancement towards synthetic and biological activity evaluation stages. In this way, a virtual molecular library comprising more than 75 thousand diverse and synthetically feasible analogues was studied by means of molecular docking and molecular dynamic simulations in the search of potential TCI of CZP, guiding the synthetic efforts towards a subset of 48 candidates. These were synthesized by applying a Cu(I)-catalyzed azide-alkyne cycloaddition (CuAAC) centered synthetic scheme, resulting in moderate to good yields and leading to the identification of 12 hits selectively inhibiting CZP activity with IC_50_ in the low micromolar range. Furthermore, four triazole derivatives showed good anti-*T. cruzi* inhibition when studied at 50 μM; and **Ald-6** excelled for its high antitrypanocidal activity and low cytotoxicity, exhibiting complete in vitro biological activity translation from CZP to *T. cruzi*. Overall, not only **Ald-6** merits further advancement to preclinical in vivo studies, but these findings also shed light on a valuable chemical space where molecular diversity might be explored in the search for efficient triazole-based antichagasic agents.

## 1. Introduction

Chagas disease (CD), also known as American trypanosomiasis, is defined by the WHO as a potentially life-threatening infectious systemic disease caused by the protozoan parasite *Trypanosoma cruzi* (*T. cruzi*) [1,2,3]. Approximately 6.5 million individuals globally suffer from CD, with an annual incidence of 30,000 new cases and up to 13,000 deaths per year [1,2,3,4,5,6]. Despite its significant health burden, CD is one of the thirteen most neglected tropical diseases and the third most common parasitic disease worldwide, just after malaria and schistosomiasis [1,5], highlighting the need for policies capable of addressing the public health challenges faced by the affected regions and countries [2,3].

The development of new drug candidates for the treatment of CD constitutes a very active scientific topic among the academic research community [7]; however, to date, only two nonspecific drugs approved about 50 years ago still constitute the unique treatment options: nifurtimox (1940, NFX) and benznidazole (1974, BZN). Despite the efficacy of NFX and BZN in primarily addressing acute stages of the disease, the severity and high number of adverse effects associated with their clinical use significantly limits their pharmacotherapeutic application, ultimately resulting in poor patient adherence and drug resistance.

**Figure 1:**
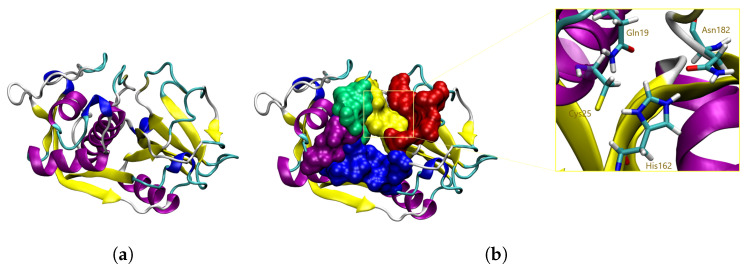
(**a**) 3D structure of CZP (PDB 3IUT [22]). (**b**) 3D structure of CZP active site, highlighting the S1’ (red), S1 (green), S2 (blue), S3 (purple) and catalytic (yellow) subsites. The catalytic triad and oxyanion hole are represented in licorice.

By the end of the 1970s, a group of cysteine proteases (CP) were identified as crucial components of the *T. cruzi* biological machinery; with one representative attracting special interest as a potential druggable target. This CP was named Cruzipain (CZP) by Cazzulo et al. [8], and is nowadays known to represent the main CP involved in the *T. cruzi* lifecycle. Its catalytic domain consists of a single polypeptide chain of 215 amino acid (AA) residues, forming α-helices and antiparallel β-sheets that fold in two distinct subdomains and delineate the active site located within the interface (Figure 1a) [9,10]. It includes a catalytic triad (Cys25, His162 and Asn182) responsible for the enzyme hydrolytic activity [11,12] and four subsites identified as S1’, S1, S2 and S3 [8,9,10] (Figure 1b). Detailed structural and topological exploration has been reported for CZP substrates and inhibitors interacting within these four subsites, with different extents of structure–activity relationships being reported [8,9,10,13,14,15,16,17,18,19,20,21].

A broad diversity of CZP inhibitors has been reported to date, including both non-covalent and covalent inhibitors resulting either from drug repurposing or de novo synthesis [16,17,18,23,24,25,26,27,28,29,30,31,32,33,34,35]. In recent years, increased scientific interest has been observed towards the development of a specific class of covalent inhibitors identified as targeted covalent inhibitors (TCI) [36,37], in which both a maximized complementarity and modulated reactivity with the binding site operate in a synergistic way towards safely and effectively inhibiting the corresponding target. Both of these features can be efficiently optimized by applying modern bioinformatic approaches and technologies related to structure-based drug design (SBDD). Several examples of clinically useful TCI have been reported in the literature [36,37,38,39]; however, to the best of our knowledge, none targeting CZP are currently under clinical development to date, while most of the preclinical candidates lack adequate translation of inhibitory activity from CZP to *T. cruzi* [40].

**Figure 2:**
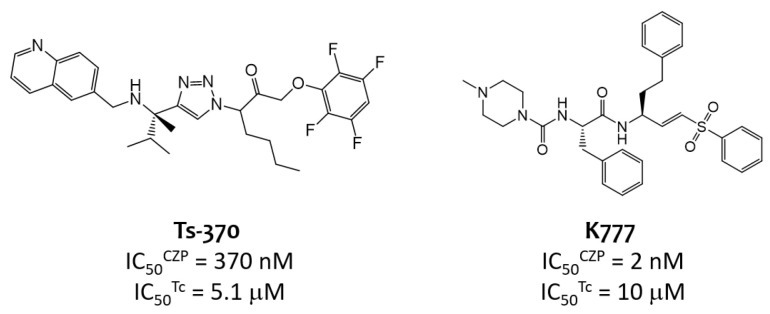
Peptidomimetic (Ts-370) and peptide-based (K777) promising CZP inhibitors reported in literature, with poor translation to *T. cruzi* inhibitory activity [32,45].

Most CZP inhibitors present a peptide-like structure that confers them with unfavorable physicochemical properties and suboptimal biopharmaceutic performance, such as a low oral bioavailability, poor stability and high rate of first pass metabolism [41,42,43]. Particular interest has been placed in strategies involving bioisosteric peptide bond replacement to further enhance these aspects. One example is the report by Brak et al. in 2008 [44], which developed a lead CZP-inhibitor based on the 1,4-disubstituted 1,2,3-triazole scaffold. Although this compound exhibited a high CZP inhibitory potency (Figure 2, **Ts-370**, IC_50_^*CZP*^ = 370 nM), its bioactivity was not efficiently translated into *T. cruzi* inhibition, as reflected by its 14-fold lower antitrypanosomal potency (IC_50_^*Tc*^ = 5.1 μM). This lack of translation constitutes a recognized hurdle for the development of effective trypanocidal agents and has also been reported for the covalent inhibitor **K777** (Figure 2), which stands out for its high CZP inhibitory potency (IC_50_^*CZP*^ = 2 nM) but translates into IC_50_^*Tc*^ values of ≈10 μM. Montanari et al. recently reported a carefully designed machine-learning-based study aimed to assess the chemical space shared by candidates combining high CZP and *T. cruzi* inhibitory activity [40]. Interestingly, the authors concluded that only a small chemical space associated with CZP inhibition intersects with high *T. cruzi* bioactivity. This limited shared bioactive chemical space is further narrowed if selectivity requirements for CZP inhibition relative to homologous CP, such as human L-cathepsin (hCatL) [10], are taken into consideration. In this context, carefully designed protocols capable of combinatorially screening CZP inhibitors covering a wide chemical space are required for the advancement in the preclinical identification of TCI meriting further progress as antichagasic agents.

In this work, a SBDD approach was applied to combinatorially generate molecular diverse libraries of 1,4-disubstituted 1,2,3-triazole derivatives, further explored by means of molecular modeling (i.e., molecular docking and molecular dynamics) as potential TCI of CZP with maximized complementarity with the corresponding CZP active site. In this search, the atomistic details and known structure–activity relationships with respect to CZP S1’, S1, S2 and S3 subsites were also comprehensively explored.

Overall, a virtual library containing 78,540 diverse drug-like 1,4-disubstituted 1,2,3-triazole analogues was exhaustively studied in silico, further identifying 22 promising candidates exhibiting a binding mode compatible with high CZP inhibitory activity. By applying a versatile Cu(I)-catalyzed azide-alkyne cicloaddition (CuAAC) centered synthetic scheme, the 22 positive binder candidates were obtained and further complemented with a set of 23 moderate to poor performing binders included as inhibitor decoys. The bioactivity of this set of triazole-derivatives was explored in detail, including in vitro measurements of inhibitory activity against CZP and *T. cruzi* in infection cell assays, as well as their selectivity over hCatL and cytotoxicity over human cells.

## 2. Results and Discussion

### 2.1. Virtual High Throughput Screening

#### 2.1.1. Development of an SBDD Screening Workflow

In order to validate the virtual high throughput screening (vHTS) conditions for hit identification, a set of 20 triazole-based CZP inhibitors bearing a¸ tetrafluorophenoxymethyl ketone (4FPMK) warhead (WH) was used as a training set [46] (Figure S1 and Table S1). The reported crystallographic structure of **Ts-370** (PDB 3IUT [22]) was considered as reference for the identification of the corresponding bioactive binding poses (see Experimental section for technical details).

Among the diverse docking approaches available, in this study, a classical non-covalent docking protocol was applied, with no bias (i.e., covalent docking or grid based) being included with respect to the orientation of the corresponding WH. Thus, it was possible to evaluate the overall binding pose clusters resulting from the orientation of **R**^1^, **R**^2^ and **R**^3^ within each subsite (S1, S2, S3), further analyzing the resulting reactive pose in the context of the landscape of alternative non-reactive orientations.

The molecular docking search parameters were systematically explored until bioactive poses were successfully identified not only for **Ts-370** (Figure S2, RMSD value < 1 Å respect to the crystal) but also for the remaining 19 analogues. The final pose selection criteria to identify hits included a combination of the resulting docking score, cluster size and satisfaction of four H-bond contacts within the CZP active site (i.e., Gln19, Ser61, Gly65, Gly66). As shown in Figure S3a, a clear trend of more negative docking score values was found for the reference compounds exhibiting lower IC_50_, confirming the predictive power of the molecular docking protocol to identify bioactive poses and sort them according to their inhibitory potency.

To describe the time-dependent conformational evolution of the identified docked poses for the training set, they were subjected to explicit solvent classical molecular dynamics (MM-MD) simulations at 300 K, with free-energy MMPBSA analyses performed on the resulting simulated trajectories. All encounter complexes showed appropriate stability during the simulated time (100 ns); and, as can be seen in Figure S3b, the results obtained also allowed an accurate correlation between the ligand potencies and their predicted Δ$G_{bind}$. Additionally, the electrostatic (EEL) and van der Waals (vdW) interaction patterns analyzed for each ligand–CZP complex (Figures S4 and S5) were in agreement with the reported structure–activity relationships (SAR) of S1’, S2 and S3 subsites.

Correspondingly, the molecular-docking-centered search protocol showed adequate efficiency to screen triazole-based CZP inhibitors, effectively discriminating the most potent candidates.

#### 2.1.2. Construction of the Working Chemical Space

**Figure 3:**
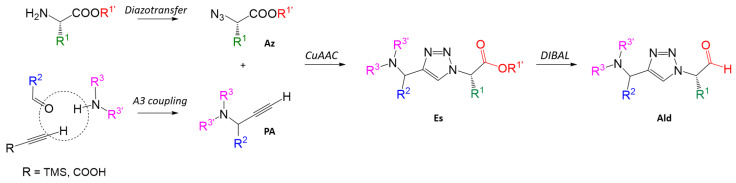
Synthetic core scheme for the synthesis of 1,4-disubstituted 1,2,3-triazole-based potential inhibitors of CZP.

The chemical space associated with the molecular diversity to be explored was based on a synthetic scheme designed to obtain 1,4-disubstituted 1,2,3-triazoles (Figure 3), which allows the combinatorial exploration of **R**^1^, **R**^2^ and **R**^3^ substitutions. In this respect, a Cu(I)-catalyzed 1,3-dipolar cycloaddition (CuAAC [47,48,49,50,51]) was considered as the core of the scheme, allowing the combinatorial coupling of libraries of azides and alkynes.

For the synthesis of azides (**Az**), an ISA.H_2_SO_4_-mediated diazotransfer reaction [52,53] to transform α-amino esters into their corresponding azido-analogues was considered; while for the alkyne building blocks, the approach was based on MW-assisted A3 coupling reactions [54,55,56], resulting in a set of terminal propargylamines (**PA**). Additionally, two alternative WH groups were explored: esters (**Es**) and aldehydes (**Ald**), with the former being obtained from α-azido esters via CuAAC, while the latter were derived from the resulting **Es** by applying a DIBAL-based reduction [57]. Further details regarding the corresponding synthetic procedures are provided in Section 2.2 (Synthesis of selected candidates and biological evaluation against CZP), Section 3.2 (Synthetic procedures) and Supplementary Materials.

Once the synthetic protocols were defined, and considering the great diversity of potential starting materials that could be employed, massive virtual screening libraries were constructed by enumerating the presented chemical reactions and evaluated using the vHTS method developed for the identification of promising candidates. These libraries were built and managed using TidyScreen (https://github.com/alfredoq/TidyScreen, accessed on 1 March 2024), an open-source Python-based in-house developed framework that leverages a structured SQL database to streamline the organization, combinatorial synthesis and molecular docking execution of large screening projects (see Section 3.1.1 for further details). Thus, the database of organic molecules eMolecules [58] was used as a building block source for the combinatorial construction of massive virtual libraries of triazole derivatives. In order to narrow down the size of the libraries to drug-like candidates, those exhibiting favorable properties (i.e., MW < 700 Da, *ClogP* between 0 and 6, less than 5 HBD and 10 HBA groups, chiral centers < 3, and a max. of 10 rotatable bonds) were considered for further exploration.

Overall, a set of 1517 AA, 6022 aldehydes and 4037 amines were retained for in silico synthesis. Considering the large number of building block combinations (>300 billion possibilities), a strategy applying an initial stepwise comprehensive regional virtual screening to identify the most promising **R**^1^, **R**^2^ and **R**^3^ substituents preceded a final combinatorial analysis of the passing results, as described in detail in the following sections.

#### 2.1.3. Virtual Screening on $R^{1}$

**Figure 4:**
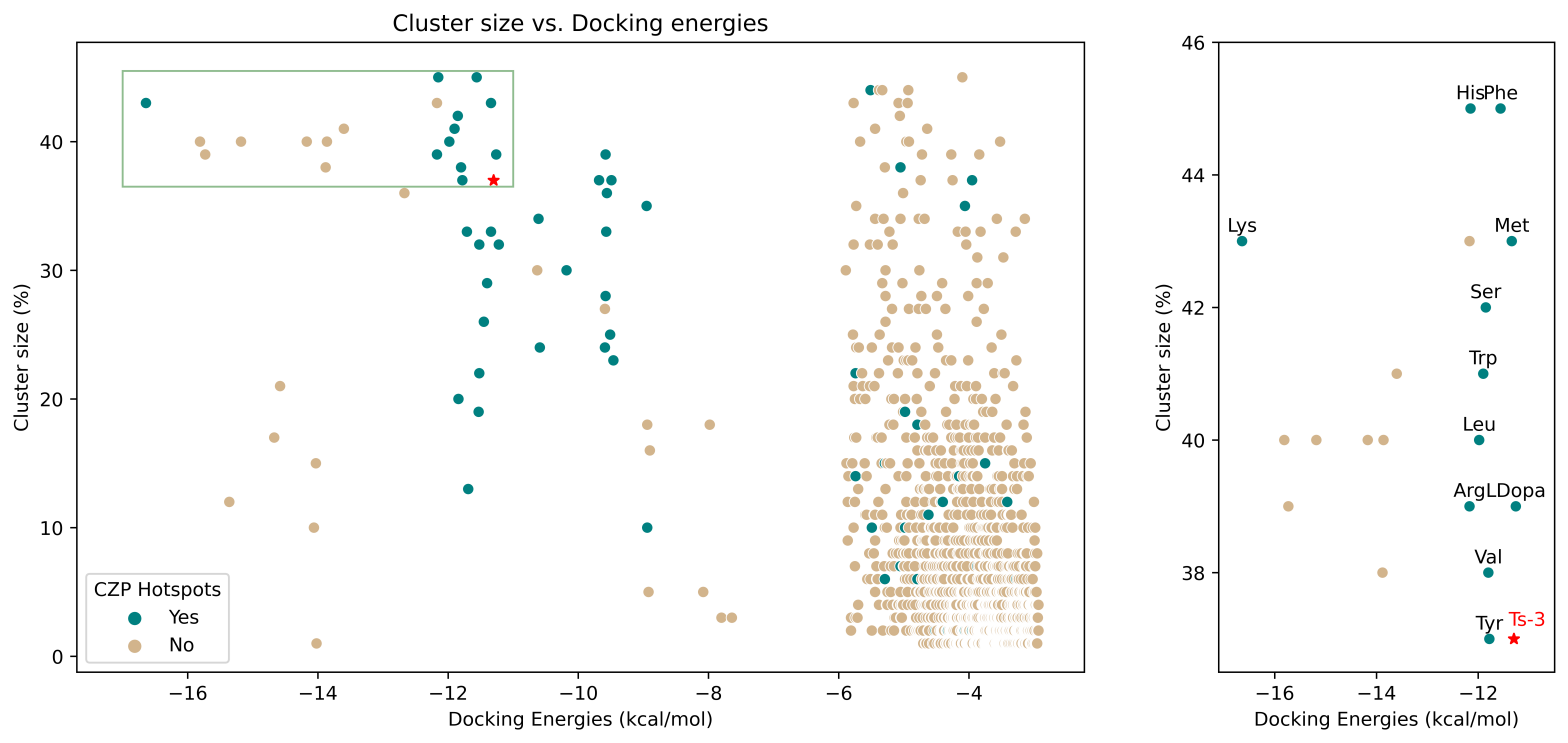
Summary of the docking-based vHTS results focused on **R**^1^ with **CZP**.

The chemical space on **R**^1^ was screened in silico evaluating a set of 1517 AA, while keeping constant the cyclopentyl, pyrazolopyrimidine and 4FPMK scaffolds of **Ts-3** as **R**^2^, **R**^3^ and WH, respectively. The set of derivatives generated were subjected to molecular docking studies, with results being classified based on the hit identification criteria defined as part of the protocol validation stage (i.e., lowest energy and most populated cluster, complying with the pharmacophoric intermolecular interactions). This selection procedure is graphically presented in (Figure 4), with the upper left region corresponding to derivatives identified as preferred **R**^1^ substituents. From the 35 promising **R**^1^ (Figure S6), it is worth highlighting the identification of Arg, Lys, His and Phe as promising starting materials, exhibiting even lower docked energies compared to the reference compound **Ts-3** (Figure 4), consistent with the known preference of CZP for basic and/or aromatic AA side chains oriented towards S1. In addition, a wide range of physicochemical properties is observed, with scaffolds ranging from totally apolar to ionizable (cationic); highlighting the chemical space versatility that can be explored in the search for adequate biological activity translation.

As was found for the training set during the vHTS protocol validation, most of the ligands oriented their **R**^1^ substituent towards the bulk of the solvent. A notable exception was the ligand derived from Lys (**R**^1^ **Lys-4FPMK**, Figure S7a), which exhibited more favorable docking energies compared to the rest of the candidates (−16.64 kcal·mol^−1^ vs. −12.17 kcal·mol^−1^ for the second with the lowest energy, Figure 4), placing it as the top-ranked derivative resulting from the docking campaign. Visual inspection of its binding mode evidenced an extra contact point not typically identified for compounds in the training set. Specifically, the methylcarboxylate side chain of Asp161, located at the S1’/S2 interface of the CZP binding site, is able to interact with the ionized amine moiety of the Lys side chain strongly favored by the flexibility, size and length of the *n*-butylamino group (Figure S7b).

#### 2.1.4. Virtual Screening on R^2^

The molecular diversity generated on the **R**^2^ substitution resulted from 6022 selected aldehydes, which were screened by maintaining the **R**^1^, **R**^3^ and WH moieties of **Ts-3**. Upon construction of the library and applying the docking procedures, 51 promising substructures were identified (Figures S8 and S9). Among the privileged **R**^2^ moieties, two main groups can be distinguished: aromatic and aliphatic substituents, with a reduced amount of heteroatomic scaffolds, in accordance with the highly recognized hydrophobicity of S2 [8,13,14,15,16,17,18,19]. It is worth noting the outstanding result achieved with the derivative bearing a cyclohexyl group, not only in terms of energy but also in the population of the docked cluster (*cHex*, Figure S9).

#### 2.1.5. Virtual Screening on $R^{3}$

Screening on **R**^3^ was accomplished by considering 4037 amine building blocks as alternatives of the pyrazolopyrimidin-5-ylmethanamine moiety present in **Ts-3**, resulting in a total of 22 scaffolds being identified as privileged based on their complementarity within the S3 subsite (Figures S10 and S11). Most of them exhibit at least one H-bond interaction with the hydroxymethyl side chain of Ser61, while no aromatic amine passed the bioactive pose filtering criteria. These observations highlight the role of Ser61 in ligand binding, emphasizing the need for some degree of flexibility to optimize this interaction. The findings are consistent with the already reported preference of S3 for bulky and/or positively charged aromatic groups [8,10,17,20], which confers binding selectivity for CZP over human-cathepsins [17,18]. It is worth pointing out the remarkable molecular docking performance of the derivative bearing a benzodioxole group as **R**^3^ (BDX, Figure S11), complying with the requirements of bulkiness and H-bond interaction with Ser61, which, to date, has not been reported in CZP inhibitors.

#### 2.1.6. Combinatorial Screening of Favored $R^{1}$, $R^{2}$ and $R^{3}$ with Inclusion of Selected WH

**Figure 5:**
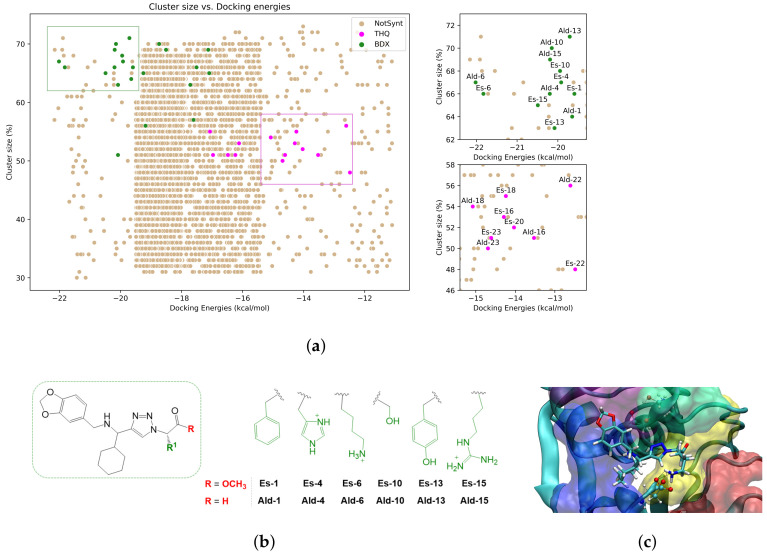
(**a**) Summary of the docking-based vHTS results for the 1,2,3-triazoles obtained by combinatorial synthesis. Clusters of optimal (green box, upper right) and less favored (fuchsia box, bottom right) synthesized compounds are highlighted. (**b**) Chemical structure of the most promising triazole-derivatives identified by means of vHTS and synthesized. (**c**) Docking pose of **Ald-6** (licorice), highlighting its interaction with Asp161 (CPK).

In a last step, a virtual combinatorial library resulting from 35 AA (**R**^1^), 51 aldehydes (**R**^2^), 22 amines (**R**^3^) and 2 reference WH (aldehyde, **Ald** and methylester, **Es**) was constructed to systematically explore the best overall combination of substituents, leading to a library of 78,540 candidates. Upon applying the docking analyses, as expected, most of them passed the interaction-based post-docking hit-selection criteria (*n* = 77,024) with medium to high cluster sizes (31–70%, Figure 5).

Remarkably, about 65% of the new candidates generated from the combinatorial synthesis of promising fragments showed more favorable docking energies (−25 to −17 kcal·mol^−1^) than both the training set (Section 2.1.1, −6.9 to −11.3 kcal·mol^−1^) and those compounds obtained during the regional search on **R**^1^, **R**^2^ and **R**^3^ (Section 2.1.3, Section 2.1.4 and Section 2.1.5; −17 to −5 kcal·mol^−1^). A certain preference could be identified for derivatives bearing cationic, polar and aromatic **R**^1^ groups (Lys, Arg, Ser, Phe, Tyr and His), combined with cHex as **R**^2^ and BDX as **R**^3^ (Figure 5a, *green box*, *top left*). The identification of Lys derivatives (i.e., **Ald-6** and **Es-6**) as promising candidates, outstanding over their analogues mainly with respect to their docking energies, should once again be underlined. Additionally, no significant differences in the resulting docked energies were observed between compounds bearing an Ald or Es as WH; suggesting that any difference in inhibitory activity may be governed by the reactivity of the corresponding WH.

Hence, considering all the commercially available starting materials included as part of the screening campaign, a chemical workspace (*n* = 77,024, Figure S12) was identified in silico to obtain triazole derivatives with good drug-likeness and the potential to act as antichagasic agents; leading to synthetic efforts as disclosed in the following section.

### 2.2. Synthesis of Selected Candidates and Biological Evaluation against CZP

**Figure 6:**
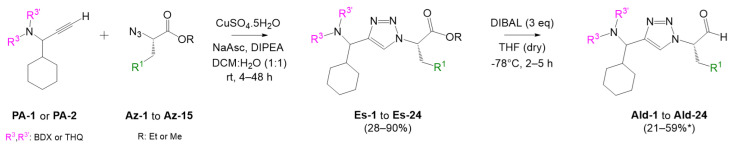
Synthetic core scheme for the synthesis of Es- and Ald-derived 1,4-disubstituted 1,2,3-triazoles. * The yields of Ald-15, Ald-16 and Ald-24 are not included in the range since they could not be isolated.

The synthetic scheme shown in Figure 6 was followed to obtain the corresponding triazole derivatives.

Based on the results obtained from the in silico screening, the main focus was placed on varying the **R**^1^ substituents, using AA classified as optimal (*n* = 6; Arg, Lys, His, Ser, Tyr, Phe), regular (*n* = 5; Cys, Met, Trp, Leu and Val) and suboptimal (*n* = 4; Ala, Thr, Glu, Phg). The first two groups successfully passed the massive virtual screening stages, but with significantly different docking energy and cluster size values, while the AA classified as suboptimal did not overcome any stage of the vHTS. In addition, the c-Hex scaffold was kept constant, since it was identified as a highly privileged **R**^2^ fragment, while two alternative moieties were used to decorate **R**^3^: BDX (capable of establishing H-bond interactions with Ser61) and THQ (with no HBA capability).

As stated in the previous section (Figure 3), the 15 azide building blocks (**Az-1** to **Az-15**) were obtained via an ISA.H_2_SO_4_-mediated diazotransfer reaction adapting the previously reported conditions [52,53], with yields ranging from 16 to 95% (see Section 3.2.2 for further details). **PA-1** was obtained by a copper(I)-catalyzed microwave-assisted A3-coupling reaction [54] followed by KHO-mediated desilylation in methanol. In contrast, the copper(I)-catalyzed decarboxylative A3 coupling reaction reported by Ermolat’ev et al. [55] was employed to obtain **PA-2**, allowing the terminal propargylamine to be yielded in one single step. Details of the reaction conditions and corresponding characterization of the building blocks are described in Section 3.2 Synthetic procedures and Supplementary Materials (pp. 24–28).

Overall, a set of 45 chemically diverse 1,4-disubstituted 1,2,3-triazoles were synthesized via CuAAC (Figure S12) and further subjected to CZP bioassays, as presented in Table 1.

As can be seen, good to excellent synthetic yields (28–90%) were achieved for all combinations of **R**^1^ moieties with c-Hex and BDX or THQ (i.e., **PA-1** and **PA-2**), including an Es WH, resulting in 24 triazole derivatives (**Es-1** to **Es-24**). The bioactivity screening against CZP was performed at 100 μM using E-64 as positive control, a recognized specific covalent irreversible inhibitor of cystein proteases [59]. As expected, E-64 inhibited CZP in 98% (±2%) at 1 μM after 10 min of preincubation, thus validating the experimental setup.

**Figure 7:**
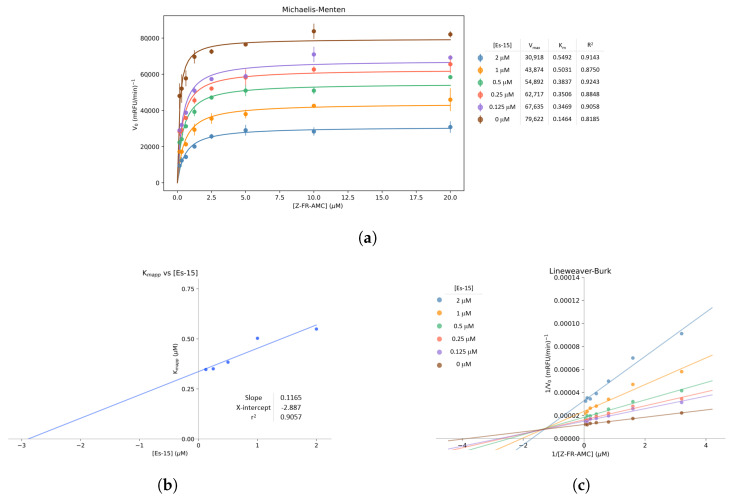
Mechanism of CZP inhibition by **Es-15**, evaluated in triplicate. (**a**) Michaelis–Menten plot. The curves correspond to the fitting of a mixed-model of inhibition to data. (**b**) Plot of the $K_{mapp}$ for varying **Es-15** concentrations (**c**) Lineweaver–Burk reciprocal plot.

Among the 24 Es derivatives evaluated, **Es-10**, **Es-14** and **Es-15** inhibited the enzyme in at least 47%; with **Es-15** excelling for its IC_50_^*CZP*^ of 6.8 ± 0.3 μM. The target-specific mechanism of **Es-15** was confirmed upon observing unchanged CZP inhibition percentages at various concentrations of Triton X-100 and after preincubation with BSA, thus discarding colloidal aggregation [60,61,62] (Table S2). Moreover, a time-dependent inhibitory mechanism was also ruled out for **Es-15** after observing unaltered percentages of CZP activity inhibition upon assays without pre-incubation (81 ± 2%, Table S3). Thus, to determine the inhibitory mechanism of **Es-15**, enzyme initial velocities at different substrate and compound concentrations were monitored. From the Michaelis–Menten graph (Figure 7a), the $V_{max}$, $K_{Mapp}$ and α values were determined. An α value equal to 8.3 indicated a competitive mechanism of inhibition; while a positive slope was determined upon evaluating the $K_{Mapp}$ at varying compound concentrations (Figure 7b). The Lineweaver–Burk plot reveals that $V_{max}$ decreases when **Es-15** concentration increases, as an increment in the intercept value on the ordinate can be observed (Figure 7c). Therefore, the increased $K_{Mapp}$ and decreased $V_{max}$ values upon higher **Es-15** concentration suggest a mixed mechanism of inhibition, with a calculated $K_{i}$ of 3 ± 1 μM (general linear F-test, *p*-value = 0.0127).

The aforementioned observations suggest that the ester group is not acting as an electrophilic center capable of being nucleophilically attacked by Cys25, which might be the main reason for the lack of inhibitory potency observed for the Es derivatives, including **R**^1^, identified as highly favored by in silico screening (Figure 5).

The corresponding **Ald-1** to **Ald-24** derivatives were synthesized from their ester-based precursors with fair to good yields (Table 1). It was not possible to synthesize Ald analogues of **Es-14**, **Es-15** and **Es-24** by applying the DIBAL-mediated reduction, since the crude reaction mixtures were found to be riddled with by-products. Among the **13** compounds bearing BDX as **R**^3^ (**Ald-1** to **Ald-13**), those bearing **R**^1^ scaffolds identified as *optimal* (**Ald-1**, **Ald-4**, **Ald-6**, **Ald-10**, **Ald-13**) inhibited CZP in a range of 64 to 87% at 100 μM; while those derived from moieties predicted as *regular* (**Ald-2**, **Ald-5**, **Ald-7**, **Ald-12**) or *suboptimal* (**Ald-3**, **Ald-8**, **Ald-9**, **Ald-11**) **R**^1^ inhibited the enzyme activity to a lower extent (8 to 64%). Among the best performers, **Ald-6** (**R**^1^: Lys) and **Ald-10** (**R**^1^: Ser) stood out as the most potent ones, with IC_50_ of 3.3 ± 0.3 μM and 3.4 ± 0.5 μM, respectively. Correspondingly, most Ald-based triazoles bearing THQ as **R**^3^ (**Ald-16** to **Ald-23**) exhibited inhibition values ranging from 8% to 55% at 100 μM and were considered inactive, with the exception of **Ald-20** (**R**^1^: Ser), which presented 83% of CZP inhibition and an IC_50_ of 7 ± 4 μM. From the results stated, the marked difference in inhibitory activity of BDX derivatives with respect to THQ-derived ones reinforces the relevance of Ser61 as a hotspot at S3, favoring appropriate CZP recognition.

**Figure 8:**
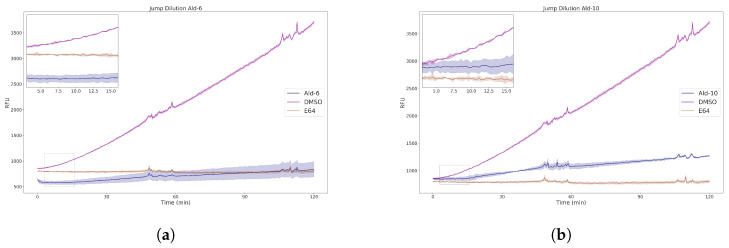
Jump dilution assay results for (**a**) **Ald-6** and (**b**) **Ald-10**. The inhibitors were incubated at 10 × IC_50_ with 100 × [CZP] for 30 min, followed by 100× dilution and 2 h monitoring. The ligand behavior during the first 15 min of the assay is depicted in the magnifications on the top left. The mean and SEM for three replicates are shown.

After ruling out nonspecific enzyme inhibition caused by colloidal aggregation (Table S2), a time-dependent mechanism was verified for **Ald-6** and **Ald-10** based on the significant reduction in their inhibitory percentages without prior incubation with CZP (Table S3). Their modes of action were further examined through jump dilution assays (JDA, Figure 8a,b) [63,64,65,66].

From these studies, it was notable that **Ald-6** and **Ald-10** progress curves exhibited slopes equal to about 12% and 24% of that of the control (DMSO), respectively, showing a homologous behavior to that observed for the widely known covalent irreversible inhibitor E-64, which selectively targets Cys25 [63]. Altogether, the presented mechanistic studies for **Ald-6** and **Ald-10** strongly support the involvement of a covalent irreversible inhibition of the catalytic residue of CZP, which is in agreement with reports on *tight-binders* bearing an aldehyde WH [67,68].

### 2.3. Structure–Activity Relationships (SAR)

From the analysis of the biological and computational results for the set of triazole derivatives evaluated, some conclusions regarding their SAR are worth noting.

The first very important conclusion refers to the efficacy of the bioisosteric replacement of the amide bond by the 1,4-disubstituted 1,2,3-triazole group. In particular, the triazole ring effectively mimics the classical peptide bond of the CZP substrates, complying with stable H-bond interactions with Gly65 and Gly66, as observed in MD simulations (Figure S13); which are confirmed as pharmacophoric contacts.

Although the involvement of the oxyanion hole and the catalytic triad for the mechanistic processes of CZP is well known, their relevance for the formation of the encounter complexes becomes evident here. Gln19 also appears as a pharmacophoric contact, acting as both an H-bond acceptor and donor residue whose electrostatic contribution to the stabilization of these compounds is remarkable (Figure S13). In fact, together with Ser61 (S3), it is responsible for the low inhibitory activity found for Ald derivatives containing THQ as **R**^1^.

Similarly, for **Ald-10**, one of the most potent covalent inhibitors identified (IC_50_: 3.4 ± 0.5 μM), an interaction pattern characterized by contact with His162, a member of the CZP catalytic triad, was found. Although the **R**^1^ hydroxymethyl substituent of this Ser derivative is small and lacks basic character, its interaction with the imidazolium group of His162 strongly favors the formation of the encounter complex, while the analysis of the docking and MM-MD results revealed a stable H-bond interaction throughout the simulation between the -OH of the **R**^1^ and His162 residue (Figures S13 and S14). This interaction also seems to favor the stabilization of the corresponding WH by orienting and positioning it closer to the catalytic AA Cys25. Indeed, despite not possessing a favorable anchor point at **R**^3^, **Ald-20**, also derived from Ser at **R**^1^, showed moderate inhibitory activity (IC_50_: 7 ± 4 μM). Although these results are not evident from the computed Δ$G_{bind}$ values (Figure S15), the preferential orientation and stabilization of the WH could imply an improvement in ligand reactivity, an effect that cannot be assessed by classical computational design strategies. This hypothesis is also supported by the null activity of **Ald-2**, **Ald-8** and **Ald-11**, three **Ald-10** analogues of similar size but lacking relevant contacts in **R**^1^ with His162.

Furthermore, Ser64 and Asp161 were identified as being responsible for the CZP preference for inhibitors bearing basic and/or aromatic groups oriented towards S1/S1’, with three compounds including a basic side chain on **R**^1^ among the most active ones.

**Figure 9:**
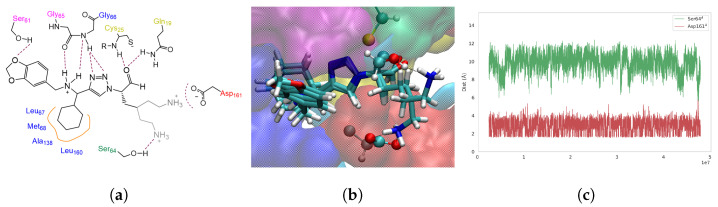
(**a**) Interaction pattern identified for **Ald-6**:**CZP**. The alternative poses of the **R**^1^ substituent are represented (gray). (**b**) MM-MD snapshots of **Ald-6** (licorice), highlighting the alternative poses of its **R**^1^ when interacting with Ser64 and Asp161 (CPK). (**c**) Distance plots of **R**^1^-*n*-butylamino of **Ald-6** with CZP-Asp161 and Ser64.

The docking pose and dynamic behavior of **Ald-6** (IC_50_: 3.3 ± 0.3 μM, Figure 9), synthesized from the AA Lys to introduce the **R**^1^ substituent, always excelled over the rest of the analogues, with the flexible and elongated *n*-butylamino group able to optimize electrostatic interactions with Asp161 (S1’) when complexed to **CZP** (Figure S13). As discussed above, this AA is located at a distant edge of the binding site. This encouraging result was also reflected in classical molecular dynamics studies complemented by binding free energy analysis, as this complex stood out from the other aldehyde derivatives due to its favorable Δ$G_{bind}$, with a difference of about 7 kcal·mol^−1^ compared to the second best Ald (Figure S15), mainly attributed to an EEL contribution.

The importance of Asp161 is also reflected for **Es-15** (Figure S16) and **Ald-4** (Figure S17), since the encounter complex formation during the molecular docking studies is favored by the presence of this anionic AA. However, the larger size and lower flexibility of the propylguanidinium group of **Es-15** and the lower extension of the methylimidazole of **Ald-4** prevent this contact from remaining constant over time, as observed in the MM-MD. The presence of an additional contact point with Ser64 seems to be responsible for the high inhibitory activity of **Es-15** (Figure S13), leading to an optimized encounter complex that results in the non-covalent inhibition of the enzyme. On the other hand, inhibitors including an acid moiety as **R**^1^ were not active at all, with **Ald-3** constituting a representative example, highlighting the role of Asp161 for ligand recognition.

Finally, Phe, Trp and Tyr derivatives (**Ald-1**, **Ald-12** and **Ald-13**) also showed promising results, although with potencies 12.4, 13.8 and 3.9 times lower than the most active analogues (**Ald-6** and **Ald-10**). The size and bulk of their **R**^1^ groups seems sufficient to establish a possible π–anion interaction with the Asp161 carboxylate [69] (Figure S18a), although the energetic contribution of this contact is not accounted in the computed docked energies since it is not parameterized in the scoring function used. More detailed tests involving quantum studies and/or hybrid QM/MM-MD studies are required to further explore this hypothesis. In the particular case of **Ald-13**, its higher potency compared to the other two **R**^1^-aromatic derivatives seems to be justified by the additional anchor point with Ser64, which also reinforces the role of this AA for the stabilization of CZP:inhibitor complexes within S1 (Figure S18b).

**Figure 10:**
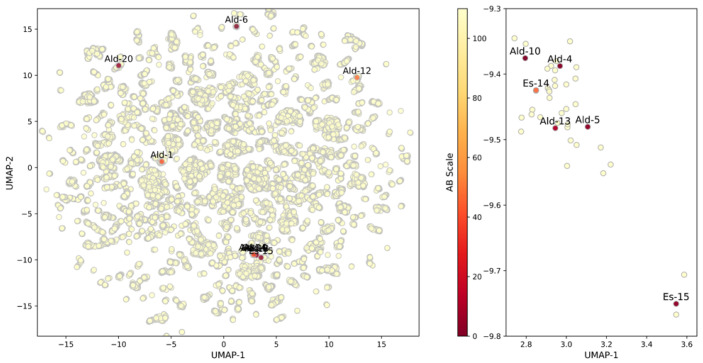
Chemical space of the triazole-based derivatives feasible to be synthesized with the biologically evaluated ones colored according to their IC_50_ against CZP. The subplot on the right shows a magnification of the most populated cluster.

When the bioactivity of the studied compounds was plotted in the context of the explored chemical space [70] (Figure 10), one main region containing the highest proportion of active CZP inhibitors was found. As can be seen in its magnification (Figure 10, right), a significant number of combinations with expected high potency have not been synthetically produced as part of this initial research effort, although the methodological implementation should be straightforward based on the principle of similarity [70,71,72,73,74].

### 2.4. Selectivity Assays: Screening against hCatL

In view of the homology between CZP and hCatL, it is crucial to evaluate the selectivity of CZP inhibitors with respect to the potential unwanted hCatL inhibition, since this may directly affect the in vivo safety profile of antichagasic agents. In this respect, the inhibitory activity against hCatL of the most potent triazole-based CZP inhibitors was evaluated at 100 μM in triplicate. As for the CZP assays, E-64 was used as positive control.

All the evaluated compounds inhibited hCatL activity in less than 26% at 100 μM (Table 2). Therefore, **Es-14**, **Es-15**, **Ald-1**, **Ald-4**, **Ald-5**, **Ald-6**, **Ald-10**, **Ald-12**, **Ald-13** and **Ald-20** can be considered selective CZP inhibitors.

### 2.5. Anti T. cruzi Bioactivity

**Figure 11:**
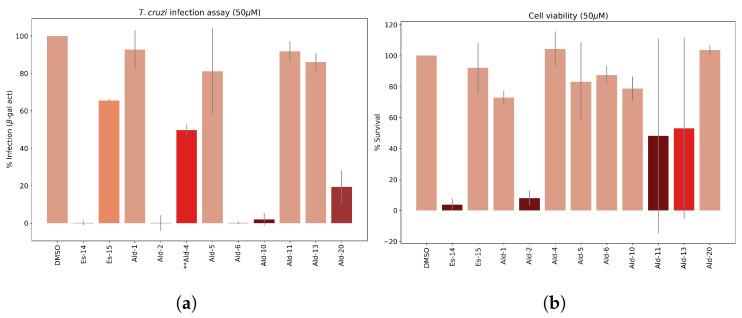
(**a**) Mean ± SD of *T. cruzi* viability quantified in triplicate ([Inh.] = 50 μM) 96 h after cell seeding (72 h p.i.) by β-gal activity assay and expressed as % of remaining infection. ** [Ald-4] = 20 μM. (**b**) Percentage of uninfected Vero cell viability after 96h incubation with triazole-based CZP inhibitors (50 μM), quantified by Alamar blue assay.

Given the significance of attaining a good translation of the CZP inhibitory potency to the corresponding trypanocidal effect, the most potent CZP inhibitors were evaluated on a *T. cruzi* infection model in *Vero* cells, allowing the analysis against both the trypomastigote (extracellular) and amastigote (intracellular) forms. In addition, to further assess the safety of these compounds, cell viability was also measured. Initially, all inhibitors were tested in triplicate at 50 μM, quantifying the percentage of remaining infection after measuring β-galactosidase activity (Figure 11).

**Es-15**, **Ald-4**, **Ald-6**, **Ald-10** and **Ald-20** exhibited acceptable anti-*T. cruzi*/cytotoxicity balance; among which **Ald-6** and **Ald-10** stood out for reducing 97% of infection at the evaluated concentration (Figure 11a). Interestingly, these derivatives also exhibited the highest CZP inhibition, specifically those derived from Lys (**Ald-6**) and Ser (**Ald-10**) as **R**^1^ substituents, as predicted by the in silico studies. These two derivatives also showed low cytotoxicity (Figure 11b) in contrast to the behavior observed for **Es-14** and **Ald-2**, which, despite their high inhibitory potency, showed significantly reduced cell viability.

In a second screening stage, **Ald-6** and **Ald-10** were further assayed at 10 μM. **Ald-10** only reduced *T. cruzi* viability by 38%, while **Ald-6** excelled for its excellent antitrypanosomal activity, with only 7% of parasite survival after 72 h of incubation.

In the final evaluation stage, **Ald-6** was assayed at 1 μM, finding a 27% reduction in *T. cruzi* infection; calculating an IC_50_^*Tc*^ of 3.2 ± 0.4 μM (Figure S20). Notably, the mean IC_50_ values determined for both CZP (IC_50_^*CZP*^ = 3.3 ± 0.1 μM) and *T. cruzi* are statistically equivalent (ANOVA *P*-value: 0.6563), evidencing a complete bioactivity translation, with no biopharmaceutical limitations at the cellular level. In this respect, **Ald-6** exhibited a translation ratio close to 1 (IC_50_^*Tc*^/IC_50_^*CZP*^ ratio: 0.97 ± 0.16) and, as observed in Figure 10, it occupies a different region of the computed chemical space compared to the rest of triazole derivatives assayed, thus defining a region of particular interest for the further design of analogues.

## 3. Materials and Methods

### 3.1. Computer-Aided Studies

#### 3.1.1. Construction of Chemical Libraries

The workflow for the generation of three-dimensional structures of each compound to be evaluated is initiated with a list of SMILES accompanied by an ID or identification code. The SMILES of the 20 triazole derivatives included in the test set were generated using MarvinSketch [75]. Massive libraries of potential new inhibitors were generated employing the Python-based package TidyScreen (https://github.com/alfredoq/TidyScreen, accessed on 1 March 2024). Initially, the SMILES lists of the corresponding building blocks (aldehydes, amines and AA) were extracted from eMolecules [58]. By using the RDKit module [76], the lists were filtered according to criteria based on drug-like properties and excluding those compounds containing metals, non-biocompatible elements or those capable of interfering with the synthetic procedures, such as duplicated reactive groups leading to side products in the different stages of synthesis. In this way, the lists were used to synthesize the triazole derivatives employing the RDKit’s Chem module by means of SMARTS-based reactions [76], combining the different filtered substances by replicating the planned reactions to be executed for their synthesis (diazotransfer, A3 coupling, CuAAC and DIBAL reduction). Once the triazoles were generated, they were subjected to a second round of drug-like-based filtration. From the resulting lists, the chiral centers present in each compound were determined, with the corresponding diastereomers being computed. In-house developed scripts allowed the selection of only those isomers of interest or, alternatively, all of them in case no stereoselectivity was restricted. In the case of the test set, only those exhibiting an (*S*,*S*) configuration were retained. Considering the planned synthetic protocol, the conformation of the chiral α-centre at the N1-triazole position was kept constant, according to the configuration of the α-AA used for their synthesis, varying only that of the Cα at C4. Subsequently, using Dimorphite-DL 1.3 [77], the ionization state of each ligand was evaluated on the basis of an estimated pKa and a defined pH range. Based on this criterion, pH = 5.5–7.4 was set, and all predominant ionization equilibrium forms in this range were retained and considered. Next, RDKit was used to generate a maximum of 100 different conformations, further applying a minimization protocol by considering the Merck Molecular Force Field (MMFF94) [78]. The resulting conformers were ranked on the basis of their computed energy, retaining the lowest energy 3D conformation to initiate ligand preparation procedures. Finally, each ligand was parameterized, generating a coordinate file in the PDBQT format, as required by the docking engine employed (AutoDock suite) [79]. The database containing the building blocks and triazole derivatives evaluated are available for download at https://github.com/alfredoq/Triazole_based_TCI_2024_paper (accessed on 14 March 2024), and a step-by-step tutorial to build and reproduce the libraries is detailed in section “Preparation of a Virtual Chemical Library for vHTS campaigns” at https://github.com/alfredoq/TidyScreen (accessed on 1 March 2024).

#### 3.1.2. CZP Three-Dimensional Structure

The CZP structure deposited under the code PDB ID 3IUT [22] was used as receptor template and was subjected to optimization procedures and further parameterized for molecular docking procedures. In this respect, in a first instance, the missing disulfide bonds were added (Cys22-Cys63, Cys56-Cys101 and Cys155-Cys203), and the structures of the ligands and additives present in the crystallographies were deleted. Considering that many studies claim that the Cys25-His162 pair of the catalytic triad would be mostly in their ionized states, both structures were parameterized so as to generate the corresponding ion pair Cys25S^−^-His162H^+^. The whole preprocessing was performed using ADT v4.2 [79], which also included the addition of polar hydrogen atoms and assignment of Gasteiger charges to the macromolecular structure. The processed protein structure was finally saved as a PDBQT file and stored for later use.

#### 3.1.3. Molecular Docking

The AutoDockGPU 4.2.6 [80] software was employed to accomplish molecular docking studies, running 100 docking runs using a Lamarckian genetic algorithm (LGA) to sample the ligands’ conformational space. The corresponding affinity grids were generated using a 40 × 42 × 60 Å box centered on Cys25 and further extended in order to include the entire active site of CZP. A grid spacing of 0.375 Å was used to calculate the affinity points. The docking results were filtered by extracting the lowest energy and highest cluster enrichment docking poses satisfying the considered pharmacophoric contacts: H-bond interactions (default distance cutoff: 3.7 Å) with Gln19, Ser61, Gly65 and Gly66. Clustering of the resulting docked poses was performed using the built-in functionality of AutoDock-GPU, allowing an RMSD tolerance of 2.0 Å, handling symmetry in the corresponding calculation and saving all computed clusters.

#### 3.1.4. MM-MD and Free Energy Analysis

Molecular dynamic (MM-MD) simulations were performed under explicit solvent conditions using the AMBER22 software package. Ligand and receptor parameters were assigned from the GAFF2 and ff14SB force fields, respectively; with the system preparation and parametrization being performed using the tLeap module of AMBER22 package [81]. The simulation trajectories were obtained by applying periodic boundary conditions, constructing octagonal boxes of explicitly pre-equilibrated TIP3P water molecules, with a minimum distance of 16 Å between the edges of the box and the solute. To obtain the corresponding trajectories, a previously validated MD workflow was applied using the AMBER22 pmemd.cuda module. First, a double-stage minimization step, in which the water molecules were initially minimized with the restricted solute, followed by a minimization of the whole system (10,000 steps for each stage), was carried out. Next, a heating phase was applied, thermostatizing the system from 0 to 300 K for 500 ps, followed by an equilibration phase for an additional 5 ns. Finally, the equilibrated systems were subjected to production runs (100 ns) obtained under constant pressure and temperature conditions, using a time step of 2 fs. The convergence of the simulation was checked by structural and energetic inspection using the cpptraj module of AMBER22, complemented with in-house Python scripts developed exclusively for this purpose. Afterwards, global and per-residue energy analyses of each MM-MD simulation were performed using the MMPBSA.py script [82,83,84], which were applied over the entire trajectory (100 ns), with individual snapshots sampled every 10 frames.

#### 3.1.5. Molecular Visualization

Snapshot visualization of docking poses, structural properties and protein–ligand complexes were calculated and represented using VMD v1.9 software [85]. Most of the 3D-depictions included throughout the document were also rendered from VMD. Schematic diagrams of intermolecular interactions were generated from the results obtained with ProLIF [86] and LigPlot [87].

#### 3.1.6. Computational Infrastructure

All the in silico studies and analyses were carried out using computational resources provided by the MedChem Lab, Departamento de Ciencias Farmacéuticas, Facultad de Ciencias Químicas, Universidad Nacional de Córdoba (FCQ-UNC, Córdoba, Argentina) headed by Prof. Mario A. Quevedo; and at the Centro de Computación de Alto Desempeño (CCAD, UNC [88]), Sistema Nacional de Computación de Alto Desempeño, of the Ministerio de Ciencia, Tecnología e Innovación (SNCAD, MinCyT), particularly in the Mendieta and Serafín clusters.

### 3.2. Synthetic Procedures

#### 3.2.1. Chemistry

The synthetic procedures were carried out at the Laboratory of Organic Synthesis, Sustainable Chemistry for Metals and Molecules Division, Department of Chemistry, KU Leuven (Belgium), headed by Dr Wim Dehaen. All chemicals were purchased from Acros Organics (Geel, Belgium), Alfa Aesar (Karlsruhe, Germany), Fluorochem (Glossop, UK), Merck (Darmstadt, Germany) and TCI Europe (Zwijndrecht, Belgium) and used as is without further purification. Dry diethyl ether, THF and toluene were obtained using an M-Braun SPS-800 system. Moisture sensitive reactions were carried out under nitrogen or argon atmosphere using flame dried glassware. A CEM-Discover microwave reactor was employed for the MW-assisted procedures. Reaction conversion was monitored via TLC analysis using MilliporeSigma™ Silica Gel 60 F254 Coated Aluminum-Backed TLC Sheets (Sigma, St. Louis, MO, USA). Column chromatography was performed via standard column chromatography, employing 70–230 mesh silica 60 (Acros) as the stationary phase. ^1^H NMR spectra were recorded on Bruker Avance 300 (300 MHz) or Bruker Avance 400 (400 MHz) spectrometers. The ^13^C NMR spectra were recorded on a Bruker Avance 400 (101 MHz working frequency, Bruker, Billerica, MA, USA). The NMR samples were dissolved in a corresponding deuterated solvent, and chemical shifts (δ) were reported in parts per million (ppm) and referenced relative to the deuterated solvent signal [89]. The FTIR-spectra were recorded on a Bruker Vertex 70 spectrometer (Bruker, Billerica, MA, USA), and OPUS 8.5 software was used to analyze the recorded spectra. The high-resolution mass spectra were acquired on a quadrupole orthogonal acceleration time-of-flight mass spectrometer (Synapt G2 HDMS, Waters, Milford, MA, USA). The samples were infused at 3 μL/min and spectra were obtained in positive or negative ionization mode with a resolution of 15,000 (FWHM—full width at half maximum) using leucine enkephalin as a lock mass. The full characterization data of intermediates and evaluated compounds are available within the Supplementary Materials.

#### 3.2.2. Azides (Az)

Imidazole-1-sulfonyl azide hydrogen sulfate (ISA·H_2_SO_4_) was synthesized according to Method B reported by Potter et al. [52] To a cooled and stirred suspension of sodium azide (20 mmol) in dry EtOAc (20 mL) under a N_2_ atmosphere, sulfuryl chloride (20 mmol) was added dropwise over 10min, after which the mixture was warmed to room temperature and stirred for 17 h. After recooling at 0 °C, imidazole (40 mmol) was added. The thick suspension was stirred for 3 h at 0 °C and then basified with saturated NaHCO_3_. The organic layer was washed, dried, cooled (0 °C) and placed under a N_2_ atmosphere. H_2_SO_4_ (conc., 20 mmol) was added dropwise over the course of 5 min, after which the resulting mixture was gradually warmed to room temperature. After 45 min, the precipitate formed was filtered and dried to yield pure ISA.H_2_SO_4_ (16.5 mmol, 4.5 g, 83%).

General procedure **A** for the synthesis of α-azido esters (Az-1 to Az-15). This method was adapted from the one reported by Goddard-Borger and Stick [53]. To a round-bottom flask equipped with a magnetic stirring bar, the corresponding α-amino ester (1 eq), K_2_CO_3_ (3.4 eq), CuSO_4_·5H_2_O (0.01 eq) and ISA.H_2_SO_4_ (1.2 eq) were added and suspended in methanol (5 mL). The reaction mixture was stirred overnight at room temperature for the specified time (14–15 h) and concentrated under reduced pressure. The crude was resuspended in EtOAc and extracted twice with 1 M HCl, washed with water, dried over MgSO_4_, filtered and concentrated in vacuo. Flash chromatography was carried out when necessary to afford the desired product.

#### 3.2.3. CuAAC

General procedure **B** for the synthesis of 1,4-disubstituted 1,2,3-triazole derivatives (Es-1 to Es-24). This method was adapted from the one reported by Zsabka et al. [90] In a round-bottom flask equipped with a magnetic stirring bar, the corresponding alkyne (1 eq) and azide (1 eq) were dissolved in DCM (0.05 M). A solution of sodium ascorbate (2 eq) in H_2_O (0.1 M) was added as a second phase; followed by the dropwise addition of CuSO_4_·5H_2_O (0.02 eq) in H_2_O (5 mM) and DIPEA (3.5 eq). The resulting mixture was stirred vigorously at room temperature until full conversion, as indicated by TLC. The resulting mixture was extracted three times with H_2_O:CHCl_3_, and the combined organic layers were dried over MgSO_4_. The solvents were evaporated under reduced pressure, and the residue was purified (when necessary) to afford the corresponding 1,4-disubstituted 1,2,3-triazole derivative.

#### 3.2.4. Aldehydes

General procedure **C** for the synthesis of aldehyde derivatives (Ald 1-23). This method was adapted from the one reported by Wood et al. [57]. To an oven-dried round-bottom flask equipped with a magnetic stirring bar, the ester derivative (1 eq) was added and dissolved in dry THF (0.2 M) under inert atmosphere. The solution was cooled in a dry ice/acetone cooling bath, followed by the dropwise addition of diisobutylaluminium hydride (DIBAL, 3 eq) in THF. After full conversion, as indicated by TLC (2–5 h), the temperature was raised to 0 °C, and the reaction was quenched by the slow addition of acetic acid (15 eq). A saturated aqueous potassium sodium tartrate solution was added, and the reaction mixture was stirred for 20 min. The resulting mixture was extracted five times with EtOAc, and the combined organic layers were dried over MgSO_4_. The solvents were evaporated under reduced pressure, and the residue was purified by column chromatography to afford the corresponding aldehyde derivative.

### 3.3. Biological Assays

#### 3.3.1. Screening against CZP

The previously reported methods were followed to evaluate the in vitro activity against CZP [91,92,93,94]. Allison Doak and Prof. Brian Shoichet (University of California San Francisco, San Francisco, CA, USA) generously provided recombinant CZP (*cruzain*). The enzyme activity was measured by monitoring the cleavage of the fluorogenic substrate Z-FR-AMC at 25 °C. Unless stated otherwise, assays were performed using 2.5 μM of substrate ($K_{M}$ = 0.5 ± 0.1 μM) and 0.5 nM CZP in a 0.1 M sodium acetate buffer, pH 5.5, 0.01% Triton X-100 and 2 mM β-mercaptoethanol. DMSO and 1 μM E-64 were employed as negative and positive controls, respectively, in all assays. Fluorescence was monitored at 340/440 nm (excitation/emission) over time in a Biotec 87 Synergy 2 fluorimeter at the Multiuser Laboratory of the Biochemistry and Immunology Department (UFMG, Belo Horizonte, Brazil). All assays were performed in triplicate in two independent experiments.

Initial screening. For the initial screening against CZP, compounds at 100 μM were tested against CZP with and without a 10min pre-incubation with the enzyme. The fluorescence was monitored for 10 min at 5 s intervals. DMSO and E-64 were employed in all assays as negative and positive controls, respectively. Initial velocities of the negative control (DMSO) were compared to those in the presence of compounds to calculate the percentage of inhibition. The reported values correspond to the mean and standard error of the mean (SEM) of experiments performed in triplicate in two independent experiments.

IC_50_ determination. Compounds in at least eight distinct compound concentrations (400 μM, 100 μM, 25 μM, 6.25 μM, 1.5 μM, 0.4 μM, 0.1 μM and 0.025 μM) were incubated with CZP for 10 min, and once the substrate was added, the fluorescence was monitored for 10 min at 5 s intervals. IC_50_ curves were obtained from a non-linear fit of the inhibition values versus the logarithmic concentration of the compounds. The reported IC_50_ values are the mean and SEM of two independent measurements performed in triplicate.

Aggregation Assays. To evaluate detergent sensitivity, CZP was pre-incubated with compounds at their IC_50_ for 10 min at room temperature in varying concentrations of Triton X-100 (0%, 0.1% and 0.01%). Additionally, compounds at their IC_50_ were incubated for 10min with BSA (4 mg/mL, Sigma-Aldrich, St. Louis, MO, USA) followed by 10 min incubation in the presence of CZP, and upon the addition of substrate, the enzyme activity was monitored. For comparison, the four conditions were assayed in parallel. Aggregation was excluded when the difference in inhibition percentages between the different conditions was less than 20%.

Jump dilution assay. For CZP at 100-fold, its final assay concentration was incubated with the inhibitor(s) at 10-fold its respective IC_50_ value for 30 min in a volume of 2 μL in a 96-well plate. This mixture was diluted 100-fold with an assay buffer containing a 5 μM Z-FR-AMC to a final volume of 200 μL, resulting in a standard concentration of enzyme and 0.1 times the IC_50_ value of inhibitor. Fluorescence intensities were monitored continuously for 2 h.

Mechanism of inhibition. To determine the mechanism of inhibition and $K_{i}$ values of **Es-15**, CZP activity was monitored at a minimum of six different substrate concentrations (0.3–20.0 μM) and five inhibitor concentrations (from 0.25× to 2× IC_50_) and in the absence of the compound. The results were calculated by nonlinear (Michaelis–Menten) and linear regression (Lineweaver–Burk plots). The effect of the inhibitor concentration on the $K_{Mapp}$ and its $K_{i}$ was evaluated by the general linear F-test.

#### 3.3.2. CatL Screening

The recombinant human cathepsin L (hCatL) (Sigma-Aldrich) was activated prior to the assays. First, 10 ng/μL of the enzyme was incubated in the activation buffer (50 mM MES, pH 6, 1 mM EDTA, 5mM DTT) for 30 min at 37 °C. hCatL activity was measured based on the cleavage of the fluorescent substrate Z-FR-AMC in a Biotec 87 Synergy 2 fluorimeter at the Multiuser Laboratory of the Biochemistry and Immunology Department (UFMG, Brazil). The assays were executed in 96-well round bottom black plates in 50 mM MES buffer, pH 6, containing 1 mM EDTA, 5 mM DTT, 0.001% Triton-X100, 5 pg/μL enzyme and 25 μM Z-FR-AMC at 37 °C. The triazole-based derivatives were tested against hCatL, at 100 μM, with a 10 min pre-incubation with the enzyme. The initial velocities of the DMSO control were compared to those in the presence of the compounds. Assays were executed in triplicate in two independent experiments, and enzyme activity was measured for 20 min.

#### 3.3.3. *T. cruzi* Inhibition Assay

To perform the assay, the drug screening method reported by Buckner et al. was adapted [95,96]. Vero cells were seeded at 1 × 10^4^ per well on 96-well culture plates in 100 μL MEM-5% FBS. Tulahuen trypomastigotes overexpressing the *E. coli*β-galactosidase protein were distributed in triplicates on the plate. After a 2 h incubation at 37 °C, the culture media were extracted and replaced with fresh MEM-5% FBS with 1 μM, 10 μM or 50 μM solutions of the triazole-based inhibitors, and the plate was incubated another 72 h at 37 °C. Ninety-six hours post infection, the cell culture media were removed and cells and intracellular amastigotes were lysed in 100 μL lysis buffer (25 mM Tris pH 8, 2 mM EDTA, 2 mM DTT, 1% Triton X-100, 10% glycerol in ultrapure MQ water) for 10 min at 37 °C. Then, 100 μL 2× reaction buffer (200 mM sodium phosphate pH 8, 2 mM MgCl_2_, 100 mM 2-mercaptoethanol and 1.33 mg.mL^−1^ *o*-nitrophenyl-β-galactoside (ONPG)) was added and the reaction was allowed to proceed until a yellow color developed (1–2 h at 37 °C). The absorbance at 420 nm was measured in a Synergy HTX multi-mode microplate reader (Biotek Instruments, Winooski, VT, USA) and normalized to the value obtained for the infection in the absence of inhibitors. To determine its IC_50_, **Ald-6** was evaluated at nine different concentrations (0.098, 0.195, 0.39, 0.78, 1.56 3.13 6.25, 12.5 and 50.0 μM), following the same protocol. Each infection condition and inhibitor were tested in triplicates in four independent experiments.

#### 3.3.4. Cytotoxicity Assay

To evaluate the viability of Vero cells, the Alamar blue method was followed [96]. First, 10^4^ Vero cells/well in 100 μL MEM-10% FBS were seeded in 96-well culture plates. After 24 h, 50 μM inhibitor solution was added to cell monolayers and incubated for 96 h. After incubation, resazurin solution (final concentration 10 μg·mL^−1^) was added to each well, and fluorescence was measured after 2 h in a Synergy HTX multi-mode microplate reader (Biotek Instruments, Winooski, VT, USA) using the 530–560 nm emission and 590 nm excitation filters. Each condition was tested in triplicate and in at least three independent experiments.

#### 3.3.5. Statistical Analysis

All statistical analyses were performed with GraphPad Prism 6 and complemented with Python-based scripts.

## 4. Conclusions

The present work has been conducted in the context of CD, which remains a neglected disease requiring continuous scientific efforts to develop potent and safe drugs. The validation of CZP as a therapeutic target for *T. cruzi* and the renewed interest in TCI as pharmacotherapeutic alternatives stand out. Building on the previous research, we have further demonstrated the potential of the triazole scaffold to develop peptidomimetic antichagasic agents targeting CZP. The versatility of triazole chemistry has enabled us to explore a vast chemical space through in silico methodologies, with good predictions of CZP inhibition, which were largely validated through complementary experimental efforts.

In this study, we elucidated a comprehensive structure–activity relationship (SAR) concerning the binding mode of potent and selective CZP inhibitors. Our results confirm that the chemical space for effective bioactivity translation from CZP to *T. cruzi* is indeed narrow but compatible with adequate selectivity over hCatL.

Carefully designed screening campaigns, coupled with robust criteria for sampling the compound regions to be experimentally explored, enhance the chances of identifying candidates with the desired trypanocidal effect. Following this approach, we identified a potent, safe and selective antichagasic candidate (**Ald-6**) that merits further advancement to more complex preclinical bioactivity assessments, such as in vivo models. Additionally, we delineated a confined chemical space where the likelihood of identifying clinically useful CZP TCI is maximized.

Overall, in agreement with the recent findings, we emphasize the critical need to include cell-based *T. cruzi* infection assays alongside classical CZP inhibition evaluations in the preclinical development pipeline of antichagasic agents.

As ongoing work related to the presented research efforts, additional WH groups are envisioned that not only may provide an adequate reactivity for Cys25 labeling but may also contribute to the specific recognition of the inhibitor within the CZP catalytic site. In this respect, the WH groups reported in the present work (i.e., Es and Ald) are feasible to be further modified towards reactive groups that provide additional intermolecular recognition contributions.

## Figures and Tables

**Table 1:** Structural, synthetic and enzymatic data of triazole-based **Es** and **Ald** derivatives.

R^3^	R^1^	Es ^*a*^	Yield ^*c*^	% CZP inh. *^e^*	IC_50_ (μM) ^*f*^	Ald ^*a *^	Yield ^*d*^	% CZP inh. *^e^*	IC_50_ (μM) ^*f*^
BDX	Phe	**Es-1** ^*b*^	89%	15 ± 2	n.d.	**Ald-1**	36%	64 ± 5	41 ± 4
	Val	**Es-2**	85%	9 ± 7	n.d.	**Ald-2**	53%	55 ± 3	n.d.
	Glu	**Es-3**	50%	8 ± 7	n.d.	**Ald-3**	21%	8 ± 2	n.d.
	His	**Es-4**	89%	5 ± 4	n.d.	**Ald-4**	42%	83 ± 2	5 ± 2
	Leu	**Es-5**	90%	5 ± 2	n.d.	**Ald-5**	32%	64 ± 2	7 ± 2
	Lys	**Es-6**	48%	9 ± 2	n.d.	**Ald-6**	51%	81 ± 2	3.3 ± 0.3
	Met	**Es-7** ^*b*^	84%	9 ± 8	n.d.	**Ald-7**	59%	42 ± 7	n.d.
	Ala	**Es-8**	84%	8 ± 7	n.d.	**Ald-8**	38%	8 ± 4	n.d.
	Phg	**Es-9**	82%	6 ± 5	n.d.	**Ald-9**	37%	11 ± 3	n.d.
	Ser	**Es-10**	87%	47 ± 6	n.d.	**Ald-10**	56%	87 ± 1	3.4 ± 0.5
	Thr	**Es-11**	65%	0 ± 2	n.d.	**Ald-11**	44%	58 ± 4	n.d.
	Trp	**Es-12**	68%	15 ± 2	n.d.	**Ald-12**	58%	56 ± 7	45.4 ± 0.3
	Tyr	**Es-13**	51%	8 ± 1	n.d.	**Ald-13**	43%	83 ± 2	13 ± 5
	Cys	**Es-14**	73%	53 ± 4	n.d.	**Ald-14**	-	-	-
	Arg	**Es-15**	43%	86 ± 1	6.8 ± 0.3	**Ald-15**	-	-	-
THQ	Phe	**Es-16** ^*b*^	76%	13 ± 6	n.d.	**Ald-16**	55%	34 ± 5	n.d.
	His	**Es-17**	54%	6 ± 3	n.d.	**Ald-17**	56%	38 ± 6	n.d.
	Met	**Es-18** ^*b*^	60%	0 ± 6	n.d.	**Ald-18**	43%	16 ± 4	n.d.
	Phg	**Es-19**	51%	0 ± 3	n.d.	**Ald-19**	49%	3 ± 10	n.d.
	Ser	**Es-20**	77%	5 ± 4	n.d.	**Ald-20**	38%	83 ± 3	7 ± 4
	Thr	**Es-21**	71%	0 ± 7	n.d.	**Ald-21**	37%	8 ± 7	n.d.
	Trp	**Es-22**	58%	1 ± 6	n.d.	**Ald-22**	50%	55 ± 9	n.d.
	Tyr	**Es-23**	51%	1 ± 14	n.d.	**Ald-23**	44%	20 ± 4	n.d.
	Arg	**Es-24**	28%	6 ± 1	n.d.	**Ald-24**	-	-	-

^*a*^ The **R**^2^ for all of them is c-Hex. ^*b*^ Es-derivatives obtained from L-AA-OEt, while the remaining were synthesized from L-AA-OMe. Yields of ^*c*^ CuAAC and ^*d*^ DIBAL-mediated reduction steps. ^*e*^ The initial screening against CZP was performed in triplicate at 100 μM (% CZP inhib.) with 10’ pre-incubation of the enzyme in the presence of the compounds. ^*f*^ IC_50_ are the mean ± SEM of two independent experiments. n.d.: not determined.

**Table 2:** Activity profiles of 1,4-disubstituted 1,2,3-triazole derivatives against CZP and hCatL at 100 μM after 10 min of incubation. * E-64: positive control.

Compd ID	% CZP inhib.	% hCatL inhib.
**Es-14**	53 ± 4	0 ± 26
**Es-15**	86 ± 1	26 ± 16
**Ald-1**	64 ± 5	0 ± 21
**Ald-4**	83 ± 2	10 ± 3
**Ald-5**	64 ± 2	24 ± 9
**Ald-6**	81 ± 2	26 ± 5
**Ald-10**	87 ± 1	7 ± 2
**Ald-12**	56 ± 7	0 ± 16
**Ald-13**	83 ± 2	0 ± 22
**Ald-20**	83 ± 3	0 ± 39
**E-64** *	98 ± 2	97 ± 3

## Data Availability

The SMILES of the 20 4FPMK derivatives [46] of the training set were generated using MarvinSketch (https://docs.chemaxon.com/display/lts-europium/marvinsketch.md). The SMILES lists of building blocks were obtained from the eMolecules database (https://www.emolecules.com/). The RDKit (https://www.rdkit.org/) package was employed to construct and filter the libraries of evaluated triazole derivatives, and Dimorphite-DL 1.3 (https://pypi.org/project/dimorphite-dl/) to set their corresponding ionization state. The meeko package (https://pypi.org/project/meeko/) was used to obtain PDBQT files as required for docking studies by AutoDock-GPU (https://github.com/ccsb-scripps/AutoDock-GPU), which was employed at each instance of molecular docking. The protein structure of CZP was retrieved from the Protein Data Bank (PDB ID: 3IUT, https://www.rcsb.org/structure/3IUT, accessed on 28 August 2021). All protein–ligand complex topologies were prepared using the AmberTools22 package as freeware (https://ambermd.org/AmberTools.php), while molecular dynamics simulations were computed using Amber22 (https://ambermd.org/AmberMD.php) through the non-commercial program. Binding free energies were computed using the MMPBSA.py script (https://ambermd.org/). For the visualization and analyses of in silico results, VMD v1.9 software (https://www.ks.uiuc.edu/Research/vmd/), ProLIF (https://github.com/chemosim-lab/ProLIF) and LigPlot (https://www.ebi.ac.uk/thornton-srv/software/LigPlus/) were employed. The spectroscopic data were analyzed by TopSpin 3.6.5 (https://www.bruker.com/en/products-and-solutions/mr/nmr-software/topspin.html), and GraphPad Prism 6 was used for statistical analyses related to biological assays (https://www.graphpad.com/scientific-software/prism/). Most input data and parameters are available for download at https://github.com/alfredoq/Triazole_based_TCI_2024_paper (accessed on 1 March 2024). The full MM-MD trajectories, additional scripts and data are available from the authors upon request.

## Supplementary Materials

The following supporting information can be downloaded at: https://www.mdpi.com/article/10.3390/molecules29174224/s1, Additional experimental details and findings represented or summarized in figures and tables. Synthesis details, characterization, and ^1^H NMR and ^13^C NMR spectra for all precursors and triazole derivatives described and evaluated. Reference [97] is cited in the Supplementary Material.

## Abbreviations

The following abbreviations are used in this manuscript:

Ald	aldehyde
BDX	1,3-benzodioxole
CD	Chagas disease
CP	Cysteine proteases
CZP	Cruzipain
Es	ester
hCatL	human cathepsin L
TCI	Targeted covalent inhibitors
THQ	1,2,3,4-tetrahydroisoquinoline
vHTS	virtual high throughput screening
WH	warhead
4FPMK	tetrafluorophenoxymethyl ketone
