# Supplementary material for: Structure-Aided Computational Design of Triazole-Based Targeted Covalent Inhibitors of Cruzipain
Source: Molecules. 2024 Sep 5;29(17):4224. doi: 10.3390/molecules29174224 (PMC11396839; doi:10.3390/molecules29174224)
Supplement: Supplementary file 1 [file molecules-29-04224-s001.zip › molecules-3083386-supplementary.pdf]

Supplementary material

Structure-Aided Computational Design of Triazole-Based Targeted Covalent Inhibitors of Cruzipain

Juan Pablo Cerutti [1,2], Lucas Abreu Diniz [3], Viviane Corrêa Santos [3], Salomé Catalina Vilchez Larrea [4], Guillermo Daniel Alonso [4], Rafaela Salgado Ferreira [3], Wim Dehaen [2] and Mario Alfredo Quevedo [1,*]

1 Unidad de Investigación y Desarrollo en Tecnología Farmacéutica (UNITEFA-CONICET), Facultad de Ciencias Químicas, Universidad Nacional de Córdoba (FCQ-UNC), Haya de la Torre y Medina Allende, Córdoba 5000, Argentina; jpcerutti@unc.edu.ar

2. Sustainable Chemistry for Metals and Molecules, Department of Chemistry, KU Leuven, Celestijnenlaan 200F, 3001 Leuven, Belgium; wim.dehaen@kuleuven.be

3 Departamento de Bioquímica e Imunologia, Instituto de Ciências Biológicas, Universidade Federal de Minas Gerais, Av. Antônio Carlos 6627, Belo Horizonte 31270-901, Brazil; lucasdiniz1478@gmail.com (L.A.D.); vivicorreasantos@gmail.com (V.C.S.); rafaelasalgadoferreira@gmail.com (R.S.F.)

4 Instituto de Investigaciones en Ingeniería Genética y Biología Molecular (INGEBI-CONICET), Vuelta de Obligado 2490, Ciudad de Buenos Aires 1428, Argentina; vilchez.ingebi@gmail.com (S.C.V.L.); galonso@dna.uba.ar (G.D.A.)

* Correspondence: aquevedo@unc.edu.ar

Additional experimental details and findings represented or summarised in figures and tables.

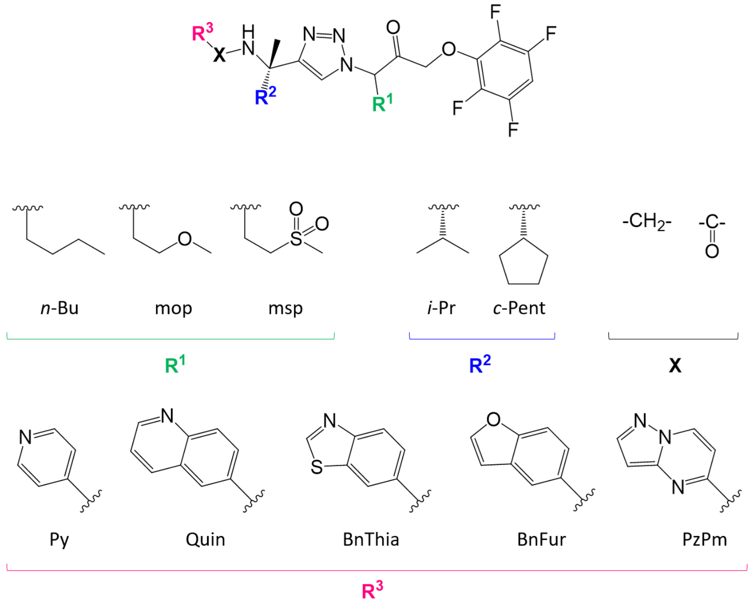

Figure S1. General overview of the 20 triazole-based 4FPMK derivatives reported as CZP inhibitors.[1]

Table S1. Summary of the IDs, chemical structures and IC_{50} against CZP of the 20 triazole-based 4FPMK inhibitors.[1]

**ID**	$\mathbf{R}^1$	$\mathbf{R}^2$	**X**	$\mathbf{R}^3$	$\mathbf{IC_{50}}$ (nM)
**Ts-3**	*n*-Bu	*c*-Pent	CH_2	PzPm	3
**Ts-5**	*n*-Bu	*c*-Pent	CH_2	BnThia	5
**Ts-25**	msp	*c*-Pent	CH_2	BnThia	25
**Ts-27**	*n*-Bu	*c*-Pent	CH_2	Quin	27
**Ts-37**	*n*-Bu	*i*-Pr	CH_2	BnThia	37
**Ts-63**	mop	*i*-Pr	CH_2	BnThia	63
**Ts-75**	*n*-Bu	*i*-Pr	CH_2	PzPm	75
**Ts-124**	mop	*c*-Pent	CH_2	BnThia	124
**Ts-125**	msp	*i*-Pr	CH_2	BnThia	125
**Ts-340**	mop	*i*-Pr	CH_2	Quin	340
**Ts-370**	*n*-Bu	*i*-Pr	CH_2	Quin	370
**Ts-467**	msp	*i*-Pr	CH_2	PzPm	467
**Ts-560**	msp	*i*-Pr	CH_2	Quin	560
**Ts-790**	msp	*c*-Pent	CH_2	BnFur	790
**Ts-880**	msp	*i*-Pr	CH_2	BnFur	880
**Ts-1100**	*n*-Bu	*i*-Pr	CH_2	Py	1100
**Ts-1350**	*n*-Bu	*i*-Pr	C=O	Quin	1350
**Ts-2820**	mop	*c*-Pent	CH_2	PzPm	2820
**Ts-3590**	*n*-Bu	*i*-Pr	C=O	BnThia	3590
**Ts-23000**	*n*-Bu	*i*-Pr	C=O	Py	23000

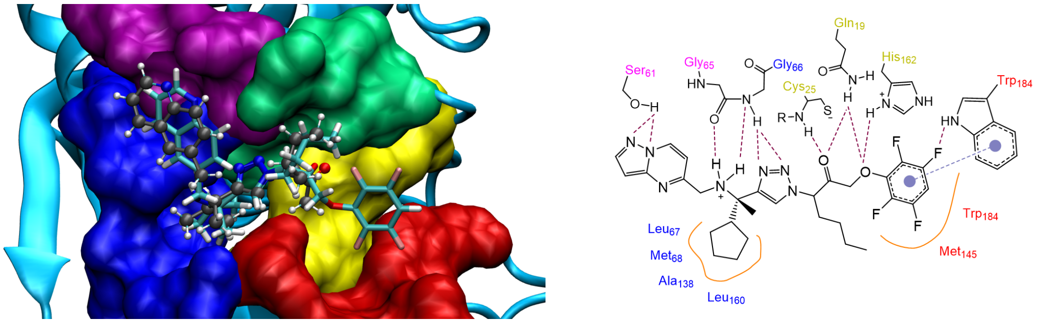

(a) (b)

Figure S2. (a) Overlay of crystallographic covalent-bonded **Ts-370** (PDB 3IUT, CPK, grey) vs. resulting docking pose for **Ts-370** (licorice, cyan) complexed to CZP. (b) 2D-depiction of the interaction pattern identified by MM-MD for **Ts-3**:**CZP** binding complex.

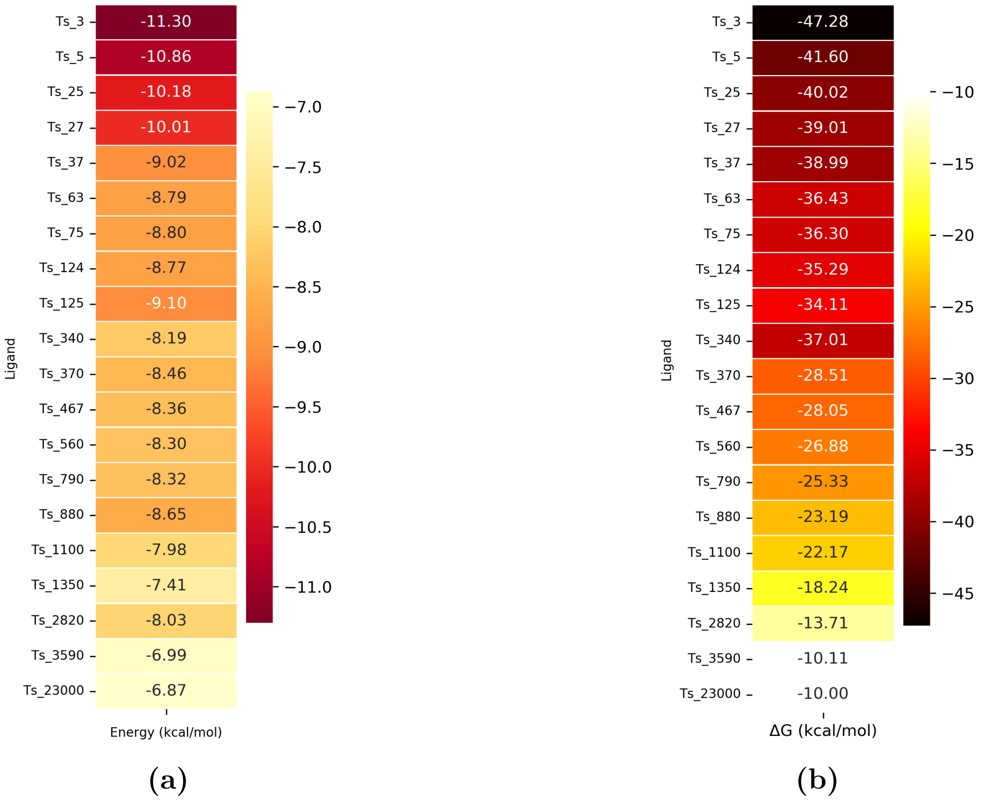

Figure S3. (a) Docking and (b) MMPBSA energetic analysis of the complex formation between CZP and each ligand of the test set, ordered by their $IC_{50}{}^{CZP}$.[1] The energy values corresponding to the docking score (a) and MMPBSA calculation (b) are expressed in kcal/mol.

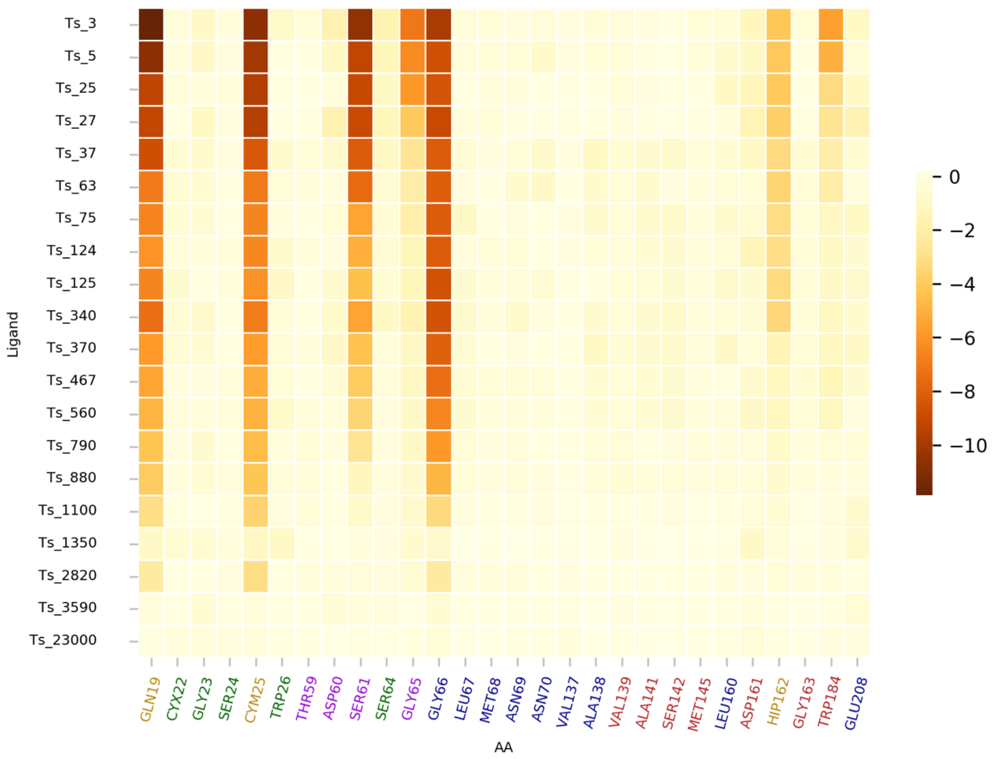

Figure S4. MMPBSA per-residue energetic analysis of the **electrostatic** interactions between CZP and each ligand of the test set. Only those AA constituting the active site of CZP were preselected, and are identified with different colours according to whether they belong to the **S1**, **S2**, **S3** and **S1'** or are part of the **catalytic triad** and **oxyanion hole**.

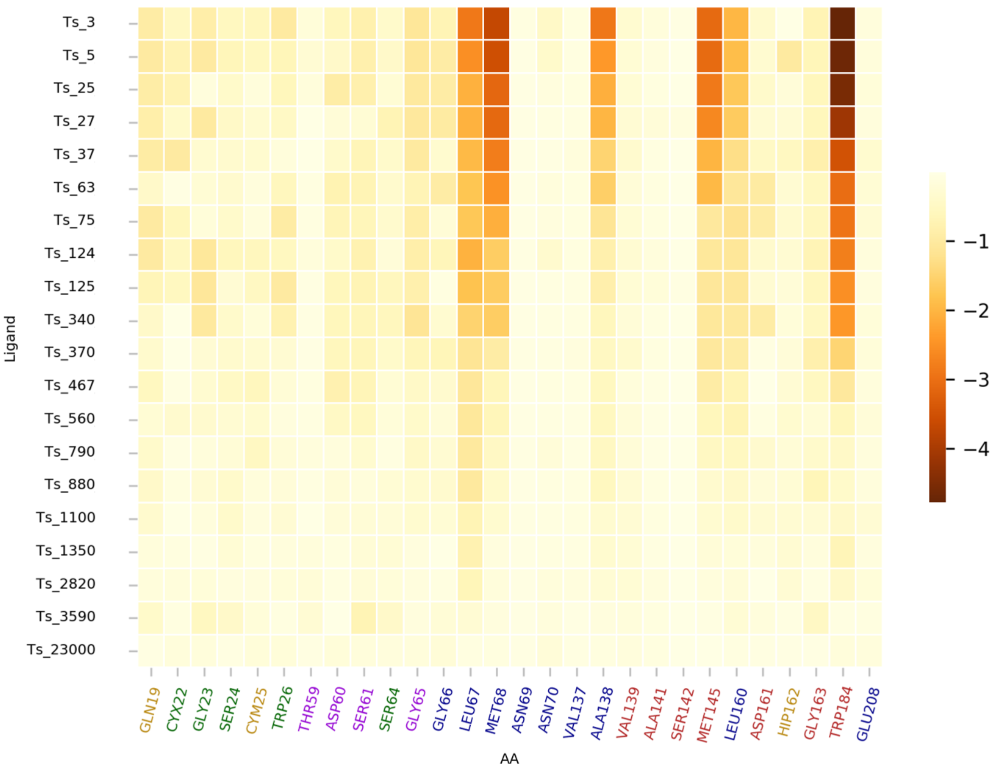

Figure S5. MMPBSA per-residue energetic analysis of the **van der Waals** interactions between CZP and each ligand of the test set. Only those AA constituting the active site of CZP were preselected, and are identified with different colours according to whether they belong to the **S1**, **S2**, **S3** and **S1'** or are part of the **catalytic triad** and **oxyanion hole**.

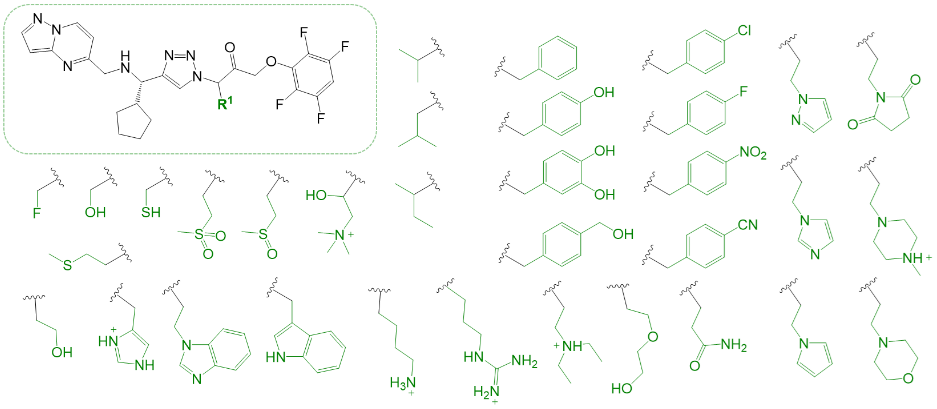

Figure S6. Promising $\mathbf{R}^1$ substituents identified by docking-based vHTS.

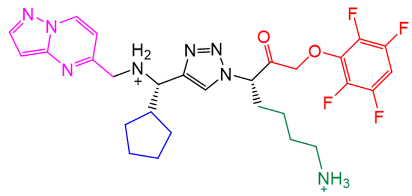

(a)

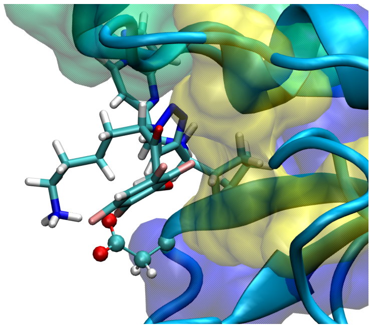

(b)

Figure S7. (a) 2D structure and (b) binding complex resulting from molecular docking pose of **R**[1]**Lys-4FPMK** (licorice) with CZP. **Asp161** is represented in CPK.

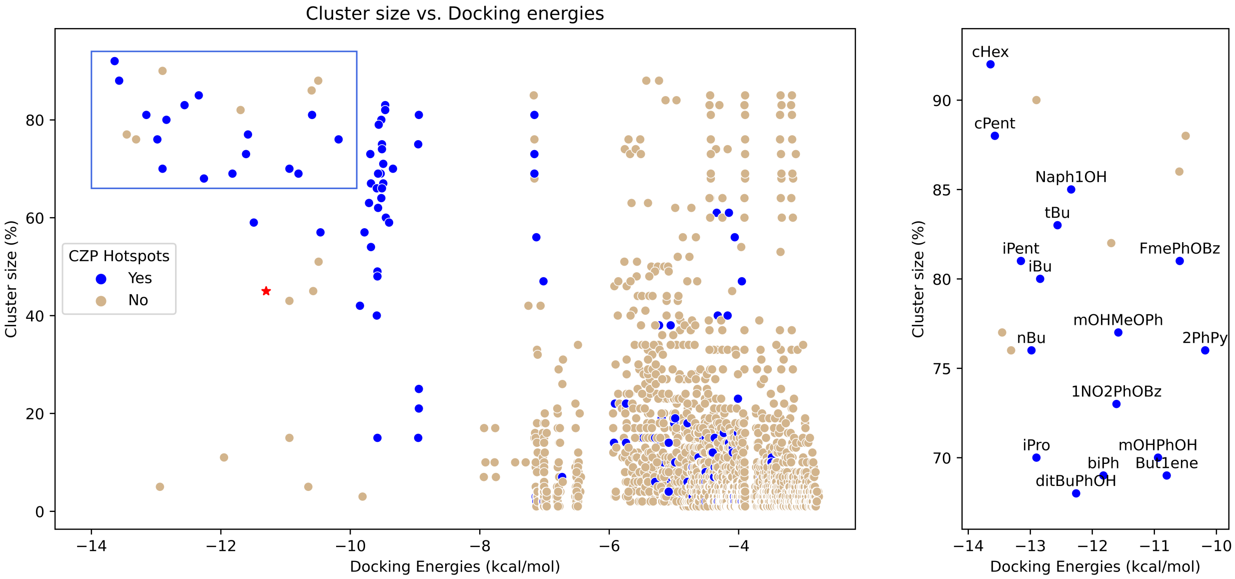

Figure S8. Summary of the docking-based vHTS results focused on $\mathbf{R}^2$ with **CZP**.

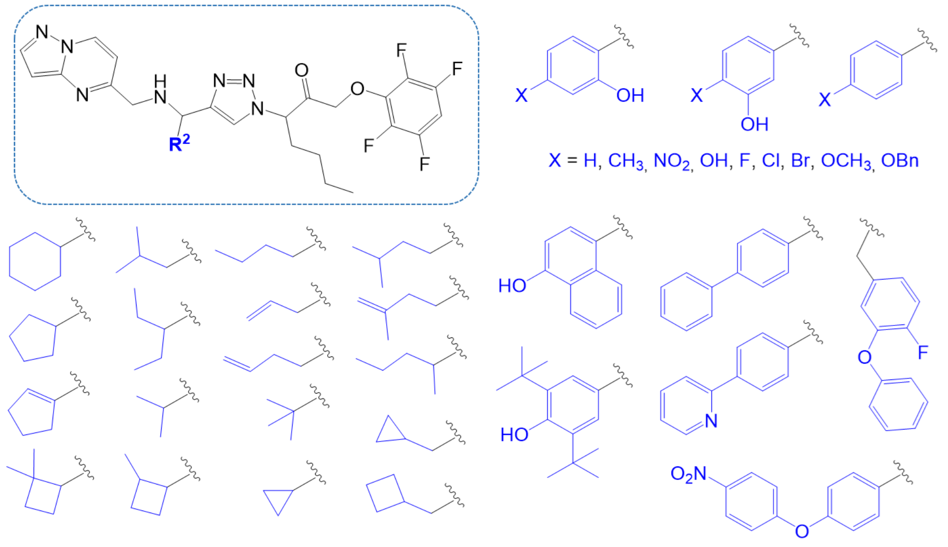

Figure S9. Promising $\mathbf{R}^2$ substituents identified by docking-based vHTS.

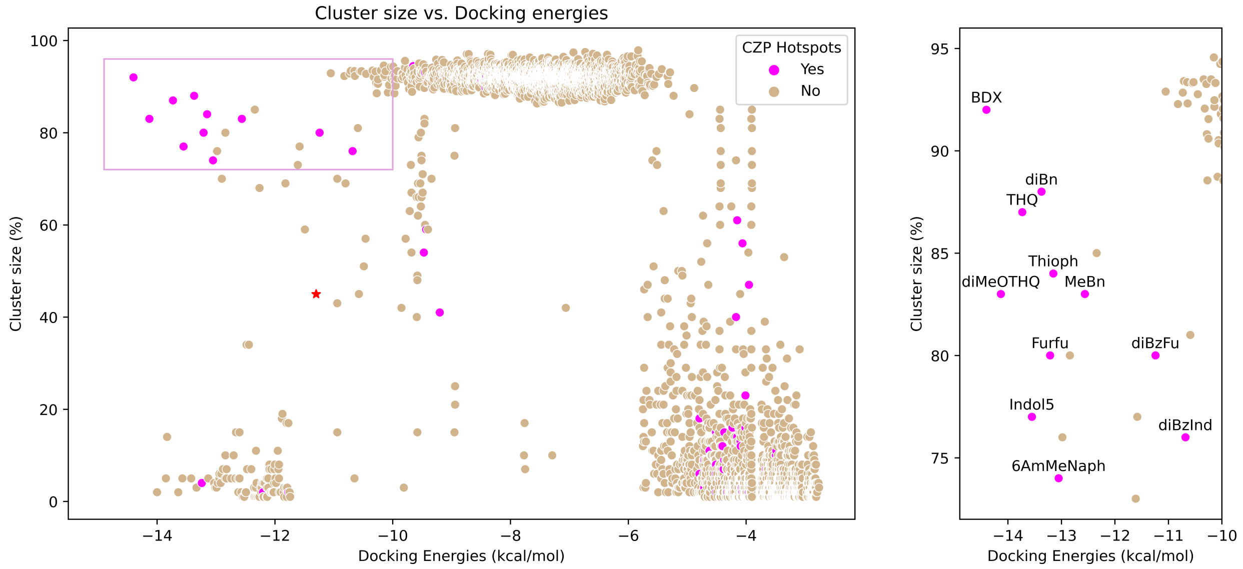

Figure S10. Summary of the docking-based vHTS results focused on $\mathbf{R}^3$ with **CZP**.

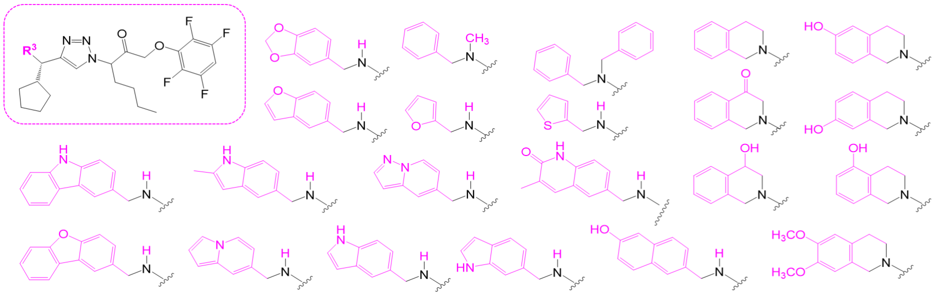

Figure S11. Promising $\mathbf{R}^3$ substituents identified by docking-based vHTS.

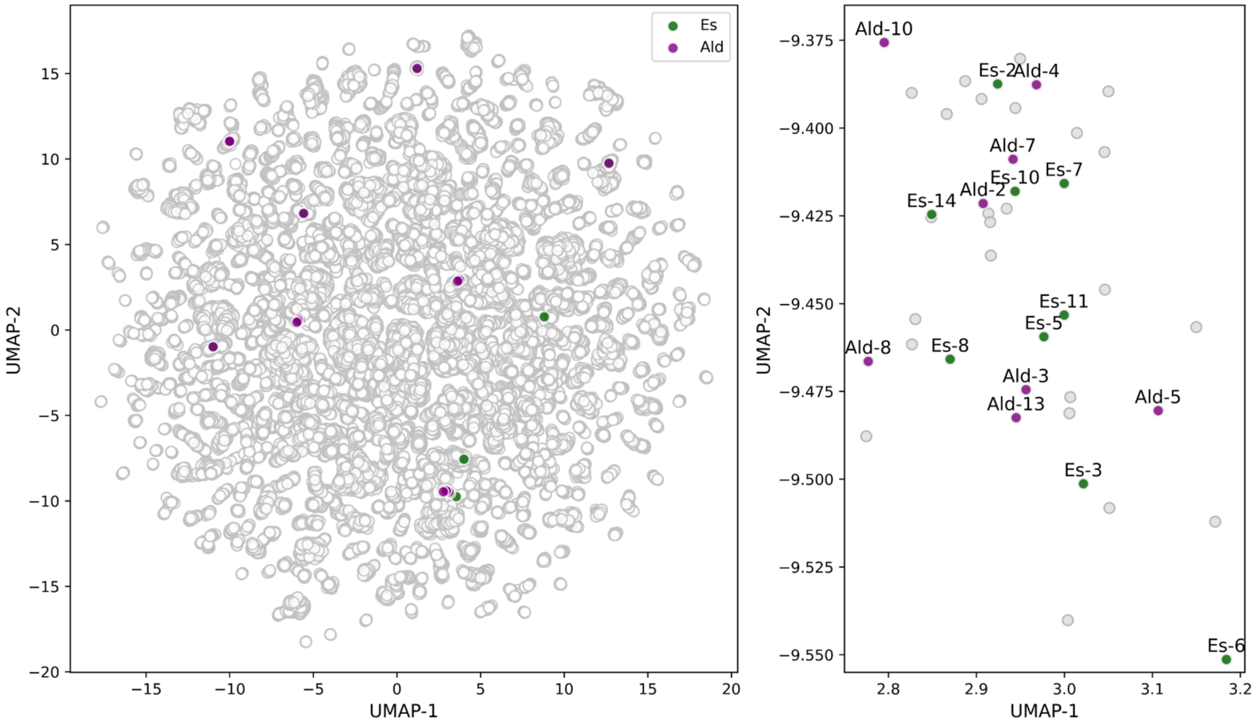

Figure S12. Chemical space of triazole-based **Es** and **Ald** derivatives feasible to be synthesised according to the building blocks of **Az** and **PA** generated *in silico* (n = 77024), highlighting in green (Es) and purple (Ald) those which were obtained and are reported in the present work. Due to their structural similarity, most of the Es and Ald analogues bearing identical $\mathbf{R}^1$, $\mathbf{R}^2$ and $\mathbf{R}^3$ groups occupy very similar coordinates on the map, as shown in the subplot on the right with the one-quadrant maximisation.

Table S2. CZP activity inhibition percentages determined in decreasing concentrations of Triton X-100, and in the absence of Triton X-100 but with a 10-min preincubation of the compounds with 4 mg/mL BSA.

Compd ID	Triton 0.1%	Triton 0.01%	Triton 0%	Triton 0% + BSA
**Es-15**	36 ± 7	41 ± 1	44 ± 5	40 ± 3
**Ald-6**	44 ± 4	31 ± 3	43 ± 5	37 ± 3
**Ald-10**	46 ± 8	43 ± 2	47 ± 6	43 ± 2

Table S3. Comparison of CZP activity inhibition percentages (%inh) with (w/inc.) and without (wo/inc.) prior incubation. Inhibitors were tested at 100μM. *E-64: positive control.

ID	% CZP inhib. w/inc.	% CZP inhib. wo/inc.
**Es-10**	47 $\pm$ 6	43 $\pm$ 4
**Es-15**	86 $\pm$ 1	81 $\pm$ 2
**Ald-6**	81 $\pm$ 2	18 $\pm$ 3
**Ald-10**	87 $\pm$ 1	21 $\pm$ 1
**E-64***	98 $\pm$ 2	12 $\pm$ 8

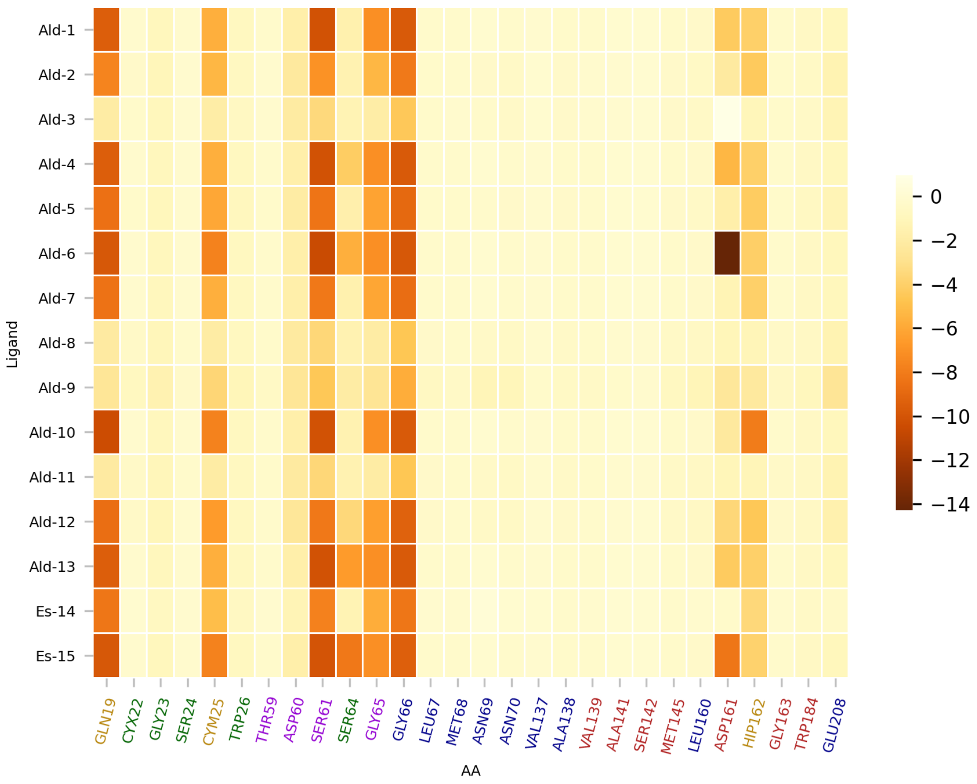

Figure S13. MMPBSA per-residue energetic analysis of the **electrostatic** interactions between **Ald-1** to **Ald-13** and **Es-14** to **Es-15** with CZP; highlighting the AA constituting the active site of CZP with different colours according to whether they belong to the **S1**, **S2**, **S3** and **S1'** or are part of the **catalytic triad** and **oxyanion hole**.

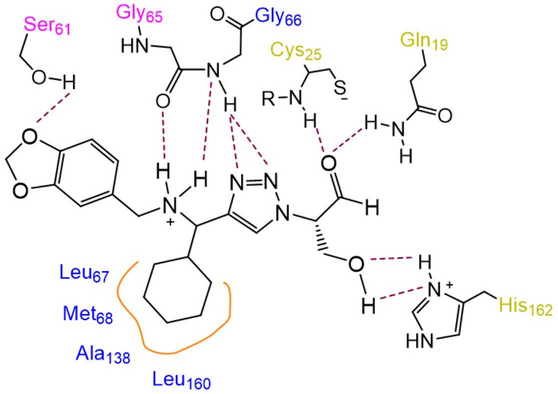

(a)

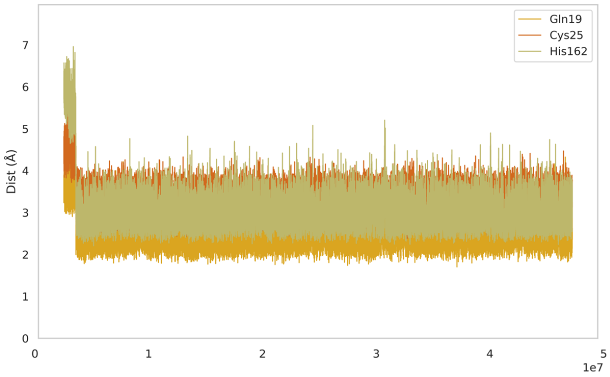

(b)

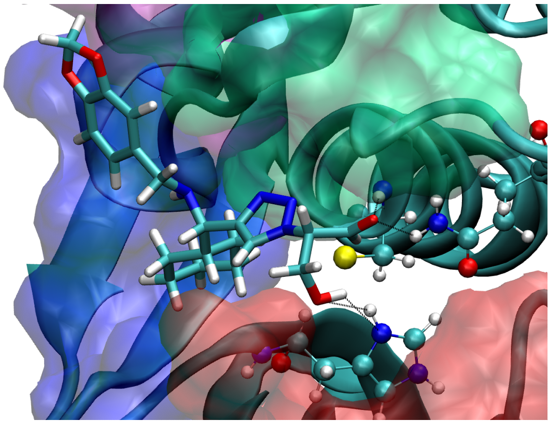

(c)

Figure S14. (a) Interaction pattern identified for **Ald-10**:**CZP**. (b) Distance plots of $\mathbf{R}^1$-hydroxymethyl of **Ald-10** with the backbone of, **Gln19**, **Cys25** and **His162**. (c) 3D-depiction of the **Ald-10**:**CZP** binding complex, focused on the interactions of **WH** and $\mathbf{R}^1$ with the **catalytic triad** and **oxyanion hole**.

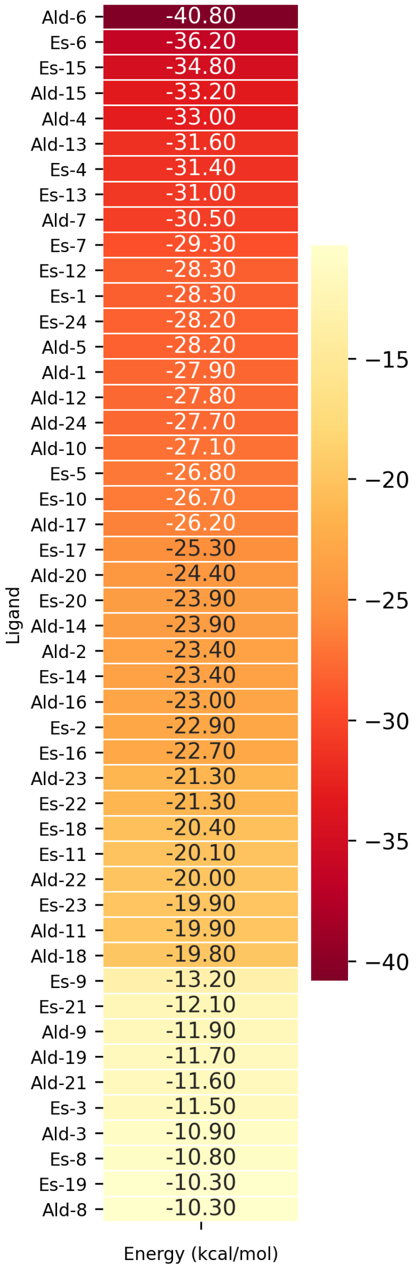

Figure S15. MMPBSA energetic analysis of the complex between CZP and each triazole-based compound synthesised and tested, ordered by energy. Values are reported in kcal.mol^{-1}.

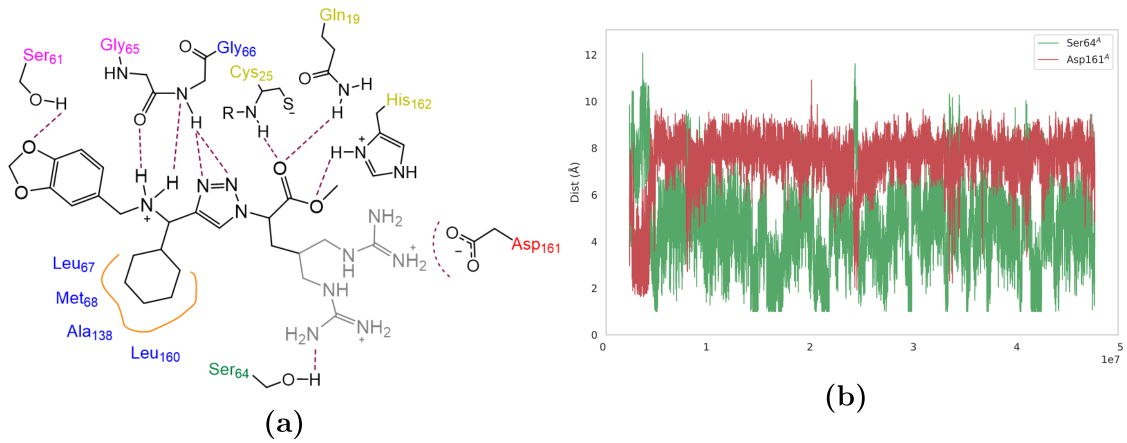

(a) (b)

Figure S16. (a) Interaction pattern identified for **Es-15**:**CZP**. The alternative poses of the $\mathbf{R}^1$ substituent are represented (gray). (b) Distance plots of $\mathbf{R}^1$-guanidinium of **Es-15** with **Ser64** and **Asp161**.

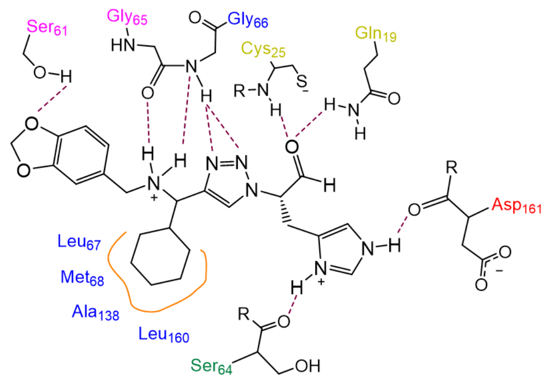

(a)

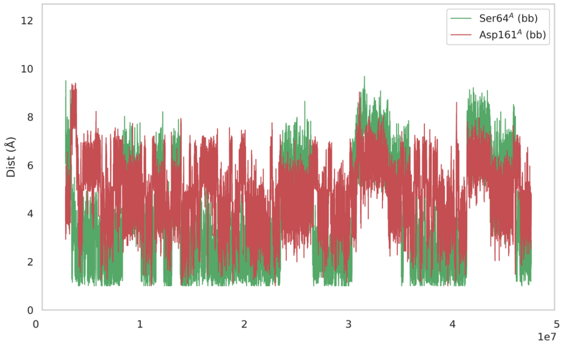

(b)

Figure S17. (a) Interaction pattern identified for **Ald-4**:**CZP**. (b) Distance plots of $\mathbf{R}^1$-methylimidazolium of **Ald-4** with the backbone (bb) of **Ser64** and **Asp161** of **CZP**.

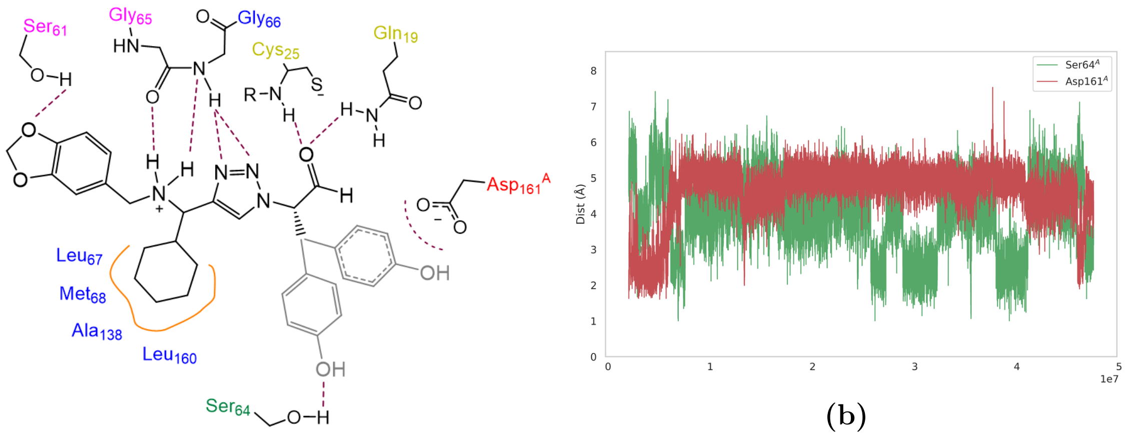

(a) (b)

Figure S18. (a) Interaction pattern identified for **Ald-13**:**CZP**. The alternative poses of the **R**[1] substituent are represented (gray). (b) Distance plots of **R**[1]-*p*-hydroxybenzyl of **Ald-13** with CZP-**Asp161** and **Ser64**.

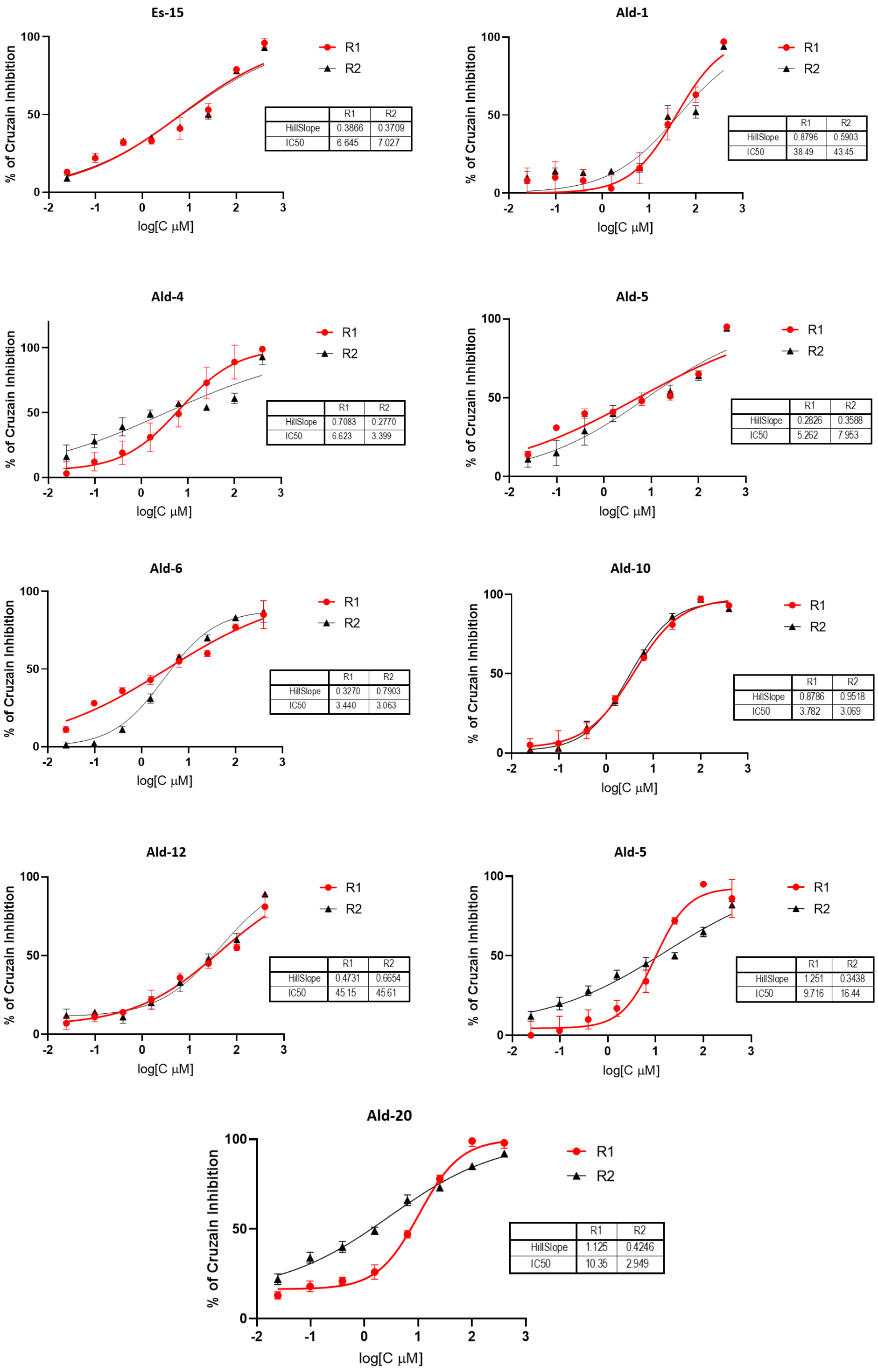

Figure S19. IC_{50} curves determined in two independent measurements performed in triplicate. Compounds in at least eight distinct concentrations were incubated with CZP for 10min, and once the substrate was added, the fluorescence was monitored for 10min at 5s intervals. IC50 curves were obtained from a non-linear fit of the inhibition values versus the logaritimic concentration of the compounds.

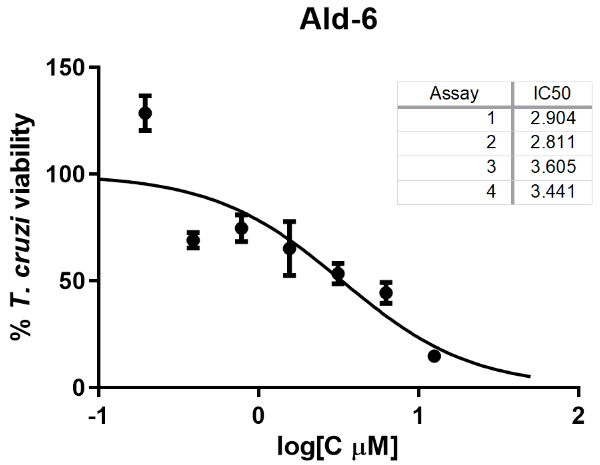

Figure S20. IC_{50} curve determined in four independent measurements performed in triplicate for Ald-6 inhibition of *T. cruzi*. IC50 curves were obtained from a non-linear fit of the inhibition values versus the logaritimic concentration of the compounds.

Synthesis details, characterization, and 1H NMR and 13C NMR spectra for all precursors and triazole-derivatives described and evaluated.

Ethyl 2-azido-3-phenylpropanoate (**Az-1**). Prepared according to General procedure **A**, starting from L-phenylalanine ethyl ester hydrochloride (3 mmol, 689.1 mg) to afford a yellow oil in 95% yield (625.5 mg). ^{1}H NMR (300 MHz, $CDCl_3$) δ (ppm): 7.23-7.40 (m, 5H), 5.40-5.43 (t, 1H), 4.07-4.20 (q, 2H), 3.19-3.31 (d, 2H), 1.13-1.15 (t, 3H). FTIR (cm^{-1}): 2129 (N_3), 1207 (C=O).

Methyl 2-azido-3-methylbutanoate (**Az-2**). Prepared according to General procedure **A**, starting from L-valine methyl ester hydrochloride (1 mmol, 168 mg) to afford a clear oil with yellowish hue in 89% yield (140.1 mg). ^{1}H NMR (400 MHz, $CDCl_3$) δ (ppm): 5.08-5.10 (d, 1H), 3.63 (s, 3H), 2.26-2.28 (m, 1H), 0.89-0.96 (dd, 6H). FTIR (cm^{-1}): 2109 (N_3), 1238 (C=O).

4-Azido-5-methoxy-5-oxopentanoic acid (**Az-3**). Prepared according to General procedure **A**, starting from L-glutamic acid 1-methyl ester (1.5 mmol, 241.7 mg) to afford a white solid in 16% yield (45.5 mg). ^{1}H NMR (300 MHz, $CDCl_3$) δ (ppm): 5.38-5.40 (t, 1H), 3.91 (s, 3H), 2.32-2.44 (t, 2H), 2.10-2.18 (m, 2H). FTIR (cm^{-1}): 2139 (N_3), 1716-1722 (C=O).

Methyl 2-azido-3-(1H-imidazol-4-yl)propanoate (**Az-4**). Prepared according to General procedure **A**, starting from L-histidine methyl ester dihydrochloride (2 mmol, 504.2 mg) to afford a yellow solid in 40% yield (150.8 mg). ^{1}H NMR (400 MHz, $CDCl_3$) δ (ppm): 7.35-7.37 (d, 1H), 6.88-6.89 (d, 1H), 5.45-5.47 (t, 1H), 3.89 (s, 3H), 3.47-3.62 (dd, 2H). FTIR (cm^{-1}): 2147 (N_3), 1751 (C=O).

Methyl 2-azido-3-methylpentanoate (**Az-5**). Prepared according to General procedure **A**, starting from L-leucine methyl ester hydrochloride (1 mmol, 182 mg) to afford a clear oil with yellowish hue in 91% yield (155.8 mg). ^{1}H NMR (300 MHz, $CDCl_3$) δ (ppm): 5.16-5.18 (t, 1H), 3.61 (s, 3H), 1.81-1.96 (d, 2H), 1.66-1.69 (m, 1H), 0.87-1.01 (d, 6H). FTIR (cm^{-1}):

2111 (N_3), 1732 (C=O).

Methyl 2-azido-6-((tert-butoxycarbonyl)amino)hexanoate (**Az-6**). Prepared according to General procedure **A**, starting from $N\epsilon$-Boc-L-lysine methyl ester hydrochloride (1.7 mmol, 504.5 mg) to afford a white solid in 35% yield (173.3 mg). ^{1}H NMR (400 MHz, $CDCl_3$) δ 5.26-5.29 (t, 1H), 3.76 (s, 3H), 3.23-3.37 (t, 2H), 1.86-1.97 (m, 2H), 1.63-1.71 (m, 2H), 1.36-1.43 (m, 11H). FTIR (cm^{-1}): 2104 (N_3), 1742-1747 (C=O), 1694 (N-H).

Ethyl 2-azido-4-(methylthio)butanoate (**Az-7**). Prepared according to General procedure **A**, starting from L-methionine ethyl ester hydrochloride (1 mmol, 213.7 mg) to afford a yellow oil in 60% yield (122.2 mg). ^{1}H NMR (300 MHz, $CDCl_3$) δ (ppm): 5.33-5.36 (t, 1H), 4.06-4.19 (m, 2H), 2.74-2.87 (t, 2H), 2.23 (s, 3H), 1.73-1.89 (m, 2H), 1.04-1.06 (t, 3H). FTIR (cm^{-1}): 2115 (N_3), 1194 (C=O).

Methyl 2-azidopropanoate (**Az-8**). Prepared according to General procedure **A**, starting from L-alanine methyl ester hydrochloride (0.8 mmol, 112 mg) to afford a clear oil in 86% yield (89.0 mg). ^{1}H NMR (400 MHz, $CDCl_3$) δ (ppm): 5.43-5.45 (q, 1H), 3.81 (s, 3H), 1.59-1.60 (d, 3H). FTIR (cm^{-1}): 2119 (N_3), 1691 (C=O).

Methyl 2-azido-2-phenylacetate (**Az-9**). Prepared according to General procedure **A**, starting from L-phenylglycine methyl ester hydrochloride (3 mmol, 605.0 mg) to afford a yellow oil in 89% yield (509.3 mg). ^{1}H NMR (400 MHz, $CDCl_3$) δ (ppm): 7.31-7.52 (m, 5H), 6.84 (s, 1H), 3.57 (s, 3H). FTIR (cm^{-1}): 2121 (N_3), 1219 (C=O).

Methyl 2-azido-3-hydroxypropanoate (**Az-10**). Prepared according to General procedure **A**, starting from L-serine methyl ester hydrochloride (0.6 mmol, 93 mg) to afford a clear oil with yellowish hue in 75% yield (65.3 mg). ^{1}H NMR (400 MHz, $CDCl_3$) δ (ppm): 5.43-5.44 (t, 1H), 3.64 (s, 3H), 3.29-3.40 (d, 2H). FTIR (cm^{-1}): 2144 (N_3), 1241 (C=O), 1074 (O-H).

Methyl 2-azido-3-hydroxybutanoate (**Az-11**). Prepared according to General procedure **A**, starting from L-threonine methyl ester hydrochloride (0.5 mmol, 85 mg) to afford a clear oil with yellowish hue in 73% yield (58.0 mg). ^{1}H NMR (300 MHz, $CDCl_3$) δ (ppm): 5.22-5.24 (d, 1H), 3.85-3.86 (m, 1H), 3.59 (s, 3H), 2.11-2.27 (d, 3H). FTIR (cm^{-1}): 2144 (N_3),

1241 (C=O), 1074 (O-H).

Methyl 2-azido-3-(1H-indol-3-yl)propanoate (**Az-12**). Prepared according to General procedure **A**, starting from L-tryptophan methyl ester hydrochloride (2 mmol, 509.4 mg) to afford a yellow oil in 66% yield (321.4 mg). ^{1}H NMR (400 MHz, $CDCl_3$) δ (ppm): 7.43-7.46 (m, 1H), 7.14-7.27 (m, 2H), 6.99-7.14 (m, 2H), 5.72-5.73 (t, 1H), 3.71 (s, 3H), 3.27-3.39 (d, 2H). FTIR (cm^{-1}): 2133 (N_3), 1218 (C=O).

Methyl 2-azido-3-(4-hydroxyphenyl)propanoate (**Az-13**). Prepared according to General procedure **A**, starting from L-tyrosine methyl ester hydrochloride (1 mmol, 231.7 mg) to afford a yellow oil in 73% yield (161.0 mg). ^{1}H NMR (400 MHz, $CDCl_3$) δ (ppm): 7.07-7.09 (d, 2H), 6.78-6.80 (d, 2H), 4.98 (s, 1H), 4.01-4.03 (dd, 1H), 3.77 (s, 3H), 3.08-3.10 (dd, 1H), 2.95-2.96 (dd, 1H). FTIR (cm^{-1}): 2098 (N_3), 1223 (C=O).

Methyl 2-azido-3-mercaptopropanoate (**Az-14**). Prepared according to General procedure **A**, starting from L-cysteine methyl ester hydrochloride (2 mmol, 343.3 mg) to afford a yellow oil in 90% yield (290.1 mg). ^{1}H NMR (400 MHz, $CDCl_3$) δ (ppm): 5.24-5.26 (t, 1H), 3.12 (s, 3H), 2.87-2.94 (dd, 2H), 1.43 (s, 1H). FTIR (cm^{-1}): 2135 (N_3), 2557 (SH).

Methyl 2-azido-5-guanidinopentanoate (**Az-15**). Prepared according to General procedure **A**, starting from L-arginine methyl ester dihydrochloride (2 mmol, 522.3 mg). After concentrating the crude under reduced pressure, the residue was resolved in a 50mL CH_3OH:H_2O (v/v=1:1) mixture[2] to afford an orange solid in 37% yield (156.8 mg). ^{1}H NMR (400 MHz, d_6–DMSO) δ (ppm): 7.96-8.15 (m, 4H), 5.66-5.68 (m, 1H), 3.83 (s, 3H), 3.08-3.24 (m, 2H), 2.05-2.22 (m, 2H), 1.59-1.81 (m, 2H). FTIR (cm^{-1}): 2145 (N_3), 1705 (C=O).

N-(benzo[d][1,3]dioxol-5-ylmethyl)-1-cyclohexylprop-2-yn-1-amine (**PA-1**). To a flame-dried 10 mL Pyrex pressure vessel equipped with a magnetic stirring bar, copper bromide (0.4 mmol, 57.4 mg) was added. While flushing with argon and stirring, dry toluene (4.0 mL) followed by the cyclohexanecarboxaldehyde (2 mmol, 224.3 mg), piperonylamine (2.6

mmol, 393.0 mg) and trimethylsilylacetylene (3.2 mmol, 314.2 mg) added. After flushing for another 2 minutes, the reaction vessel was sealed with a PTFE-Silicon septum and irradiated in a CEM-Discover microwave reactor at the stipulated reaction conditions (25 min, ceiling temperature of 125°C, maximum power of 300 W). After cooling down to room temperature, the organic phase was removed under reduced pressure and the crude was loaded onto a silica gel column to afford the corresponding silylated product. Column chromatography was carried out using a petroleum ether/EtOAc gradient (9:1 – 4:1) to afford a yellowish oil in 89% yield (615.6 mg). A solution of the product in methanol (1 M) at 0°C was prepared to which potassium hydroxide (1eq) was added. The mixture was stirred at room temperature until full conversion, quenched with water and extracted three times with EtOAc. After removal of the solvent under reduced pressure, **PA-1** was obtained as a yellowish oil in quantitative yield (481.5 mg). ^{1}H NMR (300 MHz, $CDCl_3$) δ (ppm): 6.80-6.88 (m, 3H), 5.93 (s, 2H), 3.70-3.93 (dd, 2H), 3.14-3.15 (d, 1H), 2.30 (s, 1H), 1.22-1.80 (m, 11H).

2-(1-Cyclohexylprop-2-yn-1-yl)-1,2,3,4-tetrahydroisoquinoline (**PA-2**). This method was adapted from the one reported by Ermolat'ev et al.[3] To a flame-dried 10 mL Pyrex pressure vessel equipped with a magnetic stirring bar, copper iodide (0.2 mmol, 38 mg) was added. While flushing with argon and stirring, dry toluene (1.0 mL) followed by cyclohexanecarboxaldehyde (1 mmol, 112.5 mg), 1,2,3,4-tetrahydroisoquinoline (1.2 mmol, 160 mg) and propiolic acid (1.5 mmol, 105 mg) were added. After flushing for another 2 minutes, the reaction vessel was sealed with a PTFE-Silicon septum and irradiated in a CEM-Discover microwave reactor at the stipulated reaction conditions (15 min, ceiling temperature of 100°C, maximum power of 100 W). After cooling down to room temperature, the organic phase was removed under reduced pressure and the crude was loaded onto a silica gel column to afford the corresponding product. Column chromatography was carried out using a petroleum ether/EtOAc gradient (9:1 – 4:1) to afford **PA-2** as yellow solid in 71% yield (180.7 mg). ^{1}H NMR (400 MHz, $CDCl_3$) δ (ppm): 6.91-7.17 (m, 4H), 3.69-3.71 (t, 1H), 3.41-3.59 (dd, 2H), 3.02 (s, 1H), 2.65-2.87 (m, 4H), 2.37-2.41 (m, 1H), 1.07-1.23 (m, 10H).

Ethyl 2-(4-(((benzo[d][1,3]dioxol-5-ylmethyl)amino)(cyclohexyl)methyl)-1 H-1,2,3-triazol-1-yl)-3-phenylpropanoate (**Es-1**). Prepared according to General procedure **B**, using **Az-1** (0.3 mmol, 65.8.2 mg) and **PA-1** (0.3 mmol, 81.4 mg). 6h. Flash chromatography was carried out using a petroleum ether/EtOAc mixture as the eluent (1:1) to afford a yellow oil in 89% yield (131.2 mg). ^{1}H NMR (300 MHz, $CDCl_3$) δ (ppm): 7.37 (s, 1H), 7.12-7.27 (m, 5H), 6.76 (s, 1H), 6.62-6.68 (d, 2H), 5.91 (s, 2H), 5.79-5.82 (m, 1H), 4.13-4.19 (q, 2H), 3.83-3.84 (d, 1H), 3.37-3.66 (dd, 4H), 1.84-1.87 (m, 13H), 0.82-1.22 (m, 13H). ^{13}C NMR δ (ppm): 168.38, 150.66, 148.06, 145.02, 138.85, 134.47, 128.07 (2C), 127.91 (2C), 127.62, 127.40, 122.27, 108.78, 107.59, 100.54, 63.92, 59.37, 56.87, 51.44, 43.81, 37.64, 30.01, 29.90, 26.38, 26.09, 25.93, 14.57.

Methyl 2-(4-(((benzo[d][1,3]dioxol-5-ylmethyl)amino)(cyclohexyl)methyl)-1H-1,2,3-triazol-1-yl)-3-methylbutanoate (**Es-2**). Prepared according to General procedure **B**, using **Az-2** (0.2 mmol, 31.4 mg) and **PA-1** (0.2 mmol, 54.3 mg). 7h. A yellow oil was afforded in 85% yield (73.2 mg). ^{1}H NMR (300 MHz, $CDCl_3$) δ (ppm): 7.07 (s, 1H), 6.69 (s, 1H), 6.54-6.60 (d, 2H), 5.74 (s, 2H), 5.41-5.46 (d, 1H), 3.50-3.75 (dd, 2H), 3.21 (s, 3H), 2.94-2.96 (d, 1H), 1.47-2.15 (m, 12H), 0.96-1.05 (d, 6H). ^{13}C NMR δ (ppm): 169.23, 147.78, 146.78, 139.71, 134.28, 120.02, 119.83, 108.64, 108.60, 101.57, 59.33, 55.55, 53.80, 51.46, 42.76, 30.47, 29.61, 29.56, 26.68, 26.57, 25.04, 18.94, 18.85.

4-(4-(((Benzo[d][1,3]dioxol-5-ylmethyl)amino)(cyclohexyl)methyl)-1H-1,2,3-triazol-1-yl)-5-methoxy-5-oxopentanoic acid (**Es-3**). Prepared according to General procedure **B**, using **Az-3** (0.2 mmol, 37.4 mg) and **PA-1** (0.2 mmol, 54.3 mg). 6h. Flash chromatography was carried out using EtOAc mixture as the eluent to afford a yellow solid in 50% yield (45.9 mg). ^{1}H NMR (300 MHz, $CDCl_3$) δ (ppm): 7.34 (s, 1H), 6.88 (s, 1H), 6.74-6.82 (m, 2H), 5.93 (s, 2H), 5.53-5.56 (t, 1H), 3.69-3.96 (dd, 2H), 3.20 (s, 3H), 2.89-2.89 (d, 1H), 2.51-2.54 (t, 2H), 1.90-1.93 (t, 2H), 1.41-1.73 (m, 11H). ^{13}C NMR δ (ppm): 179.13, 166.60, 147.55, 147.05, 132.38, 131.66, 127.63, 124.66, 108.66, 108.09, 101.18, 57.03, 56.93, 52.58, 52.42,

40.68, 29.96, 29.48, 29.03, 28.23, 25.46 (2C), 24.91.

Methyl 2-(4-(((benzo[d][1,3]dioxol-5-ylmethyl)amino)(cyclohexyl)methyl)-1H-1,2,3-triazol-1-yl)-3-(1H-imidazol-4-yl)propanoate (**Es-4**). Prepared according to General procedure **B**, using **Az-4** (0.2 mmol, 39.0 mg) and **PA-1** (0.2 mmol, 54.3 mg). 6h. Flash chromatography was carried out using EtOAc as the eluent to afford an orangewish oil in 61% yield (56.7 mg). ^{1}H NMR (300 MHz, $CDCl_3$) δ (ppm): 7.56-7.60 (d, 1H), 7.41 (s, 1H), 6.88 (s, 1H), 6.80-6.82 (d, 2H), 6.74-6.76 (d, 1H), 5.90 (s, 2H), 5.80-5.87 (t, 1H), 4.10-4.11 (d, 1H), 3.80 (s, 3H), 3.64-3.62 (d, 2H), 2.97-3.23 (dd, 2H), 0.88-1.91 (m, 11H). ^{13}C NMR δ (ppm): 173.36, 147.68, 147.38, 135.92, 132.14, 130.50, 128.67, 128.51, 122.87, 109.77, 108.02, 106.30, 100.49, 57.03, 56.94, 52.55, 46.18, 39.18, 28.19, 28.08, 26.44, 26.36, 24.72, 24.05.

Methyl 2-(4-(((benzo[d][1,3]dioxol-5-ylmethyl)amino)(cyclohexyl)methyl)-1H-1,2,3-triazol-1-yl)-4-methylpentanoate (**Es-5**). Prepared according to General procedure **B**, using **Az-5** (0.2 mmol, 34.2 mg) and **PA-1** (0.2 mmol, 54.3 mg). 12h. A yellow oil was afforded in 90% yield (79.6 mg). ^{1}H NMR (300 MHz, $CDCl_3$) δ (ppm): 7.41 (s, 1H), 6.68 (s, 1H), 6.53-6.62 (d, 2H), 5.74 (s, 2H), 5.31-5.34 (t, 1H), 3.89 (s, 3H), 3.79-3.81 (d, 1H), 3.14-3.43 (dd, 2H), 2.05-2.13 (d, 2H), 1.26-2.00 (m, 12H), 1.03-1.05 (d, 6H). ^{13}C NMR δ (ppm): 170.47, 147.31, 147.00, 136.24, 133.88, 128.53, 123.02, 108.44 (2C), 101.66, 57.04, 56.86, 51.68, 51.01, 39.15, 35.66, 29.73, 29.36, 26.45, 26.19, 26.10, 24.55, 22.09 (2C).

Methyl 6-amino-2-(4-(((benzo[d][1,3]dioxol-5-ylmethyl)amino)(cyclohexyl) methyl)-1H-1,2,3-triazol-1-yl)hexanoate (**Es-6**). Prepared according to General procedure **B**, using **Az-6** (0.2 mmol, 57.3 mg) and **PA-1** (0.2 mmol, 54.3 mg). 8h. Flash chromatography was carried out using EtOAc as the eluent to afford the N-Boc product as a yellow solid in 48% yield (53.8 mg). It was added to a solution of DCM/TFA (1:1 v/v, 0.5 mL) at 0°C and stirred 20 min. The mixture was concentrated *in vacuo* to afford the product as a pale yellow solid without further purification (43.8 mg). ^{1}H NMR (400 MHz, $CDCl_3$) δ (ppm): 7.34 (s, 1H), 6.84 (s, 1H), 6.72-6.75 (d, 2H), 5.90 (s, 2H), 5.03-4.99 (t, 1H), 3.92 (s, 3H), 3.27-3.31 (t, 2H), 2.96-3.04 (m, 3H), 1.14-2.04 (m, 15H), 0.86-0.95 (m, 2H). ^{13}C NMR δ (ppm): 171.46,

147.21, 146.32, 133.98, 129.94, 129.07, 125.34, 108.66, 108.17, 101.06, 54.88, 54.54, 52.18, 51.48, 40.98, 37.12, 30.48, 29.66, 29.34, 28.77, 26.41, 25.97, 25.78, 22.98.

Ethyl 2-(4-(((benzo[d][1,3]dioxol-5-ylmethyl)amino)(cyclohexyl)methyl)-1 H-1,2,3-triazol-1-yl)-4-(methylthio)butanoate (**Es-7**). Prepared according to General procedure **B**, using **Az-7** (0.2 mmol, 40.7 mg) and **PA-1** (0.2 mmol, 54.3 mg). 6h. Flash chromatography was carried out using a petroleum ether/EtOAc mixture as the eluent (1:1) to afford a yellow oil in 84% yield (80.0 mg). ^{1}H NMR (300 MHz, $CDCl_3$) δ (ppm): 7.55 (s, 1H), 6.87 (s, 1H), 6.74-6.82 (m, 2H), 5.93 (s, 2H), 4.66-4.70 (m, 3H), 3.70-3.94 (dd, 2H), 3.30-3.35 (m, 1H), 2.55-2.57 (t, 2H), 2.31 (s, 3H), 1.30-1.67 (m, 13H), 0.86-0.92 (t, 3H). ^{13}C NMR δ (ppm): 167.87, 149.68, 147.05, 146.13, 134.22, 127.65, 121.11, 108.64, 108.01, 101.13, 61.36, 59.44, 56.71, 51.58, 43.17, 31.38, 29.86, 29.47, 29.41, 26.54, 26.31, 26.11, 16.84, 13.89.

Methyl 2-(4-(((benzo[d][1,3]dioxol-5-ylmethyl)amino)(cyclohexyl)methyl)-1H-1,2,3-triazol-1-yl)propanoate (**Es-8**). Prepared according to General procedure **B**, using **Az-8** (0.2 mmol, 25.8 mg) and **PA-1** (0.2 mmol, 54.3 mg). 6h. A yellow oil was afforded in 84% yield (67.1 mg). ^{1}H NMR (300 MHz, $CDCl_3$) δ (ppm): 7.35 (s, 1H), 6.77 (s, 1H), 6.64-6.70 (m, 2H), 5.89 (s, 2H), 5.43-5.49 (q, 1H), 4.06 (s, 3H), 3.59-3.62 (d, 1H), 3.37-3.56 (dd, 2H), 0.56-1.96 (m, 14H). ^{13}C NMR δ (ppm): 171.12, 146.33, 146.02, 134.12, 129.31, 124.84, 121.20, 108.43, 108.01, 101.02, 57.10, 56.87, 56.61, 52.33, 46.18, 29.82, 29.44, 26.50, 26.22, 26.16, 20.89.

Methyl 2-(4-(((benzo[d][1,3]dioxol-5-ylmethyl)amino)(cyclohexyl)methyl)-1H-1,2,3-triazol-1-yl)-2-phenylacetate (**Es-9**). Prepared according to General procedure **B**, using **Az-9** (0.2 mmol, 38.2 mg) and **PA-8** (0.2 mmol, 54.3 mg). 10h. Flash chromatography was carried out using a petroleum ether/EtOAc mixture as the eluent (1:1) to afford a yellow oil in 82% yield (75.8 mg). ^{1}H NMR (300 MHz, $CDCl_3$) δ (ppm): 7.36-7.49 (m, 5H), 7.47 (s, 1H), 6.74-6.77 (dd, 1H), 6.60-6.71 (m, 2H), 6.59-6.60 (d, 1H), 5.92 (s, 2H), 3.85 (s, 3H), 3.51-3.54 (d, 1H), 3.36-3.62 (dd, 2H), 1.87-1.92 (m, 1H), 0.83-1.51 (m, 10H). ^{13}C NMR δ (ppm): 169.27, 148.51, 147.73, 139.12, 134.50, 129.90, 128.01 (2C), 127.96, 127.60, 127.11 (2C), 121.34, 108.09, 107.91, 100.83, 57.66, 57.21, 52.08, 49.92, 39.97, 29.27, 28.33, 25.48,

24.36, 23.99.

Methyl 2-(4-(((benzo[d][1,3]dioxol-5-ylmethyl)amino)(cyclohexyl)methyl)-1H-1,2,3-triazol-1-yl)-3-hydroxypropanoate (**Es-10**). Prepared according to General procedure **B**, using **Az-10** (0.2 mmol, 29.0 mg) and **PA-1** (0.2 mmol, 54.3 mg). 8h. Flash chromatography was carried out using a petroleum ether/EtOAc mixture as the eluent (1:1) to afford a yellow oil in 87% yield (72.5 mg). ^{1}H NMR (300 MHz, $CDCl_3$) δ (ppm): 7.91 (s, 1H), 6.82 (s, 1H), 6.69-6.82 (d, 2H), 5.92 (s, 2H), 5.19-5.26 (t, 1H), 4.13-4.16 (d, 2H), 4.09 (s, 3H), 3.77-3.79 (d, 1H), 3.51-3.77 (dd, 2H), 0.86-1.96 (m, 11H). ^{13}C NMR δ (ppm): 162.43, 150.74, 147.79, 146.66, 134.44, 128.71, 121.08, 108.83, 108.18, 101.04, 66.43, 59.42, 56.63, 52.48, 51.56, 43.01, 29.83, 29.40, 26.71, 26.29, 26.17.

Methyl 2-(4-(((benzo[d][1,3]dioxol-5-ylmethyl)amino)(cyclohexyl)methyl)-1H-1,2,3-triazol-1-yl)-3-hydroxybutanoate (**Es-11**). Prepared according to General procedure **B**, using **Az-11** (0.2 mmol, 32.0 mg) and **PA-1** (0.2 mmol, 54.3 mg). 8h. Flash chromatography was carried out using a petroleum ether/EtOAc mixture as the eluent (1:1) to afford a yellow oil in 65% yield (56.2 mg). ^{1}H NMR (300 MHz, $CDCl_3$) δ (ppm): 7.94 (s, 1H), 6.83 (s, 1H), 6.72-6.73 (d, 2H), 5.92 (s, 2H), 4.32-4.33 (d, 1H), 4.29-4.31 (m, 1H), 3.80-3.86 (d, 1H), 3.54-3.78 (dd, 2H), 3.13 (s, 3H), 1.68-2.12 (m, 11H), 1.09-1.17 (d, 3H). ^{13}C NMR δ (ppm): 168.10, 150.20, 147.48, 146.24, 134.42, 127.22, 121.45, 121.03, 108.57, 107.86, 100.68, 68.97, 58.17, 57.05, 52.43, 51.39, 42.47, 32.53, 31.41, 28.20, 26.80, 25.75, 16.84.

Methyl 2-(4-(((benzo[d][1,3]dioxol-5-ylmethyl)amino)(cyclohexyl)methyl)-1H-1,2,3-triazol-1-yl)-3-(1H-indol-3-yl)propanoate (**Es-12**). Prepared according to General procedure **B**, using **Az-12** (0.2 mmol, 48.9 mg) and **PA-1** (0.2 mmol, 54.3 mg). 7h. Flash chromatography was carried out using a petroleum ether/EtOAc mixture as the eluent (1:1) to afford a yellow oil in 68% yield (69.8 mg). ^{1}H NMR (400 MHz, $CDCl_3$) δ (ppm): 7.60 (s, 1H), 7.37-7.40 (m, 1H), 7.13-7.24 (m, 4H), 6.66 (s, 1H), 6.56-6.59 (d, 2H), 5.92 (s, 2H), 4.17-4.20 (t, 1H), 4.11-4.13 (d, 1H), 3.76 (s, 3H), 3.66-3.68 (d, 2H), 3.18-3.40 (ddd, 2H), 0.79-1.67 (m, 11H). ^{13}C NMR δ (ppm): 169.66, 148.39, 145.67, 144.42, 138.87, 134.21, 128.89, 128.62, 128.00,

126.86, 123.86, 122.26, 121.21, 112.38, 108.24, 108.04, 101.69, 58.31, 57.89, 53.39, 51.07, 45.59, 30.10, 29.48, 28.36, 25.34, 25.15, 23.74.

Methyl 2-(4-(((benzo[d][1,3]dioxol-5-ylmethyl)amino)(cyclohexyl)methyl)-1H-1,2,3-triazol-1-yl)-3-(4-hydroxyphenyl)propanoate (**Es-13**). Prepared according to General procedure **B**, using **Az-13** (0.2 mmol, 44.2 mg) and **PA-1** (0.2 mmol, 54.3 mg). 8h. Flash chromatography was carried out using a petroleum ether/EtOAc mixture as the eluent (1:1) to afford a yellow oil in 51% yield (49.8 mg). ^{1}H NMR (300 MHz, $CDCl_3$) δ (ppm): 8.08-8.07 (d, 2H), 7.57 (s, 1H), 6.81 (s, 1H), 6.71-6.72 (d, 2H), 6.08-6.14 (d, 2H), 5.93 (s, 2H), 5.44-5.49 (m, 1H), 3.78 (s, 3H), 3.66-3.67 (d, 1H), 3.59-3.65 (dd, 2H), 2.17-2.20 (d, 2H), 1.05-1.74 (m, 11H). ^{13}C NMR δ (ppm): 169.64, 153.10, 146.81, 146.11, 135.15, 134.88, 134.26, 133.14, 130.12, 127.48, 121.11, 120.22, 119.10, 108.85, 106.54, 101.05, 58.37, 55.44, 52.98, 50.66, 42.09, 39.77, 31.92, 30.80, 27.78, 27.58, 26.18.

Methyl 2-(4-(((benzo[d][1,3]dioxol-5-ylmethyl)amino)(cyclohexyl)methyl)-1H-1,2,3-triazol-1-yl)-3-mercaptopropanoate (**Es-14**). Prepared according to General procedure **B**, using **Az-14** (0.3 mmol, 48.4 mg) and **PA-1** (0.3 mmol, 81.4 mg). 6h. Flash chromatography was carried out using EtOAc as the eluent to afford a yellow oil in 73% yield (95.3 mg). ^{1}H NMR (400 MHz, $CDCl_3$) δ (ppm): 7.39 (s, 1H), 6.74-6.88 (m, 3H), 5.93 (s, 2H), 5.32-5.34 (t, 1H), 4.00-4.26 (dd, 2H), 3.70 (s, 3H), 3.18-3.22 (dd, 1H), 2.52-2.72 (dd, 2H), 0.72-1.85 (m, 12H). ^{13}C NMR δ (ppm): 171.71, 146.83, 145.61, 130.47, 128.38, 125.44, 123.69, 107.20, 106.68, 100.02, 56.00, 55.25, 53.16, 50.36, 40.07, 32.06, 31.91, 26.87, 25.89, 25.10 (2C).

Methyl 2-(4-(((benzo[d][1,3]dioxol-5-ylmethyl)amino)(cyclohexyl)methyl)-1H-1,2,3-triazol-1-yl)-5-guanidinopentanoate (**Es-15**). Prepared according to General procedure **B**, using **Az-15** (0.2 mmol, 43.0 mg) and **PA-1** (0.2 mmol, 54.3 mg). 48h. Flash chromatography was carried out using methanol as the eluent to afford a yellow solid in 43% yield (41.3 mg). ^{1}H NMR (400 MHz, d_6-DMSO) δ (ppm): 9.08 (s, 1H), 8.02-8.14 (m, 4H), 7.06 (s, 1H), 6.77-6.96 (m, 3H), 6.18-6.19 (d, 1H), 5.99 (s, 2H), 4.07-4.11 (d, 1H), 3.78-3.81 (dd, 2H), 3.49 (s, 3H), 2.58-2.74 (m, 2H), 2.18-2.30 (m, 2H), 1.78-1.92 (m, 2H), 1.19-1.64 (m, 11H). ^{13}C

NMR δ (ppm): 176.04, 159.56, 148.32, 146.55, 136.67, 134.54, 129.83, 126.78, 108.80, 108.52, 104.83, 58.24, 58.09, 52.42, 43.89, 39.18, 35.80, 35.21, 32.89, 29.98, 29.30, 25.74, 25.49, 25.37. HRMS: m/z $[M+H]^+$ calcd. for $C_{24}H_{35}N_7O_4$: 485.2750; found: 486.3497.

Ethyl 2-(4-(cyclohexyl(3,4-dihydroisoquinolin-2(1H)-yl)methyl)-1H-1,2,3-triazol-1-yl)-3-phenylpropanoate (**Es-16**). Prepared according to General procedure **B**, using **Az-1** (0.5 mmol, 109.6 mg) and **PA-2** (0.5 mmol, 126.7 mg). 4h. Column chromatography was carried out using a petroleum ether/EtOAc gradient (9:1 – 1:1) to afford a yellow solid in 76% yield (179.4 mg). ^{1}H NMR (300 MHz, $CDCl_3$) δ (ppm): 7.94-8.11 (m, 5H), 7.91 (s, 1H), 7.00-7.10 (m, 4H), 5.36-3.38 (t, 1H), 4.01-4.22 (dd, 2H), 3.54-3.61 (m, 5H), 1.18-2.41 (m, 17H). ^{13}C NMR δ (ppm): 173.12, 137.03, 136.40, 135.47, 133.82, 130.32 (2C), 129.46 (4C), 126.76, 126.36, 124.07, 120.06, 68.08, 64.08, 56.02, 53.65, 47.59, 36.36, 33.37, 31.44, 28.68, 28.59, 28.40, 27.95, 25.30, 14.82.

Methyl 2-(4-(cyclohexyl(3,4-dihydroisoquinolin-2(1H)-yl)methyl)-1H-1,2,3-triazol-1-yl)-3-(1H-imidazol-4-yl)propanoate (**Es-17**). Prepared according to General procedure **B**, using **Az-4** (0.2 mmol, 39.0 mg) and **PA-2** (0.2 mmol, 50.7 mg). 6h. Flash chromatography was carried out using EtOAc as the eluent to afford an orange oil in 54% yield (48.4 mg). ^{1}H NMR (400 MHz, $CDCl_3$) δ (ppm): 7.44-7.45 (d, 1H), 6.99-7.13 (m, 5H), 4.54-5.57 (t, 1H), 3.92 (s, 3H), 3.77-3.85 (t, 1H), 3.64-3.82 (dd, 2H), 3.27-3.30 (dd, 2H), 2.85-2.89 (m, 4H), 2.58-2.66 (m, 1H), 1.11-1.26 (m, 10H). ^{13}C NMR δ (ppm): 176.25, 137.31, 135.53, 134.32, 132.65, 132.39, 129.63, 129.35, 128.89, 125.66, 120.43, 115.77, 67.08, 59.02, 56.65, 50.74, 50.59, 37.59, 31.40, 30.95, 28.30, 27.71, 25.70, 25.58, 25.39.

Ethyl 2-(4-(cyclohexyl(3,4-dihydroisoquinolin-2(1H)-yl)methyl)-1H-1,2,3-triazol-1-yl)-4-(methylthio)butanoate (**Es-18**). Prepared according to General procedure **B**, using **Az-7** (0.2 mmol, 40.7 mg) and **PA-2** (0.2 mmol, 50.7 mg). 6h. Flash chromatography was carried out using a petroleum ether/EtOAc mixture as the eluent (1:1) to afford a yellow oil in 60% yield (55.2 mg). ^{1}H NMR (300 MHz, $CDCl_3$) δ (ppm): 7.33 (s, 1H), 7.03-7.16 (m, 4H), 5.12-5.16 (t, 1H), 4.82-4.91 (m, 2H), 3.67-3.86 (dd, 2H), 3.45-3.54 (m, 2H), 3.26-3.34 (m, 1H),

2.86-2.97 (m, 4H), 2.65-2.73 (m, 1H), 2.28 (s, 3H), 1.21-1.79 (m, 12H), 0.90-0.94 (t, 3H). ^{13}C NMR δ (ppm): 168.64, 136.91, 133.92, 131.99, 129.23, 128.95, 128.49, 125.26, 120.23, 63.80, 61.98, 58.88, 56.59, 52.58, 43.90, 28.55, 28.46, 28.27, 27.82, 25.17, 24.58, 22.57, 22.45, 17.74, 14.69.

Methyl 2-(4-(cyclohexyl(3,4-dihydroisoquinolin-2(1H)-yl)methyl)-1H-1,2,3-triazol-1-yl)-2-phenylacetate (**Es-19**). Prepared according to General procedure **B**, using **Az-9** (0.2 mmol, 38.2 mg) and **PA-2** (0.2 mmol, 50.7 mg). 10h. Flash chromatography was carried out using a petroleum ether/EtOAc mixture as the eluent (1:1) to afford a yellow oil in 51% yield (45.4 mg). ^{1}H NMR (400 MHz, $CDCl_3$) δ (ppm): 7.02-7.28 (m, 9H), 6.84 (s, 1H), 3.62-3.83 (m, 3H), 3.18 (s, 3H), 2.87-2.93 (m, 4H), 2.59-2.63 (m, 1H), 0.85-1.38 (m, 10H). ^{13}C NMR δ (ppm): 173.18, 136.84, 136.63, 135.12, 133.33, 129.77 (2C), 129.50 (4C), 129.38, 127.08, 124.41, 123.08, 63.71, 56.04, 55.76, 50.09, 37.26, 32.80, 31.03, 29.81, 28.14, 27.88, 27.79, 25.12.

Methyl 2-(4-(cyclohexyl(3,4-dihydroisoquinolin-2(1H)-yl)methyl)-1H-1,2,3-triazol-1-yl)-3-hydroxypropanoate (**Es-20**). Prepared according to General procedure **B**, using **Az-10** (0.2 mmol, 29.0 mg) and **PA-2** (0.2 mmol, 50.7 mg). 9h. Flash chromatography was carried out using a petroleum ether/EtOAc mixture as the eluent (7:3) to afford a yellow oil in 77% yield (61.2 mg). ^{1}H NMR (400 MHz, $CDCl_3$) δ (ppm): 7.11 (s, 1H), 6.91-7.01 (m, 4H), 5.43-5.47 (t, 1H), 4.03 (s, 3H), 3.81-3.88 (t, 1H), 3.50-3.68 (dd, 2H), 3.30-3.32 (d, 2H), 2.68-2.79 (m, 4H), 2.45-2.55 (m, 1H), 1.08-1.52 (m, 10H). ^{13}C NMR δ (ppm): 168.39, 136.37, 136.05, 135.12, 128.30, 128.21, 128.01, 126.42, 123.72, 64.42, 63.31, 58.86, 54.20, 53.94, 51.18, 40.36, 29.46, 29.33, 29.15, 25.56, 25.47, 24.62.

Methyl 2-(4-(cyclohexyl(3,4-dihydroisoquinolin-2(1H)-yl)methyl)-1H-1,2,3-triazol-1-yl)-3-hydroxybutanoate (**Es-21**). Prepared according to General procedure **B**, using **Az-11** (0.2 mmol, 32.0 mg) and **PA-2** (0.2 mmol, 50.7 mg). 8h. Flash chromatography was carried out using a petroleum ether/EtOAc mixture as the eluent (7:3) to afford a yellow oil in 71% yield (58.7 mg). ^{1}H NMR (300 MHz, $CDCl_3$) δ (ppm): 7.04-7.19 (m, 5H), 5.13-5.14 (d, 1H), 3.92

(s, 3H), 3.61-3.84 (m, 6H), 2.83-2.89 (m, 4H), 2.57-2.67 (m, 1H), 2.28-2.36 (d, 3H), 1.36-1.75 (m, 10H). ^{13}C NMR δ (ppm): 170.34, 136.47, 136.15, 135.22, 129.83, 129.22, 128.31, 126.12, 123.82, 64.26, 63.53, 58.16, 54.39, 53.44, 50.40, 39.24, 29.80, 29.68, 29.49, 26.58, 25.91, 25.81, 18.15.

Methyl 2-(4-(cyclohexyl(3,4-dihydroisoquinolin-2(1H)-yl)methyl)-1H-1,2,3-triazol-1-yl)-3-(1H-indol-3-yl)propanoate (**Es-22**). Prepared according to General procedure **B**, using **Az-12** (0.2 mmol, 48.9 mg) and **PA-2** (0.2 mmol, 50.7 mg). 7h. Flash chromatography was carried out using a petroleum ether/EtOAc mixture as the eluent (1:1) to afford a yellow oil in 58% yield (58.1 mg). ^{1}H NMR (400 MHz, $CDCl_3$) δ (ppm): 7.89-7.92 (m, 1H), 7.00-7.12 (m, 5H), 6.84-6.96 (m, 4H), 4.92-4.94 (t, 1H), 3.75-3.76 (t, 1H), 3.69 (s, 3H), 3.36-3.57 (dd, 2H), 2.97-2.99 (d, 2H), 2.64-2.70 (m, 4H), 2.39-2.44 (m, 1H), 1.41-2.09 (m, 10H). ^{13}C NMR δ (ppm): 171.23, 136.47, 135.95, 134.82, 134.61, 129.56, 128.00 (2C), 127.87, 127.76, 127.49, 127.37, 125.07, 122.40, 121.06, 113.01, 110.69, 62.28, 60.46, 59.55, 55.07, 52.39, 51.06, 40.69, 30.14, 30.02, 27.12, 26.92, 26.25, 25.31.

Methyl 2-(4-(cyclohexyl(3,4-dihydroisoquinolin-2(1H)-yl)methyl)-1H-1,2,3-triazol-1-yl)-3-(4-hydroxyphenyl)propanoate (**Es-23**). Prepared according to General procedure **B**, using **Az-13** (0.2 mmol, 44.2 mg) and **PA-2** (0.2 mmol, 50.7 mg). 6h. Flash chromatography was carried out using a petroleum ether/EtOAc mixture as the eluent (1:1) to afford a yellow oil in 51% yield (48.4 mg). ^{1}H NMR (400 MHz, $CDCl_3$) δ (ppm): 7.24 (s, 1H), 7.14-7.18 (m, 4H), 6.79-6.84 (d, 2H), 6.56-6.60 (d, 2H), 5.30 (s, 1H), 4.10-4.15 (dd, 1H), 3.84 (s, 3H), 3.52-3.73 (dd, 2H), 3.12-3.18 (t, 1H), 2.55-2.86 (dd, 2H), 2.01-2.10 (m, 4H), 0.86-1.76 (m, 11H). ^{13}C NMR δ (ppm): 171.24, 164.94, 137.22, 135.45, 134.23, 132.56, 129.54, 129.45 (2C), 129.26, 128.81, 126.16, 125.57, 116.11, 115.68, 64.66, 58.60, 52.93, 51.90, 51.77, 39.69, 38.95, 29.81, 29.72, 28.87, 26.25, 25.87, 25.83.

Methyl 2-(4-(cyclohexyl(3,4-dihydroisoquinolin-2(1H)-yl)methyl)-1H-1,2,3-triazol-1-yl)-5-guanidinopentanoate (**Es-24**). Prepared according to General procedure **B**, using **Az-15** (0.2 mmol, 43.0 mg) and **PA-2** (0.2 mmol, 50.7 mg). 48h. Flash chromatography was carried

out using methanol as the eluent to afford a yellow solid in 28% yield (26.3 mg). ^{1}H NMR (400 MHz, $CDCl_3$) δ (ppm): 9.02 (s, 1H), 7.96-8.22 (m, 4H), 7.32 (s, 1H), 7.00-7.11 (m, 4H), 5.48-5.58 (m, 1H), 3.84 (s, 3H), 3.55-3.75 (dd, 2H), 3.14-3.17 (m, 2H), 2.77-2.87 (m, 4H), 2.58-2.62 (m, 1H), 2.02-2.12 (m, 2H), 1.62-1.80 (m, 5H), 0.87-1.34 (m, 7H). ^{13}C NMR δ (ppm): 174.84, 151.58, 139.44, 139.16, 138.71, 129.57, 129.47, 128.63, 126.01, 125.58, 66.43, 58.00, 52.33, 51.30, 51.17, 41.85, 39.09, 29.89, 29.21, 29.12, 28.27, 25.65, 25.27, 25.23 (2C).

2-(4-(((Benzo[d][1,3]dioxol-5-ylmethyl)amino)(cyclohexyl)methyl)-1H-1,2,3-triazol-1-yl)-3-phenylpropanal (**Ald-1**). Prepared according to the General procedure **C**, starting from **Es-1** (131.2 mg, 0.27 mmol). 3 h. Column chromatography was carried out using a petroleum ether/EtOAc gradient (9:1 – 3:2). Yellowish oil; yield: 42.4 mg (36%). ^{1}H NMR (300 MHz, $CDCl_3$) δ (ppm): 9.61 (s, 1H), 7.34 (s, 1H), 7.10-7.21 (m, 5H), 6.81 (s, 1H), 6.67-6.73 (m, 2H), 5.92 (s, 2H), 5.48-5.51 (q, 1H), 3.61-3.63 (d, 2H), 3.30-3.54 (m, 3H), 0.83-1.77 (m, 11H). ^{13}C NMR δ (ppm): 197.25, 148.77, 147.48, 146.37, 139.79, 134.63, 128.67, 128.35, 128.21, 127.72, 127.38, 126.27, 121.47, 109.78, 107.46, 101.04, 60.17, 57.85, 51.43, 43.09, 38.65, 28.99, 28.86, 27.90, 26.40, 26.36. HRMS: *m/z* $[M+H]^+$ calcd. for $C_{26}H_{30}N_4O_3$: 446.2318; found: 447.2391.

2-(4-(((Benzo[d][1,3]dioxol-5-ylmethyl)amino)(cyclohexyl)methyl)-1H-1,2,3-triazol-1-yl)-3-mehtylbutanal (**Ald-2**). Prepared according to the General procedure **C**, starting from **Es-2** (73.2 mg, 0.17 mmol). 3 h. Column chromatography was carried out using a petroleum-ether/ethylacetate gradient (9:1 – 3:2). Clear oil with yellowish hue; yield: 35.9 mg (53%). ^{1}H NMR (400 MHz, $CDCl_3$) δ (ppm): 9.78 (s, 1H), 7.31 (s, 1H), 6.81 (s, 1H), 6.67-6.74 (m, 2H), 5.92 (s, 2H), 5.17 (dd, 1H), 4.24-4.26 (d, 1H), 3.58 (dd, 2H), 2.16-2.39 (m, 1H) 1.08-1.74 (m, 11H), 0.87-0.89 (m, 6H). ^{13}C NMR δ (ppm): 199.12, 147.35, 146.21, 139.75, 133.96, 120.95, 119.98, 108.72, 107.76, 100.58, 59.72, 55.11, 51.08, 42.07, 29.83, 28.39, 26.15, 25.84, 25.72 (2C), 19.08, 18.04. HRMS: *m/z* $[M+H]^+$ calcd. for $C_{22}H_{30}N_4O_3$: 398.2318; found: 399.2391.

4-(4-(((Benzo[d][1,3]dioxol-5-ylmethyl)amino)(cyclohexyl)methyl)-1H-1,2,3-triazol-1-yl)-5-

oxopentanoic acid (**Ald-3**). Prepared according to the General procedure **C**, starting from **Es-3** (45.9 mg, 0.1 mmol). 2.5 h. Flash chromatography was carried out using a petroleum ether/acetone mixture as the eluent (4:1). Yellow solid; yield: 10.2 mg (21%). ^{1}H NMR (300 MHz, $CDCl_3$) δ (ppm): 9.69 (s, 1H), 7.38 (s, 1H), 6.63-6.76 (m, 3H), 5.91 (s, 2H), 5.50-5.57 (t, 1H), 3.63-3.66 (d, 1H), 3.40-3.59 (dd, 2H), 2.48-2.52 (t, 2H), 2.19-2.24 (t, 2H), 1.82-1.90 (m, 1H), 0.85-1.52 (m, 10H). ^{13}C NMR δ (ppm): 198.23, 180.19, 149.64, 145.98, 145.93, 134.79, 126.27, 120.55, 108.27, 108.03, 101.03, 60.07, 56.00, 51.17, 43.05, 36.40, 30.80, 30.75, 29.39, 26.70, 26.13, 25.76. HRMS: *m/z* $[M+H]^+$ calcd. for $C_{22}H_{28}N_4O_5$: 428.2060; found: 429.2132.

2-(4-(((Benzo[d][1,3]dioxol-5-ylmethyl)amino)(cyclohexyl)methyl)-1H-1,2,3-triazol-1-yl)-3-(1H-imidazol-4-yl)propanal (**Ald-4**). Prepared according to the General procedure **C**, starting from **Es-4** (56.7 mg, 0.12 mmol). 5 h. Column chromatography was carried out using a petroleum ether/EtOAc/methanol gradient (9:1:0 – 2:7:1). Yellow solid; yield: 22.1 mg (42%). ^{1}H NMR (300 MHz, $CDCl_3$) δ (ppm): 9.43 (s, 1H), 7.98-8.00 (d, 1H), 7.42 (s, 1H), 6.93-6.95 (d, 1H), 6.82 (s, 1H), 6.72-6.75 (m, 2H), 5.92 (s, 2H), 5.80-5.83 (t, 1H), 3.78-3.80 (d, 1H), 3.66-3.69 (d, 2H), 3.51-4.11 (dd, 2H), 0.85-1.92 (m, 11H). ^{13}C NMR δ (ppm): 195.34, 149.97, 146.99, 146.24, 134.98, 134.39, 130.32, 127.75, 120.88, 117.13, 108.43, 108.01, 99.96, 59.25, 56.43, 51.47, 43.02, 30.69, 29.81, 29.39, 26.51, 26.20, 26.15. HRMS: *m/z* $[M+H]^+$ calcd. for $C_{23}H_{28}N_6O_3$: 436.2223; found: 437.2296.

2-(4-(((Benzo[d][1,3]dioxol-5-ylmethyl)amino)(cyclohexyl)methyl)-1H-1,2,3-triazol-1-yl)-4-methylpentanal (**Ald-5**). Prepared according to the General procedure **C**, starting from **Es-5** (79.6 mg, 0.18 mmol). 5 h. Column chromatography was carried out using a petroleum ether/EtOAc gradient (9:1 – 7:3). Yellow solid; yield: 24.0 mg (32%). ^{1}H NMR (300 MHz, $CDCl_3$) δ (ppm): 9.18 (s, 1H), 7.07 (s, 1H), 6.74 (s, 1H), 6.55-6.59 (m, 2H), 5.74 (s, 2H), 5.33-5.36 (t, 1H), 3.51-3.54 (d, 1H), 3.51-3.73 (dd, 2H), 1.78-2.00 (dd, 2H), 1.10-1.43 (m, 12H), 0.94-0.97 (d, 6H). ^{13}C NMR δ (ppm): 197.06, 149.89, 147.61, 146.85, 134.46, 128.02, 121.97, 109.01, 108.04, 100.68, 59.46, 56.68, 51.68, 42.97, 35.66, 29.77, 29.40, 26.49, 26.21,

26.13, 24.61, 22.11 (2C). HRMS: m/z $[M+H]^+$ calcd. for $C_{23}H_{32}N_4O_3$: 412.2474; found: 413.2547.

6-Amino-2-(4-(((benzo[d][1,3]dioxol-5-ylmethyl)amino)(cyclohexyl)methyl)-1H-1,2,3-triazol-1-yl)hexanal (**Ald-6**). Prepared according to the General procedure **C**, starting from **Es-6** (44.0 mg, 0.1 mmol). 4 h. Column chromatography was carried out using a petroleum ether/EtOAc/methanol gradient (7:3:0 – 2:7:1). Brown oil; yield: 25.2 mg (51%). ^{1}H NMR (400 MHz, $CDCl_3$) δ (ppm): 9.18 (s, 1H), 7.07 (s, 1H), 6.68 (s, 1H), 6.57-6.60 (d, 2H), 5.74 (s, 2H), 5.42-5.45 (t, 1H), 3.96-3.98 (d, 1H), 3.51-3.73 (dd, 2H), 3.12-3.16 (t, 2H), 2.14-2.20 (q, 2H), 0.69-1.45 (m, 15H). ^{13}C NMR δ (ppm): 190.45, 153.27, 148.75, 148.01, 135.95, 128.82, 121.73, 108.52, 107.09, 101.52, 58.35, 57.13, 51.53, 43.27, 40.18, 29.03, 27.93, 27.88, 26.52, 26.07, 26.02, 25.26, 23.26. HRMS: m/z $[M+H]^+$ calcd. for $C_{23}H_{33}N_5O_3$: 427.2583; found: 428.2656.

2-(4-(((Benzo[d][1,3]dioxol-5-ylmethyl)amino)(cyclohexyl)methyl)-1H-1,2,3-triazol-1-yl)-4-(methylthio)butanal (**Ald-7**). Prepared according to the General procedure **C**, starting from **Es-7** (80.0 mg, 0.17 mmol). 3 h. Column chromatography was carried out using a petroleum ether/EtOAc gradient (9:1 – 3:2). Yellowish oil; yield: 43.0 mg (59%). ^{1}H NMR (300 MHz, $CDCl_3$) δ (ppm): 9.73 (s, 1H), 7.69 (s, 1H), 6.79-7.06 (m, 3H), 5.96 (s, 2H), 5.32-5.38 (m, 1H), 4.10-4.29 (m, 3H), 3.59-3.70 (m, 2H), 2.31 (s, 3H), 2.03-2.06 (m, 2H), 0.64-1.73 (m, 11H). ^{13}C NMR δ (ppm): 197.12, 149.68, 147.05, 146.13, 134.22, 127.65, 121.13, 109.21, 108.19, 101.13, 59.44, 56.71, 51.58, 43.17, 31.38, 29.86, 29.47, 29.41, 26.54, 26.31, 26.11, 16.84. HRMS: m/z $[M+H]^+$ calcd. for $C_{22}H_{30}N_4O_3S$: 430.2039; found: 431.2111.

2-(4-(((Benzo[d][1,3]dioxol-5-ylmethyl)amino)(cyclohexyl)methyl)-1H-1,2,3-triazol-1-yl) propanal (**Ald-8**). Prepared according to the General procedure **C**, starting from **Es-8** (61.7 mg, 0.17 mmol). 2 h. Column chromatography was carried out using a petroleum ether/EtOAc gradient (9:1 – 4:1). Pale yellow solid; yield: 23.6 mg (38%). ^{1}H NMR (300 MHz, $CDCl_3$) δ (ppm): 9.78 (s, 1H), 7.31 (s, 1H), 6.81 (s, 1H), 6.70-6.71 (d, 2H), 5.92 (s, 2H), 5.45-5.47 (q, 1H) 3.60-3.63 (d, 1H), 3.39-3.59 (dd, 2H), 1.03-1.70 (m, 14H). ^{13}C NMR δ

(ppm): 199.36, 150.15, 147.59, 146.37, 134.48, 127.87, 121.15, 108.69, 107.97, 100.81, 59.39, 56.62, 51.49, 43.04, 29.82, 29.44, 26.50, 26.22, 26.16, 20.89. HRMS: *m/z* $[M+H]^+$ calcd. for $C_{20}H_{26}N_4O_3$: 370.2005; found: 371.2078.

2-(4-(((Benzo[d][1,3]dioxol-5-ylmethyl)amino)(cyclohexyl)methyl)-1H-1,2,3-triazol-1-yl)-2-phenylacetaldehyde (**Ald-9**). Prepared according to the General procedure **C**, starting from **Es-9** (75.8 mg, 0.16 mmol). 3 h. Column chromatography was carried out using a petroleum ether/EtOAc gradient (9:1 – 7:3). Yellow oil; yield: 26.3 mg (37%). ^{1}H NMR (300 MHz, $CDCl_3$) δ (ppm): 9.35 (s, 1H), 7.64-7.71 (m, 5H), 7.54 (s, 1H), 6.86 (s, 1H), 6.72-6.80 (dd, 2H), 6.34-6.36 (d, 1H), 5.91 (s, 2H), 3.73-3.76 (d, 1H), 3.21-3.45 (dd, 2H), 1.84-1.91 (m, 1H), 1.08-1.57 (m, 10H). ^{13}C NMR δ (ppm): 197.81, 150.14, 147.60, 146.37, 139.85, 134.48, 128.12 (2C), 127.87, 127.64 (2C), 127.30, 120.88, 108.68, 107.86, 101.09, 59.36, 57.33, 52.46, 43.00, 29.12, 28.79, 25.01, 24.33, 24.19. HRMS: *m/z* $[M+H]^+$ calcd. for $C_{25}H_{28}N_4O_3$: 432.2161; found: 433.2234.

2-(4-(((Benzo[d][1,3]dioxol-5-ylmethyl)amino)(cyclohexyl)methyl)-1H-1,2,3-triazol-1-yl)-3-hydroxypropanal (**Ald-10**). Prepared according to the General procedure **C**, starting from **Es-10** (72.5 mg, 0.17 mmol). 3 h. Column chromatography was carried out using a petroleum ether/EtOAc gradient (9:1 – 3:2). Brownwish oil; yield: 37.8 mg (56%). ^{1}H NMR (400 MHz, $CDCl_3$) δ (ppm): 9.18 (s, 1H), 7.07 (s, 1H), 6.68 (s, 1H), 6.57-6.59 (d, 2H), 5.74 (s, 2H), 5.44-5.49 (t, 1H), 3.88-3.92 (d, 2H), 3.69-3.73 (d, 1H), 3.16-3.40 (dd, 2H), 2.83 (broad s, 1H), 1.69-1.73 (m, 1H), 1.17-1.53 (m, 10H). ^{13}C NMR δ (ppm): 196.09, 150.45, 147.28, 146.27, 133.95, 126.90, 121.48, 110.52, 110.33, 100.95, 66.19, 58.94, 55.16, 51.68, 42.56, 29.04, 28.92, 25.56, 25.40, 25.33. HRMS: *m/z* $[M+H]^+$ calcd. for $C_{20}H_{26}N_4O_4$: 386.1954; found: 387.2027.

2-(4-(((Benzo[d][1,3]dioxol-5-ylmethyl)amino)(cyclohexyl)methyl)-1H-1,2,3-triazol-1-yl)-3-hydroxybutanal (**Ald-11**). Prepared according to the General procedure **C**, starting from **Es-11** (56.2 mg, 0.13 mmol). 3 h. Column chromatography was carried out using a petroleum ether/EtOAc gradient (9:1 – 3:2). Brownwish oil; yield: 23.1 mg (44%). ^{1}H NMR (300

MHz, $CDCl_3$) δ (ppm): 9.69 (s, 1H), 7.46 (s, 1H), 6.84 (s, 1H), 6.68-6.77 (d, 2H), 5.92 (s, 2H), 5.85-5.87 (d, 1H), 4.10-4.15 (m, 1H), 3.79-3.81 (d, 1H), 3.52-3.71 (dd, 2H), 2.50 (broad s, 1H), 1.08-1.78 (m, 11H), 0.90-0.92 (d, 3H). ^{13}C NMR δ (ppm): 198.51, 150.88, 147.93, 147.01, 134.97, 127.34, 121.17, 108.64, 108.61, 100.37, 66.67, 60.44, 57.39, 51.22, 44.50, 31.69, 31.65, 25.29, 25.13, 25.06, 19.49. HRMS: *m/z* $[M+H]^+$ calcd. for $C_{21}H_{28}N_4O_4$: 400.2111; found: 401.2183.

2-(4-(((Benzo[d][1,3]dioxol-5-ylmethyl)amino)(cyclohexyl)methyl)-1H-1,2,3-triazol-1-yl)-3-(1H-indol-3-yl)propanal (**Ald-12**). Prepared according to the General procedure **C**, starting from **Es-12** (69.8 mg, 0.14 mmol). 4 h. Column chromatography was carried out using a petroleum ether/EtOAc gradient (9:1 – 3:2). Yellow solid; yield: 38.1 mg (58%). ^{1}H NMR (400 MHz, $CDCl_3$) δ (ppm): 9.62 (s, 1H), 7.98-8.43 (dd, mH), 7.97 (s, 1H), 6.82 (s, 1H), 6.70-6.75 (d, 2H), 5.92 (s, 2H), 5.08-5.11 (t, 1H), 4.08-4.15 (m, 2H), 3.78-3.80 (d, 1H), 3.51-3.69 (dd, 2H), 0.90-1.94 (m, 11H). ^{13}C NMR δ (ppm): 197.31, 148.75, 147.88, 147.84, 136.80, 135.58, 129.98, 129.93, 128.12, 128.08, 124.87, 123.10, 122.56, 112.49, 112.44, 108.39, 107.82, 98.76, 58.30, 57.08, 50.07, 44.06, 29.49, 29.44, 28.08, 26.81, 26.64, 25.39. HRMS: *m/z* $[M+H]^+$ calcd. for $C_{28}H_{31}N_5O_3$: 485.2427; found: 486.2500.

2-(4-(((Benzo[d][1,3]dioxol-5-ylmethyl)amino)(cyclohexyl)methyl)-1H-1,2,3-triazol-1-yl)-3-(4-hydroxyphenyl)propanal (**Ald-13**). Prepared according to the General procedure **C**, starting from **Es-13** (49.8 mg, 0.1 mmol). 3 h. Column chromatography was carried out using a petroleum ether/EtOAc gradient (9:1 – 7:3). Yellow solid; yield: 20.1 mg (43%). ^{1}H NMR (300 MHz, $CDCl_3$) δ (ppm): 9.58 (s, 1H), 7.13-7.21 (dd, 2H), 7.02 (s, 1H), 6.41 (s, 1H), 6.31-6.36 (dd, 2H), 6.23-6.28 (d, 2H), 5.74 (s, 2H), 5.24-5.25 (t, 1H), 4.12 (broad s, 1H), 3.51-3.56 (d, 1H), 3.32-3.37 (dd, 2H), 2.42-2.47 (d, 2H), 1.53-1.62 (m, 1H), 1.03-1.45 (m, 10H). ^{13}C NMR δ (ppm): 196.23, 153.50, 149.11, 148.37, 136.18, 135.31, 132.28, 130.16 (2C), 126.15, 121.49, 120.01, 119.62, 108.74, 107.32, 101.75, 58.82, 55.73, 50.29, 42.07, 38.80, 29.35, 29.30, 27.45, 26.68, 26.50. HRMS: *m/z* $[M+H]^+$ calcd. for $C_{26}H_{30}N_4O_4$: 462.2267; found: 463.2340.

2-(4-(Cyclohexyl(3,4-dihydroisoquinolin-2(1H)-yl)methyl)-1H-1,2,3-triazol-1-yl)-3-phenyl-propanal (**Ald-16**). Prepared according to the General procedure **C**, starting from **Es-16** (179.4 mg, 0.38 mmol). 3 h. Column chromatography was carried out using a petroleum ether/EtOAc gradient (9:1 – 4:1). Yellow solid; yield: 89.5 mg (55%). ^{1}H NMR (300 MHz, $CDCl_3$) δ (ppm): 9.51 (s, 1H), 7.17-7.35 (m, 10H), 5.45-5.49 (m, 1H), 3.57-3.62 (m, 1H), 2.98-3.20 (m, 4H), 2.17-2.45 (m, 4H), 0.86-1.57 (m, 11H). ^{13}C NMR δ (ppm): 197.32, 136.19, 135.76, 135.41, 130.01, 129.74, 129.55, 129.53, 129.21, 129.12, 128.72, 127.58, 126.77, 126.34, 122.56, 63.54, 57.69, 56.92, 55.10, 37.03, 34.54, 32.54, 31.80, 28.57, 28.13, 27.66, 25.34. HRMS: *m/z* $[M+H]^+$ calcd. for $C_{27}H_{32}N_4O$: 428.2576; found: 429.2649.

2-(4-(Cyclohexyl(3,4-dihydroisoquinolin-2(1H)-yl)methyl)-1H-1,2,3-triazol-1-yl)-3-(1H-imidazol-4-yl)propanal (**Ald-17**). Prepared according to the General procedure **C**, starting from **Es-17** (48.4 mg, 0.11 mmol). 5 h. Column chromatography was carried out using a petroleum ether/EtOAc/methanol gradient (9:1:0 – 2:7:1). Yellow solid; yield: 25.3 mg (56%). ^{1}H NMR (400 MHz, $CDCl_3$) δ (ppm): 9.14 (s, 1H), 8.07-8.08 (d, 1H), 6.99-7.12 (m, 6H), 5.26-5.34 (t, 1H), 3.77-3.84 (t, 1H), 3.63-3.83 (dd, 2H), 3.14-3.18 (dd, 2H), 2.83-2.89 (m, 4H), 2.58-2.65 (m, 1H), 1.60-2.12 (m, 10H). ^{13}C NMR δ (ppm): 196.24, 135.87, 135.29, 134.04, 133.13, 130.58, 128.33, 127.40, 126.83, 125.65, 122.39, 115.77, 66.62, 57.66, 52.61, 49.19, 38.25, 31.09, 29.36, 27.77, 27.18, 25.94, 25.16, 25.03. HRMS: *m/z* $[M+H]^+$ calcd. for $C_{24}H_{30}N_6O_2$: 418.2481; found: 419.2554.

2-(4-(Cyclohexyl(3,4-dihydroisoquinolin-2(1H)-yl)methyl)-1H-1,2,3-triazol-1-yl)-4-(methyl-thio)butanal (**Ald-18**). Prepared according to the General procedure **C**, starting from **Es-18** (55.2 mg, 0.12 mmol). 4 h. Column chromatography was carried out using a petroleum ether/EtOAc gradient (9:1 – 7:3). Yellow solid; yield: 21.6 mg (43%). ^{1}H NMR (300 MHz, $CDCl_3$) δ (ppm): 9.55 (s, 1H), 7.44 (s, 1H), 7.03-7.13 (m, 4H), 5.23-5.30 (t, 1H), 3.64-3.83 (dd, 2H), 3.25-2.34 (m, 3H), 2.87-2.89 (m, 4H), 2.60-2.63 (m, 1H), 2.28 (s, 3H), 1.26-1.78 (m, 12H). ^{13}C NMR δ (ppm): 194.80, 134.37, 131.38, 129.85, 128.02, 127.86, 127.79, 125.27, 122.22, 61.37, 57.58, 55.94, 51.36, 33.75, 29.97, 29.86, 28.33, 26.50, 26.05, 26.00, 25.89, 25.82,

14.53. HRMS: m/z $[M+H]^+$ calcd. for $C_{23}H_{32}N_4OS$: 412.2297; found: 413.2370.

2-(4-(Cyclohexyl(3,4-dihydroisoquinolin-2(1H)-yl)methyl)-1H-1,2,3-triazol-1-yl)-2-phenylacetaldehyde (**Ald-19**). Prepared according to the General procedure **C**, starting from **Es-19** (45.4 mg, 0.1 mmol). 4 h. Column chromatography was carried out using a petroleum ether/EtOAc gradient (9:1 – 7:3). Yellow solid; yield: 20.7 mg (49%). ^{1}H NMR (400 MHz, $CDCl_3$) δ (ppm): 9.39 (s, 1H), 7.36-7.52 (m, 5H), 6.99-7.13 (m, 6H), 3.59-3.81 (dd, 2H), 3.66-3.70 (t, 1H), 2.83-2.90 (m, 4H), 2.56-2.63 (m, 1H), 1.61-2.09 (m, 10H). ^{13}C NMR δ (ppm): 198.25, 137.25, 136.04, 134.10, 131.53, 129.03, 128.58 (4C), 128.54, 127.76, 127.59, 125.76, 123.02, 63.90, 55.38, 54.16, 52.22, 36.54, 31.47, 30.98, 28.78, 28.21, 27.77, 25.46. HRMS: m/z $[M+H]^+$ calcd. for $C_{26}H_{30}N_4O$: 414.2420; found: 415.2492.

2-(4-(Cyclohexyl(3,4-dihydroisoquinolin-2(1H)-yl)methyl)-1H-1,2,3-triazol-1-yl)-3-hydroxypropanal (**Ald-20**). Prepared according to the General procedure **C**, starting from **Es-20** (61.2 mg, 0.15 mmol). 5 h. Column chromatography was carried out using a petroleum ether/EtOAc gradient (9:1 – 1:1). Yellow solid; yield: 21.5 mg (38%). ^{1}H NMR (400 MHz, $CDCl_3$) δ (ppm): 9.37 (s, 1H), 7.52 (s, 1H), 7.03-7.13 (m, 4H), 5.31-5.35 (t, 1H), 3.68-3.86 (dd, 2H), 3.50-3.53 (t, 1H), 3.27-3.31 (d, 2H), 2.90-2.96 (m, 4H), 2.65-2.73 (m, 1H), 1.34-1.79 (m, 10H). ^{13}C NMR δ (ppm): 199.43, 138.59, 136.64, 134.08, 128.89, 128.31, 127.94, 126.11, 120.20, 65.73, 62.64, 57.21, 54.05, 50.34, 39.41, 30.88, 29.67, 27.72, 25.16, 24.07, 24.02. HRMS: m/z $[M+H]^+$ calcd. for $C_{21}H_{28}N_4O_2$: 368.2212; found: 369.2285.

2-(4-(Cyclohexyl(3,4-dihydroisoquinolin-2(1H)-yl)methyl)-1H-1,2,3-triazol-1-yl)-3-hydroxybutanal (**Ald-21**). Prepared according to the General procedure **C**, starting from **Es-21** (58.7 mg, 0.14 mmol). 4 h. Column chromatography was carried out using a petroleum ether/EtOAc gradient (9:1 – 3:2). Yellow solid; yield: 20.2 mg (37%). ^{1}H NMR (300 MHz, $CDCl_3$) δ (ppm): 9.56 (s, 1H), 7.52 (s, 1H), 7.02-7.14 (m, 4H), 5.35-5.39 (m, 1H), 5.27-5.30 (d, 1H), 3.61-3.81 (dd, 2H), 3.26-3.35 (t, 1H), 2.85-2.90 (m, 4H), 2.57-2.64 (m, 1H), 2.23-2.30 (d, 3H), 1.40-1.71 (m, 10H). ^{13}C NMR δ (ppm): 190.10, 136.38, 135.16, 133.22, 128.15, 127.70, 127.66, 125.46, 124.45, 65.89, 62.80, 57.36, 54.20, 50.55, 38.11, 28.41, 27.83, 27.46,

27.40, 25.63, 25.09, 18.78. HRMS: m/z $[M+H]^+$ calcd. for $C_{22}H_{30}N_4O_2$: 382.2369; found: 383.2442.

2-(4-(Cyclohexyl(3,4-dihydroisoquinolin-2(1H)-yl)methyl)-1H-1,2,3-triazol-1-yl)-3-(1H-indol-3-yl)propanal (**Ald-22**). Prepared according to the General procedure **C**, starting from **Es-22** (58.0 mg, 0.12 mmol). 5 h. Column chromatography was carried out using a petroleum ether/EtOAc gradient (9:1 – 7:3). Yellow solid; yield: 27.0 mg (50%). ^{1}H NMR (400 MHz, $CDCl_3$) δ (ppm): 9.61 (s, 1H), 7.37-7.42 (m, 1H), 7.23 (s, 1H), 7.14-7.18 (m, 6H), 6.73-6.79 (m, 2H), 5.34-5.40 (t, 1H), 4.09-4.13 (t, 1H), 3.52-3.73 (dd, 2H), 3.12-3.15 (d, 2H), 2.79-2.87 (m, 4H), 2.54-2.59 (m, 1H), 1.14-1.91 (m, 10H). ^{13}C NMR δ (ppm): 191.23, 136.87, 136.16, 135.59, 135.19, 129.60, 129.25 (2C), 129.16, 128.68, 128.22, 127.43, 124.65, 124.10, 120.83, 112.48, 109.65, 63.42, 61.80, 60.32, 54.73, 50.08, 39.20, 29.11, 28.76, 28.66, 27.73, 26.94, 24.78. HRMS: m/z $[M+H]^+$ calcd. for $C_{29}H_{33}N_5O$: 467.2685; found: 468.2758.

2-(4-(Cyclohexyl(3,4-dihydroisoquinolin-2(1H)-yl)methyl)-1H-1,2,3-triazol-1-yl)-3-(4-hydroxyphenyl)propanal (**Ald-23**). Prepared according to the General procedure **C**, starting from **Es-23** (48.4 mg, 0.1 mmol). 4 h. Column chromatography was carried out using a petroleum ether/EtOAc gradient (9:1 – 4:1). Brownwish solid; yield: 20.4 mg (44%). ^{1}H NMR (400 MHz, $CDCl_3$) δ (ppm): 9.64 (s, 1H), 7.54-7.62 (m, 6H), 7.39 (s, 1H), 6.58-6.63 (d, 2H), 5.10 (s, 1H), 4.83-4.85 (m, 1H), 4.14-4.17 (m, 1H), 3.54-3.75 (dd, 2H), 3.15-3.17 (d, 2H), 2.82-2.88 (m, 4H), 0.85-1.93 (m, 11H). ^{13}C NMR δ (ppm): 196.78, 160.12, 135.77, 135.06, 134.49, 134.09, 131.02 (2C), 128.50, 128.15, 127.58, 127.12, 126.33, 117.13, 116.70, 63.01, 55.74, 54.36, 50.24, 40.51, 37.41, 29.57, 29.47, 28.99, 25.59, 25.12, 24.42. HRMS: m/z $[M+H]^+$ calcd. for $C_{27}H_{32}N_4O_2$: 444.2525; found: 445.2598.

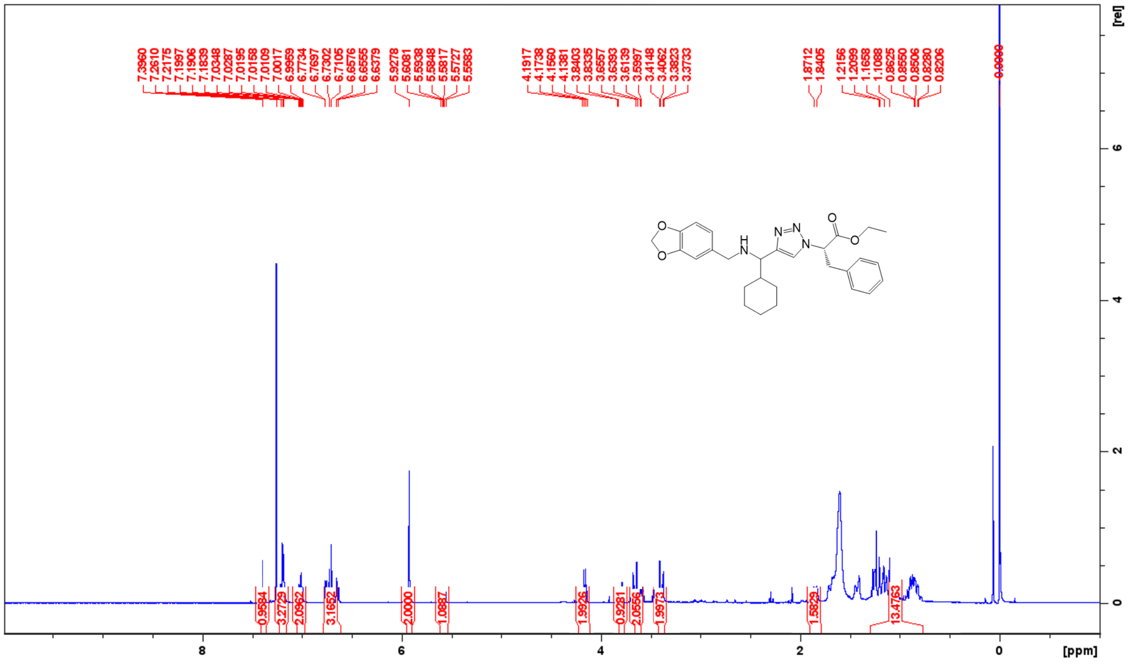

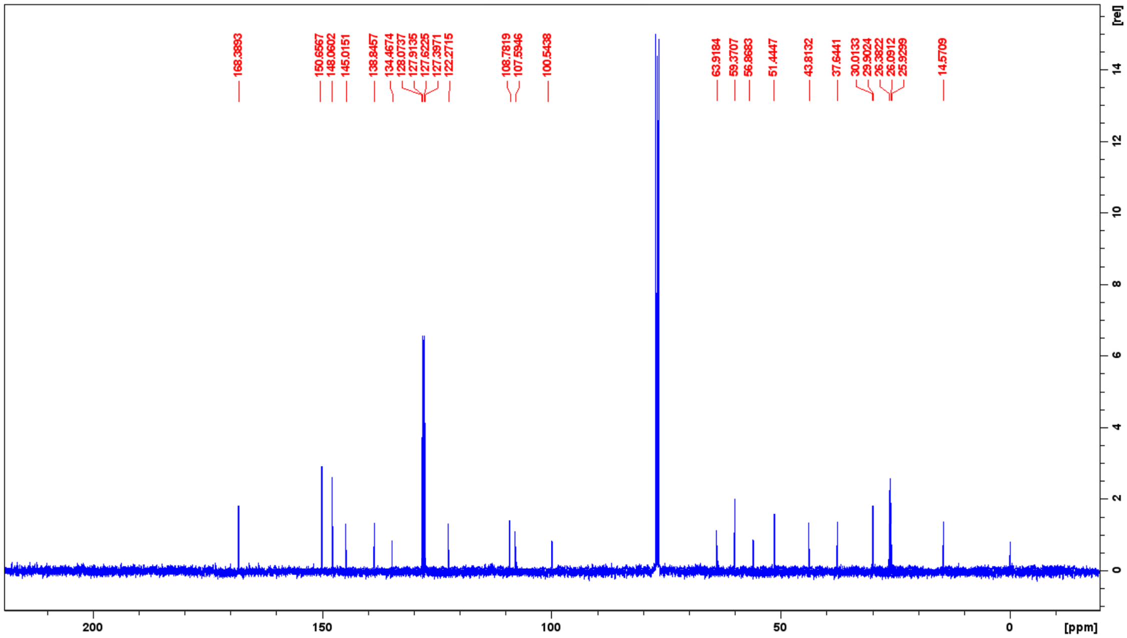

^{1}H NMR and ^{13}C NMR spectra of **Es-1**

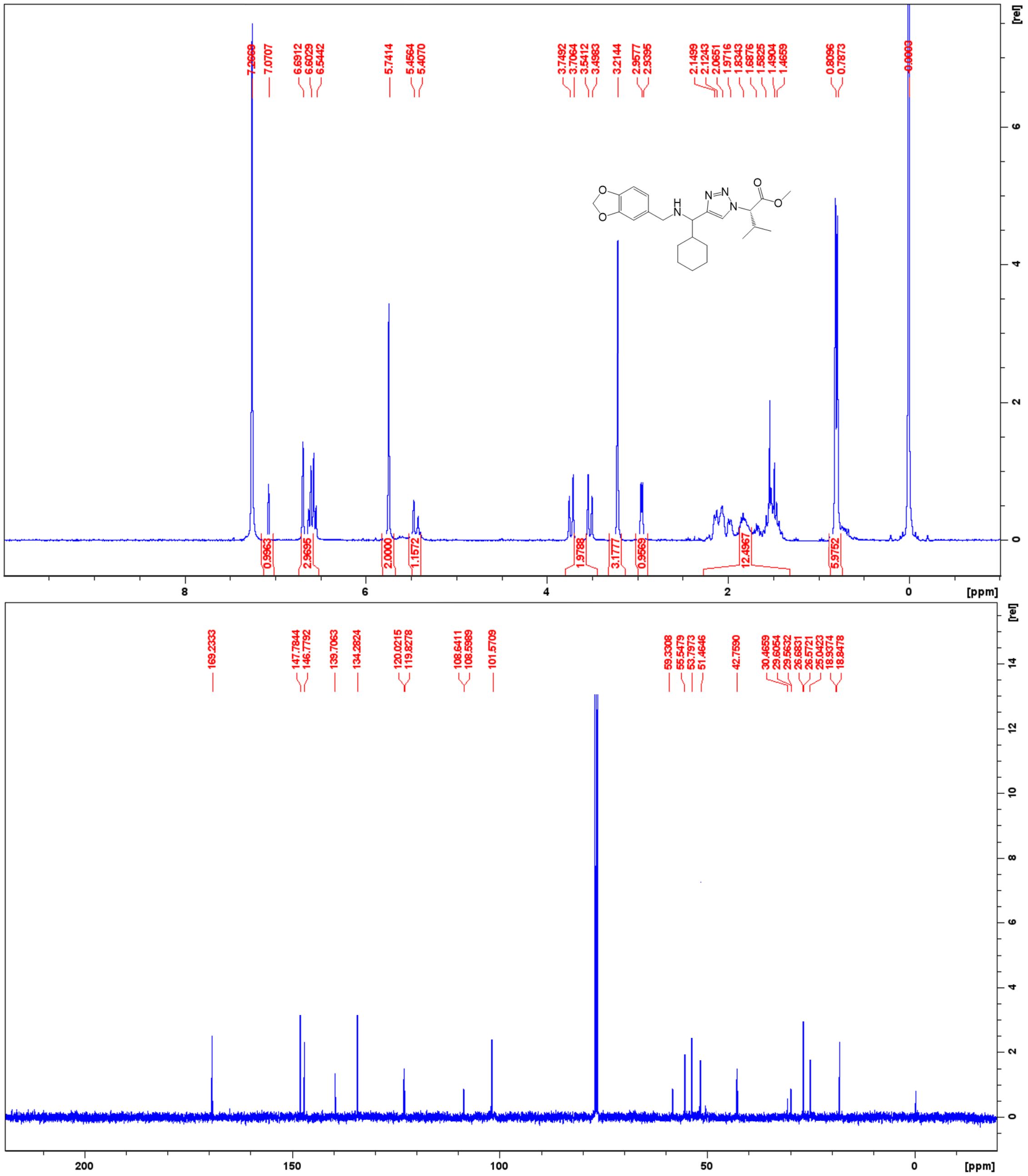

^{1}H NMR and ^{13}C NMR spectra of **Es-2**

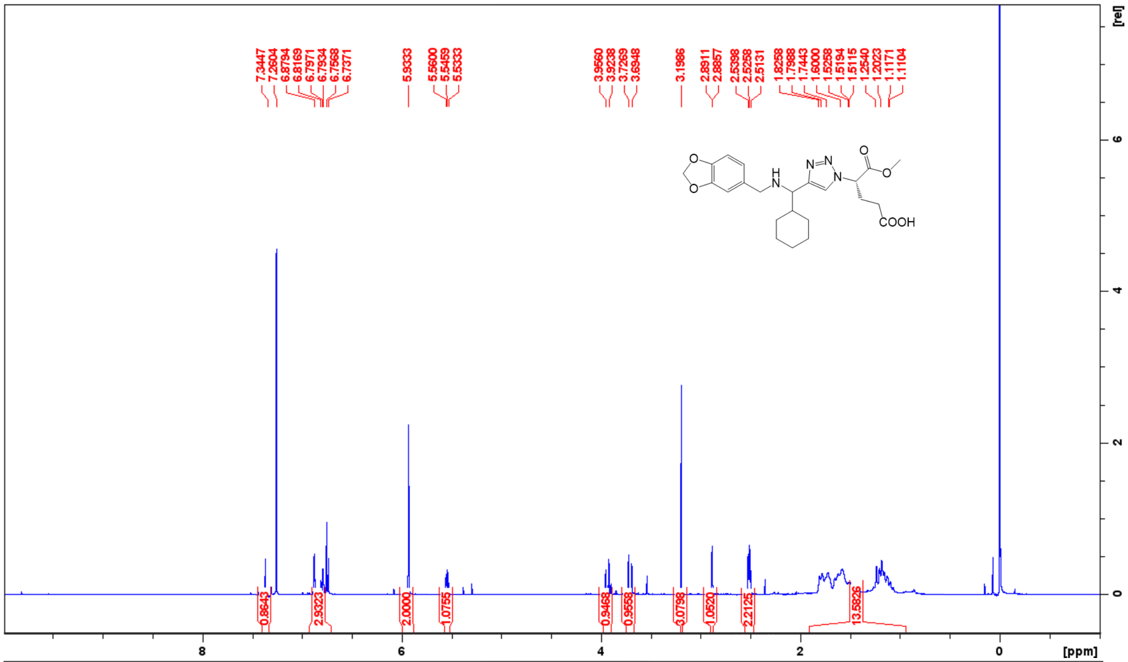

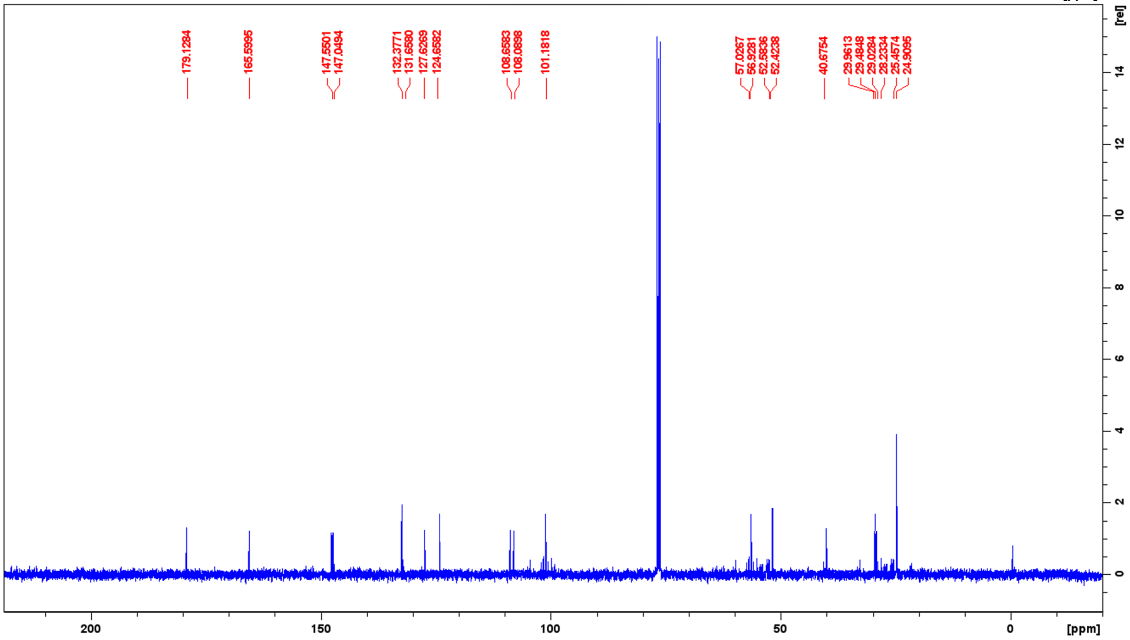

^{1}H NMR and ^{13}C NMR spectra of **Es-3**

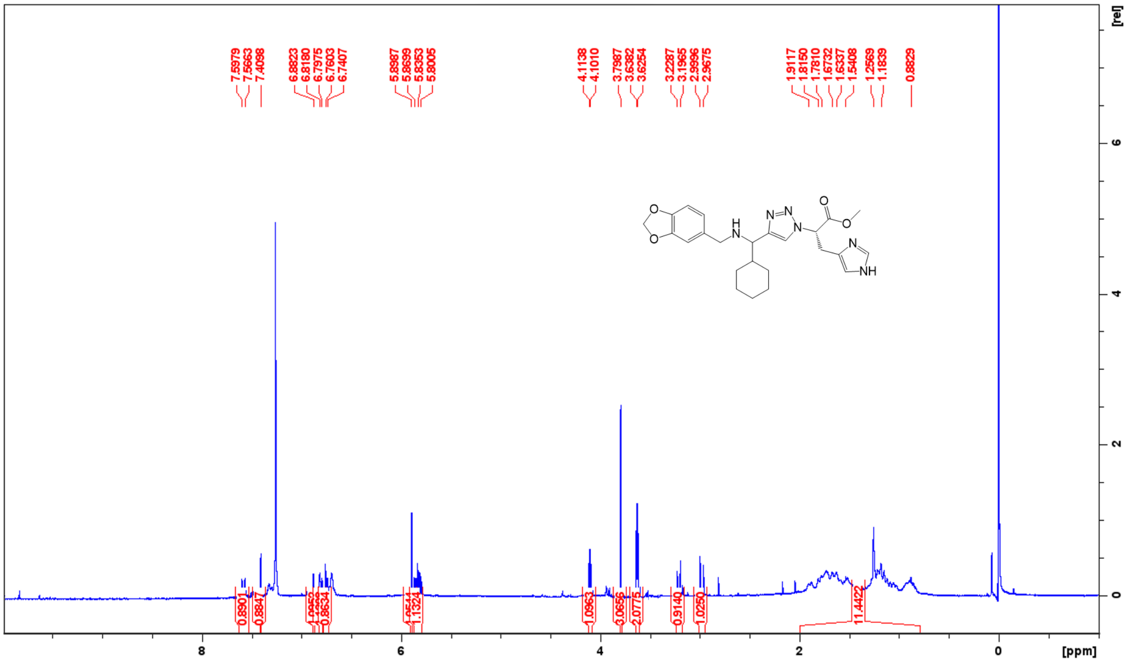

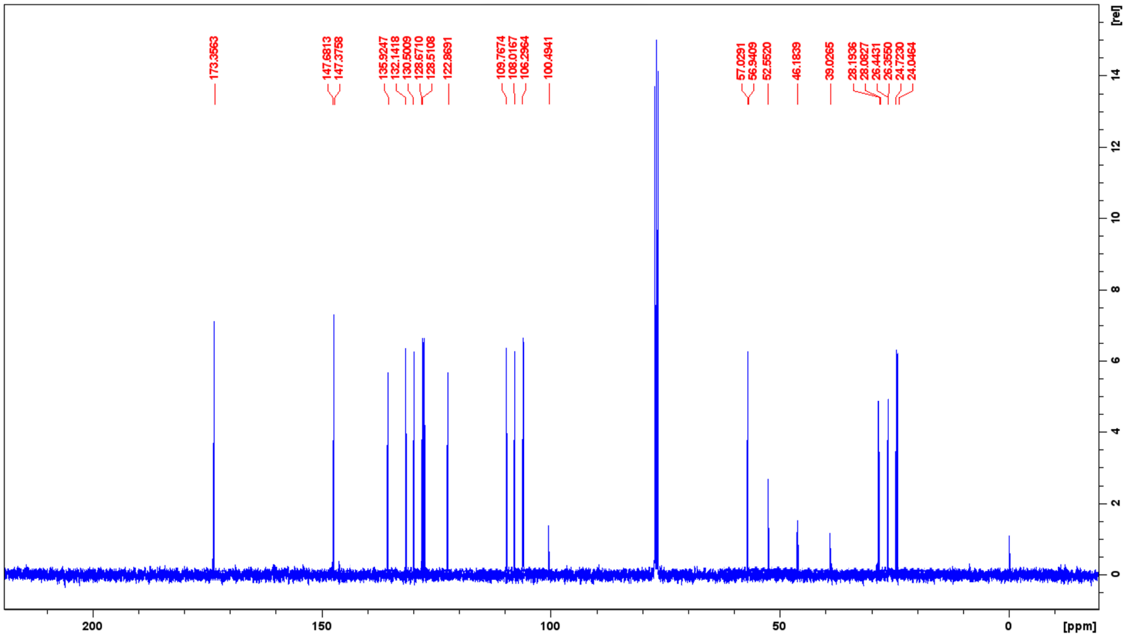

^{1}H NMR and ^{13}C NMR spectra of **Es-4**

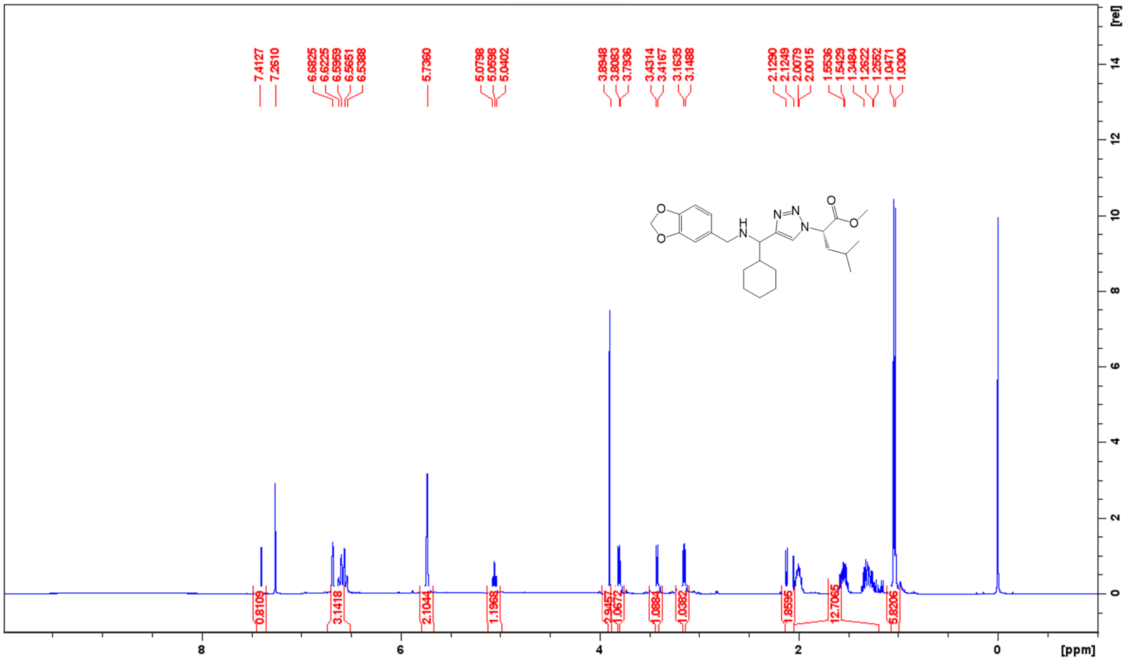

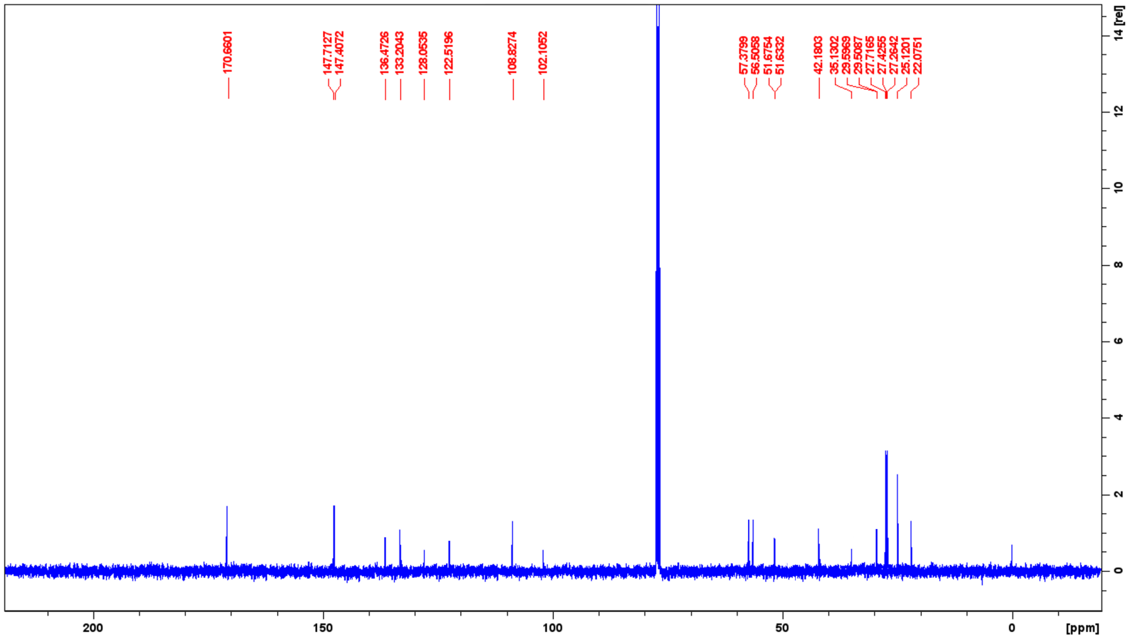

^{1}H NMR and ^{13}C NMR spectra of **Es-5**

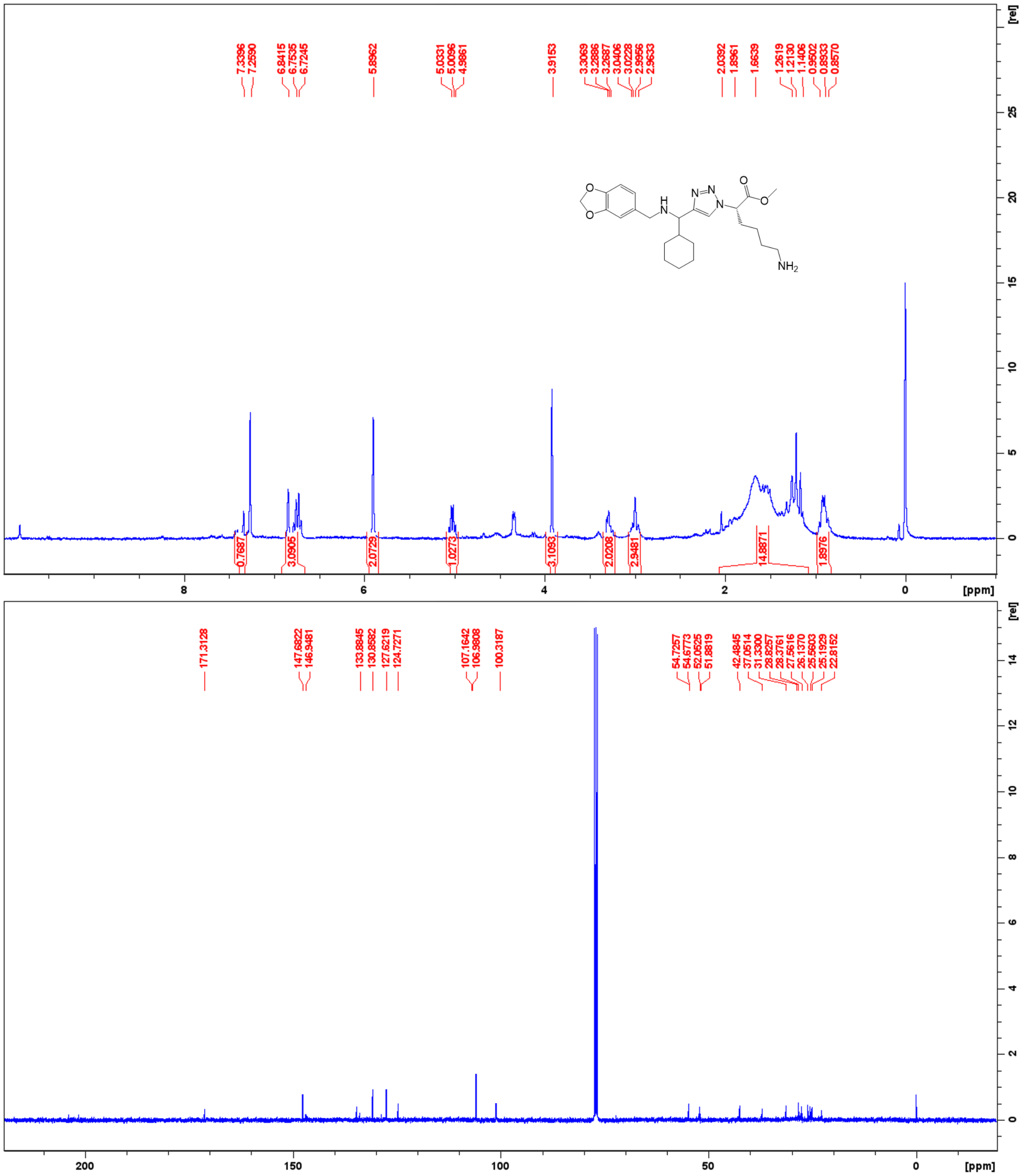

^{1}H NMR and ^{13}C NMR spectra of **Es-6**

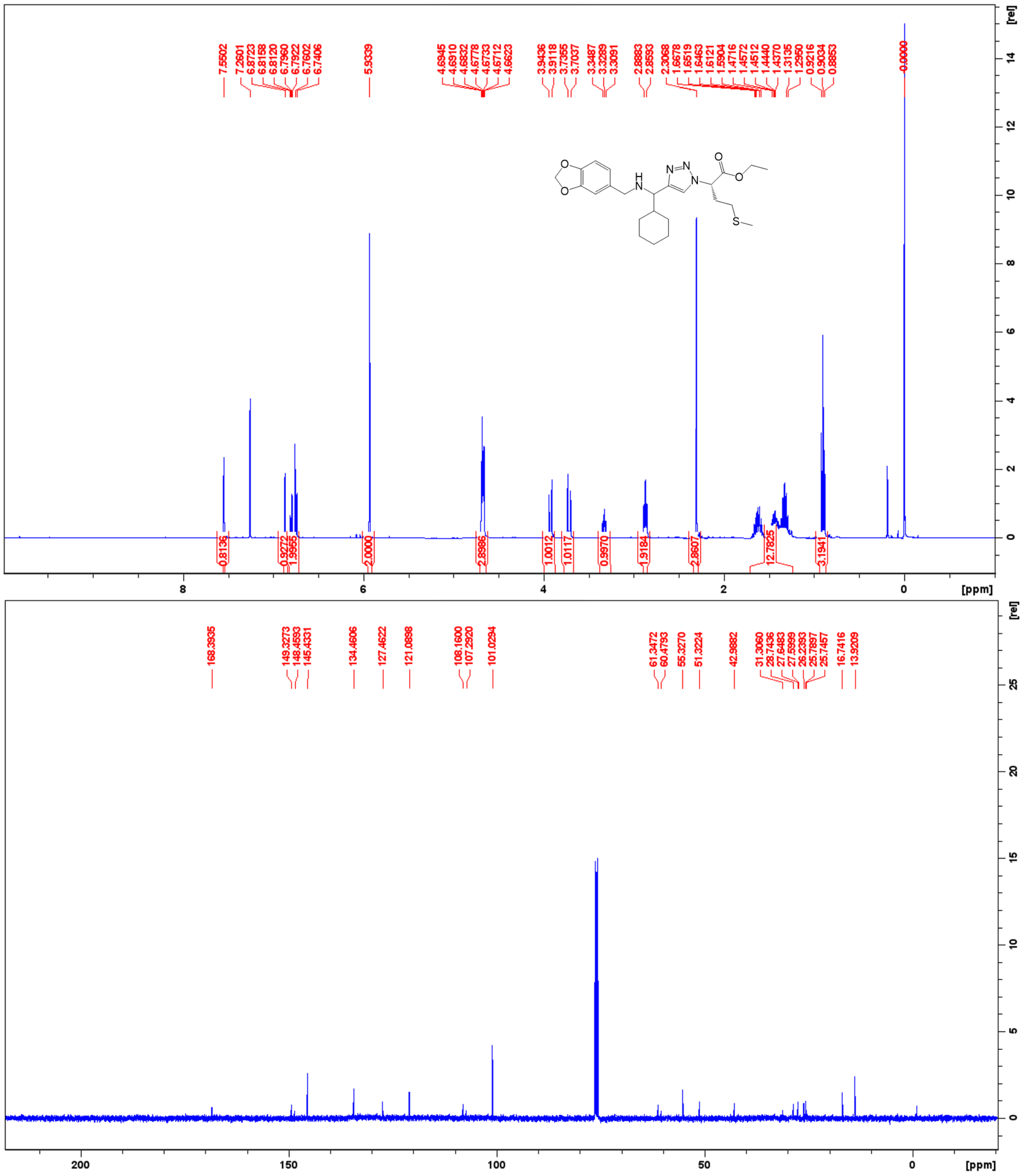

^{1}H NMR and ^{13}C NMR spectra of **Es-7**

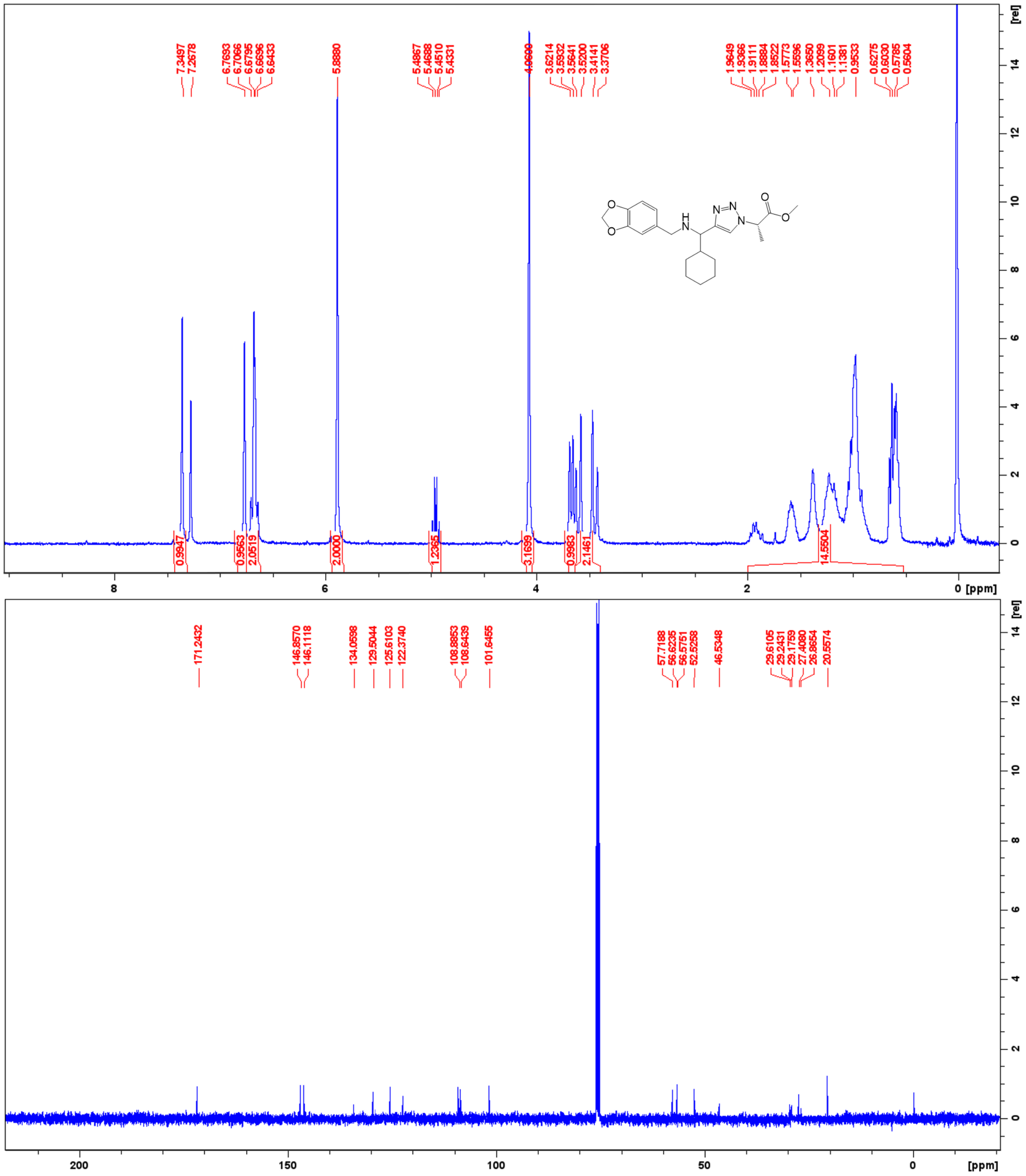

^{1}H NMR and ^{13}C NMR spectra of **Es-8**

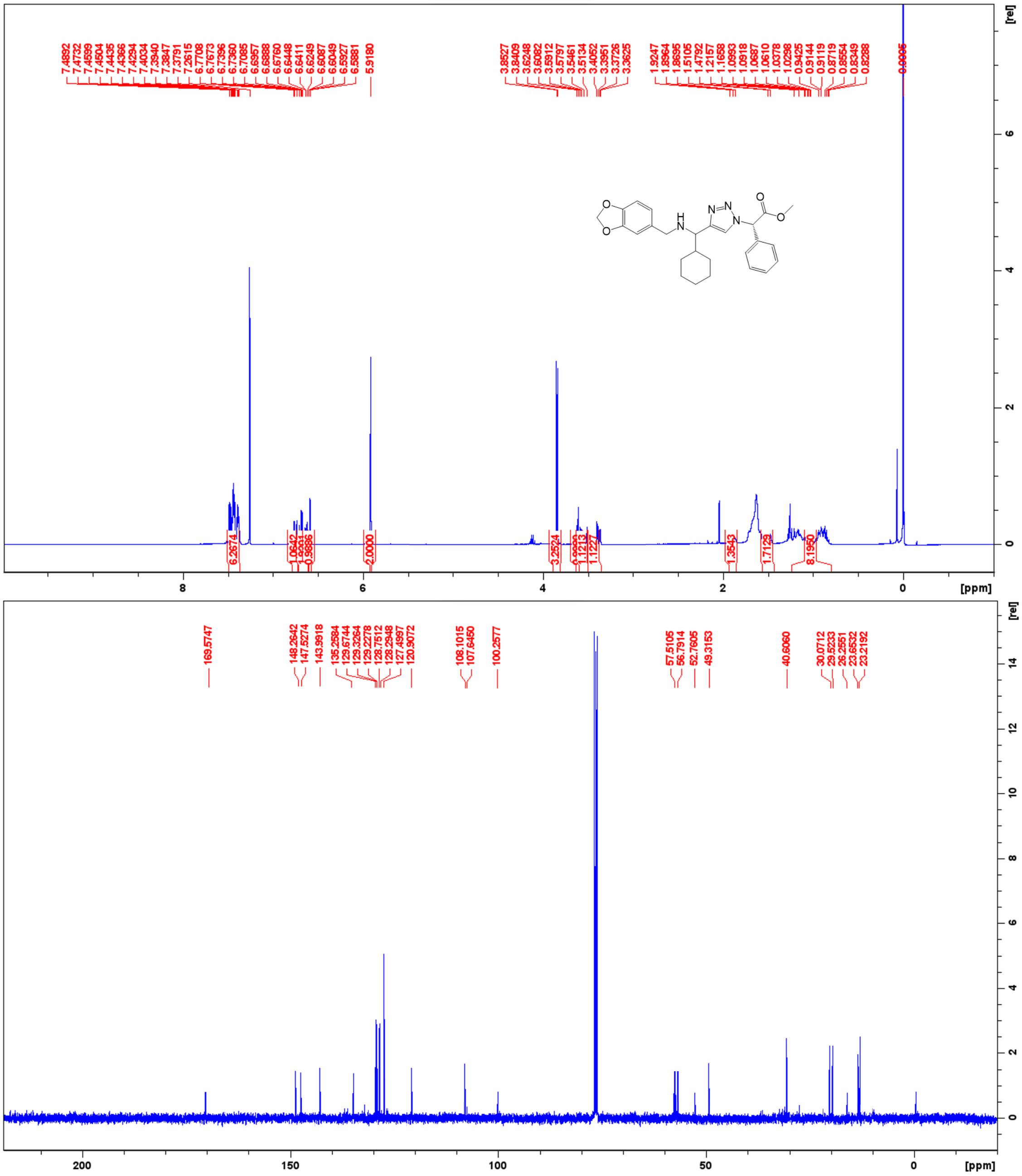

^{1}H NMR and ^{13}C NMR spectra of **Es-9**

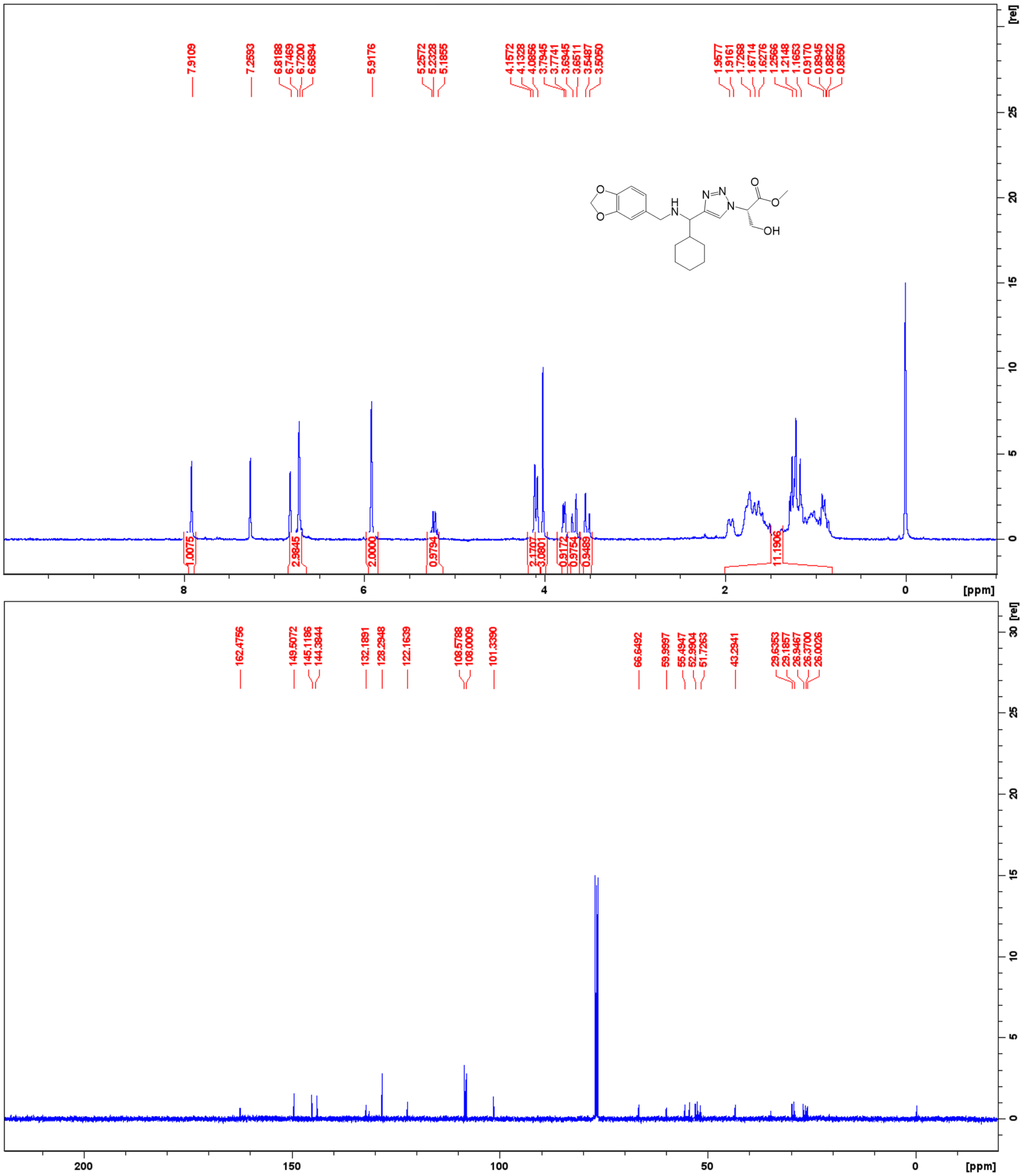

^{1}H NMR and ^{13}C NMR spectra of **Es-10**

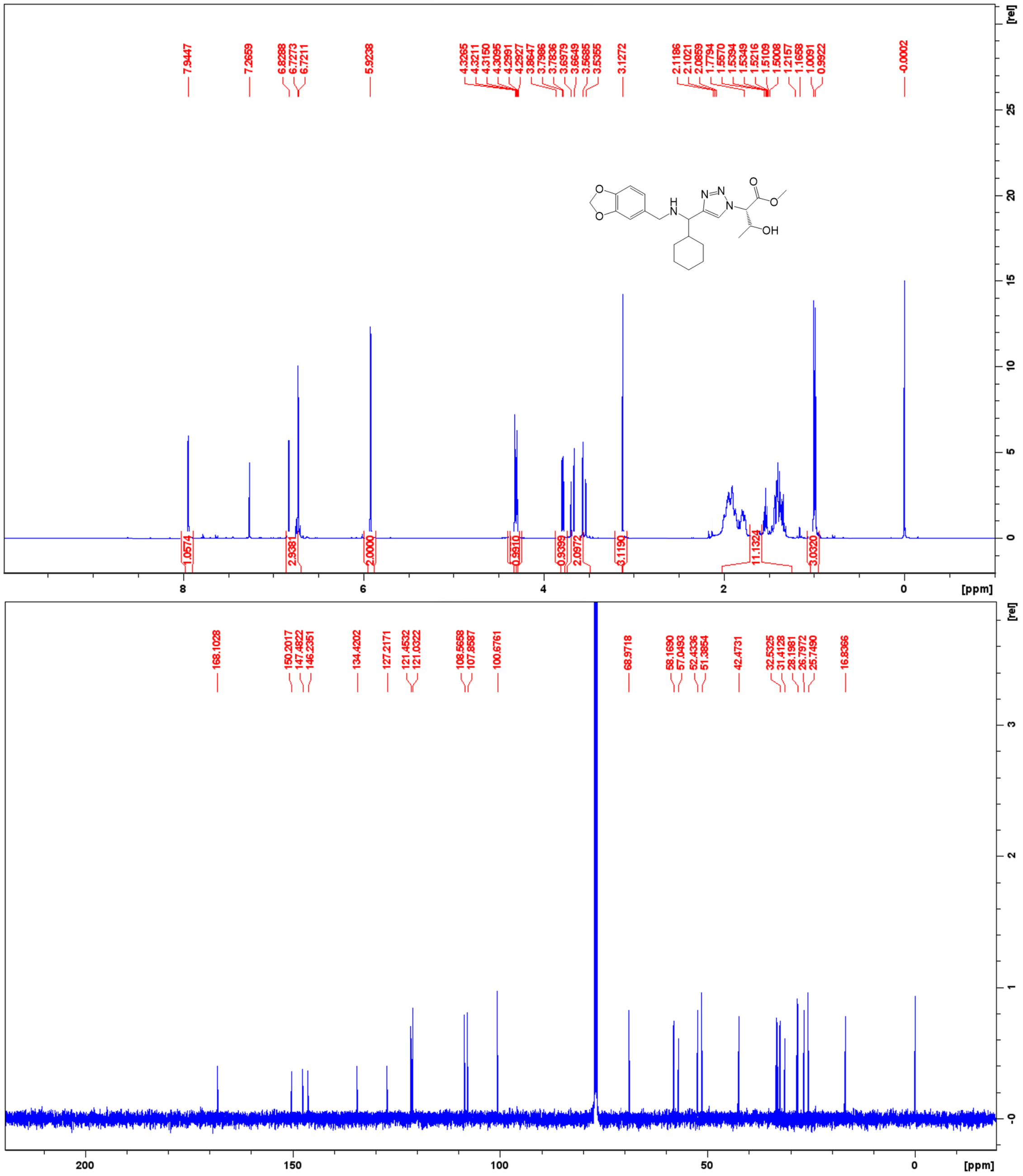

^{1}H NMR and ^{13}C NMR spectra of **Es-11**

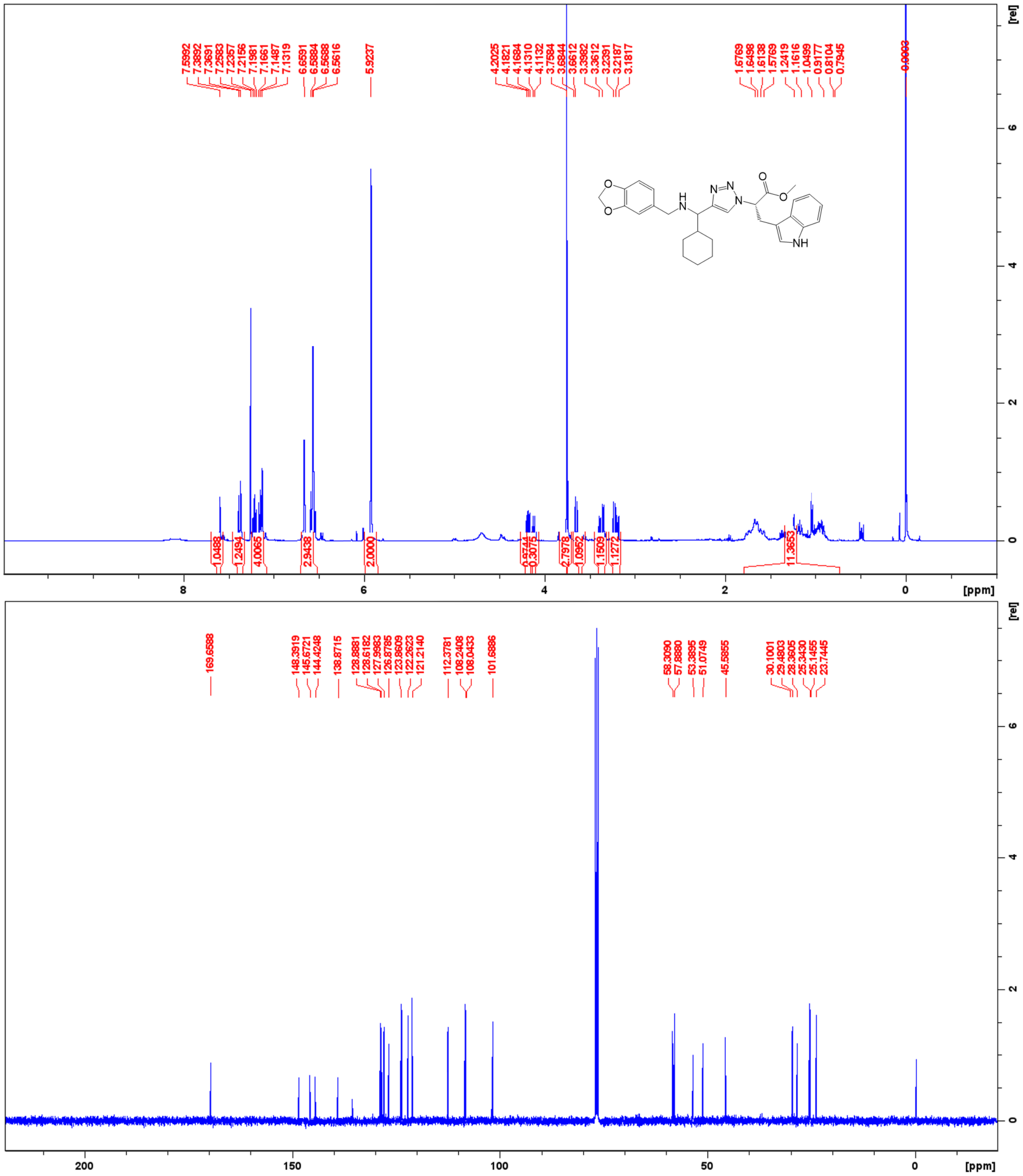

^{1}H NMR and ^{13}C NMR spectra of **Es-12**

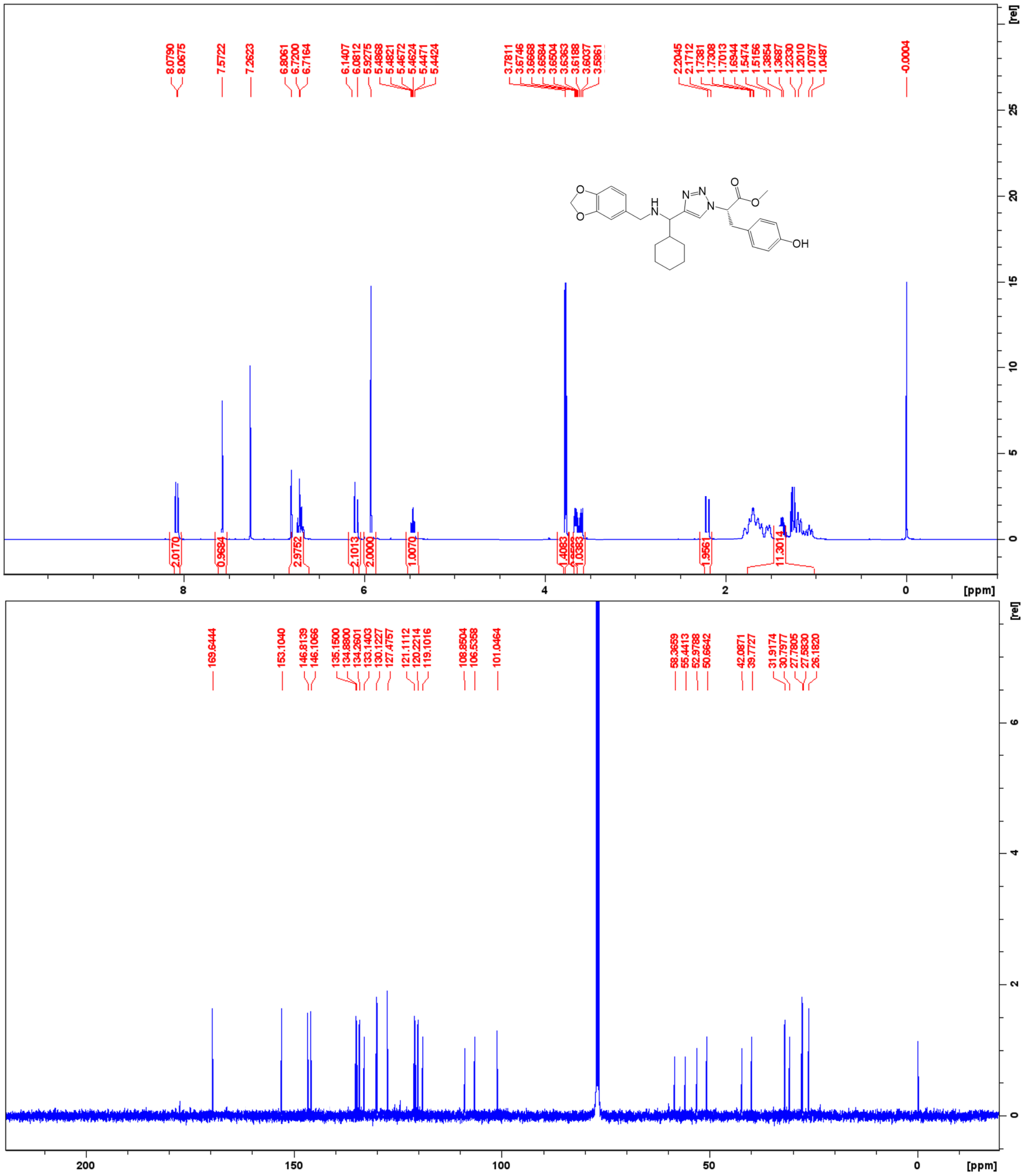

^{1}H NMR and ^{13}C NMR spectra of **Es-13**

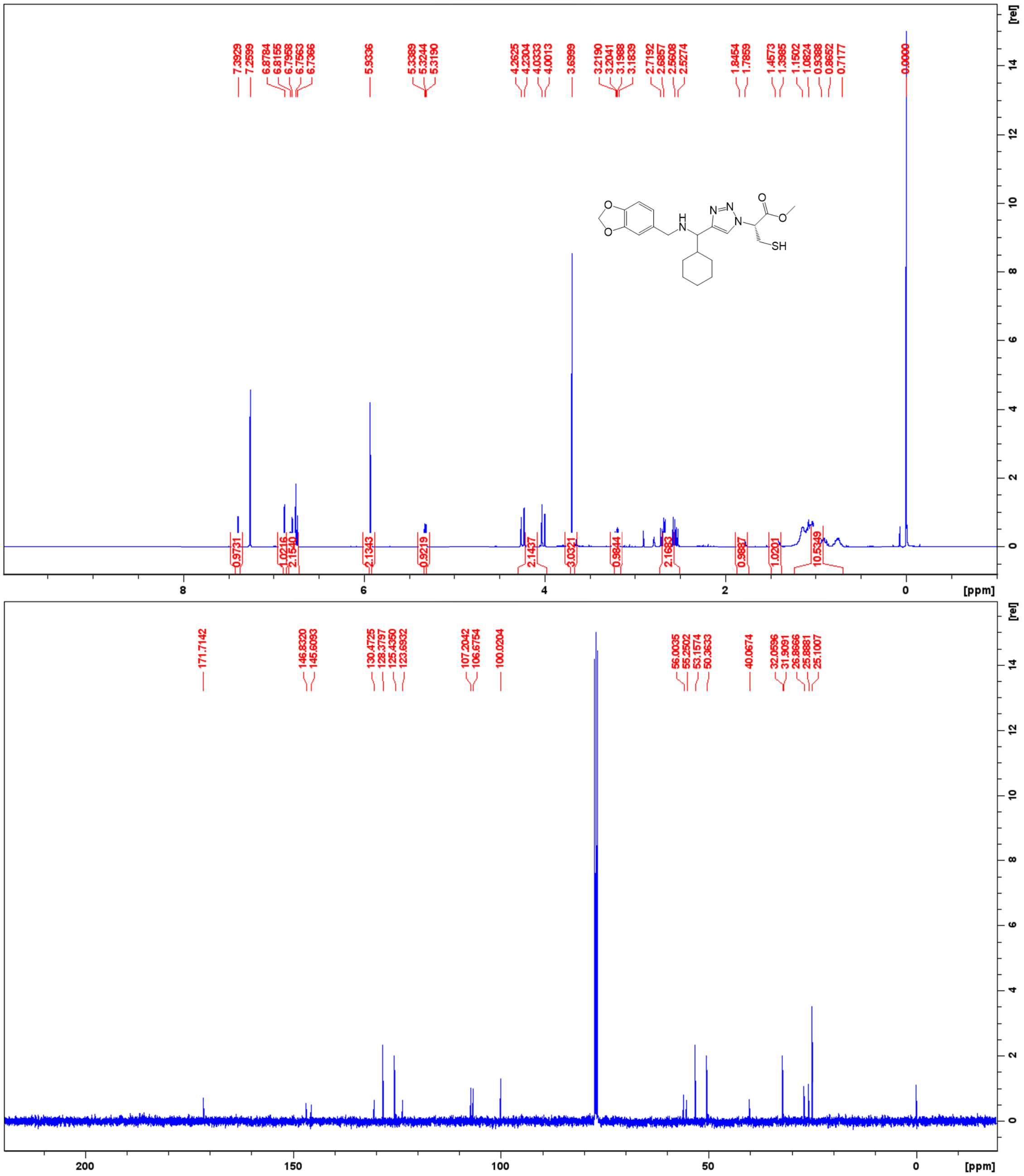

^{1}H NMR and ^{13}C NMR spectra of **Es-14**

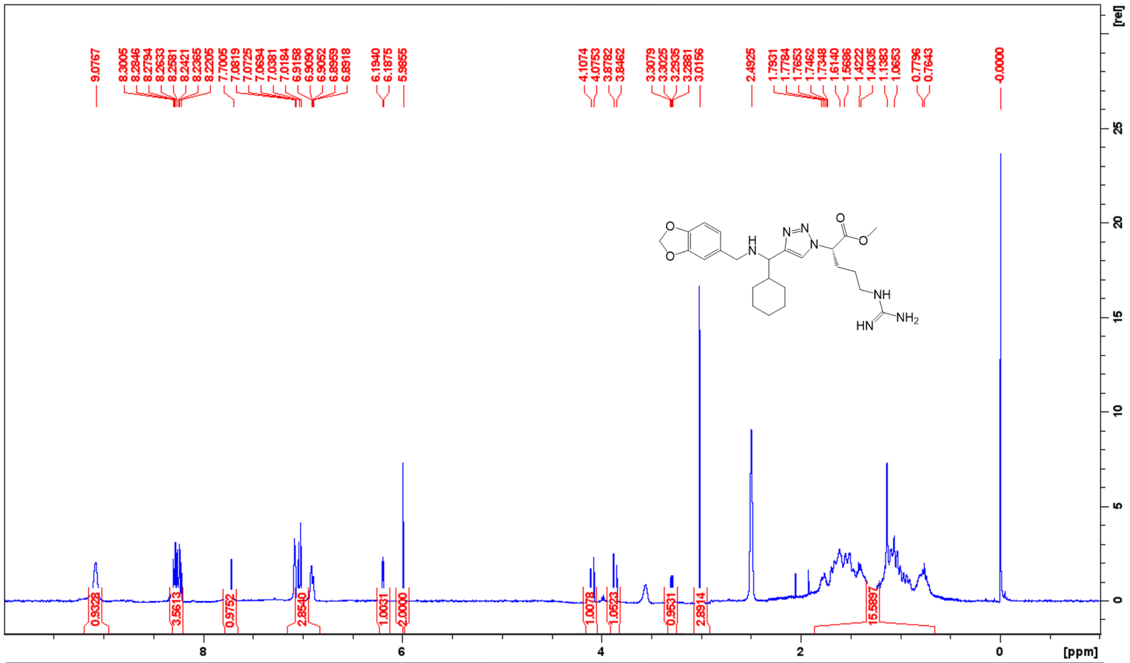

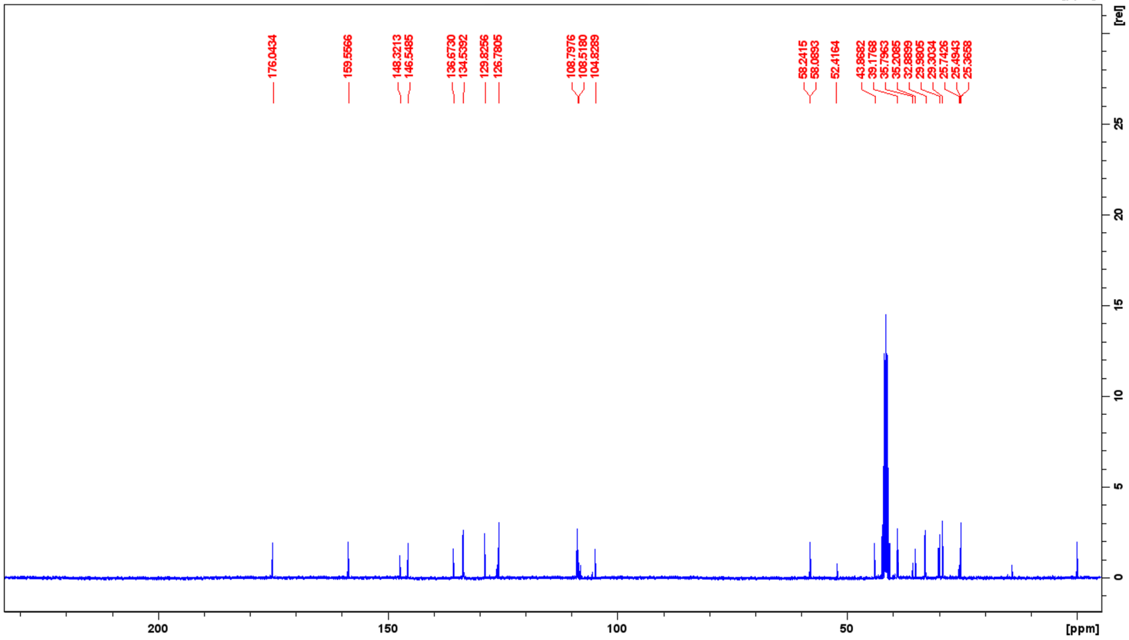

^{1}H NMR and ^{13}C NMR spectra of **Es-15**

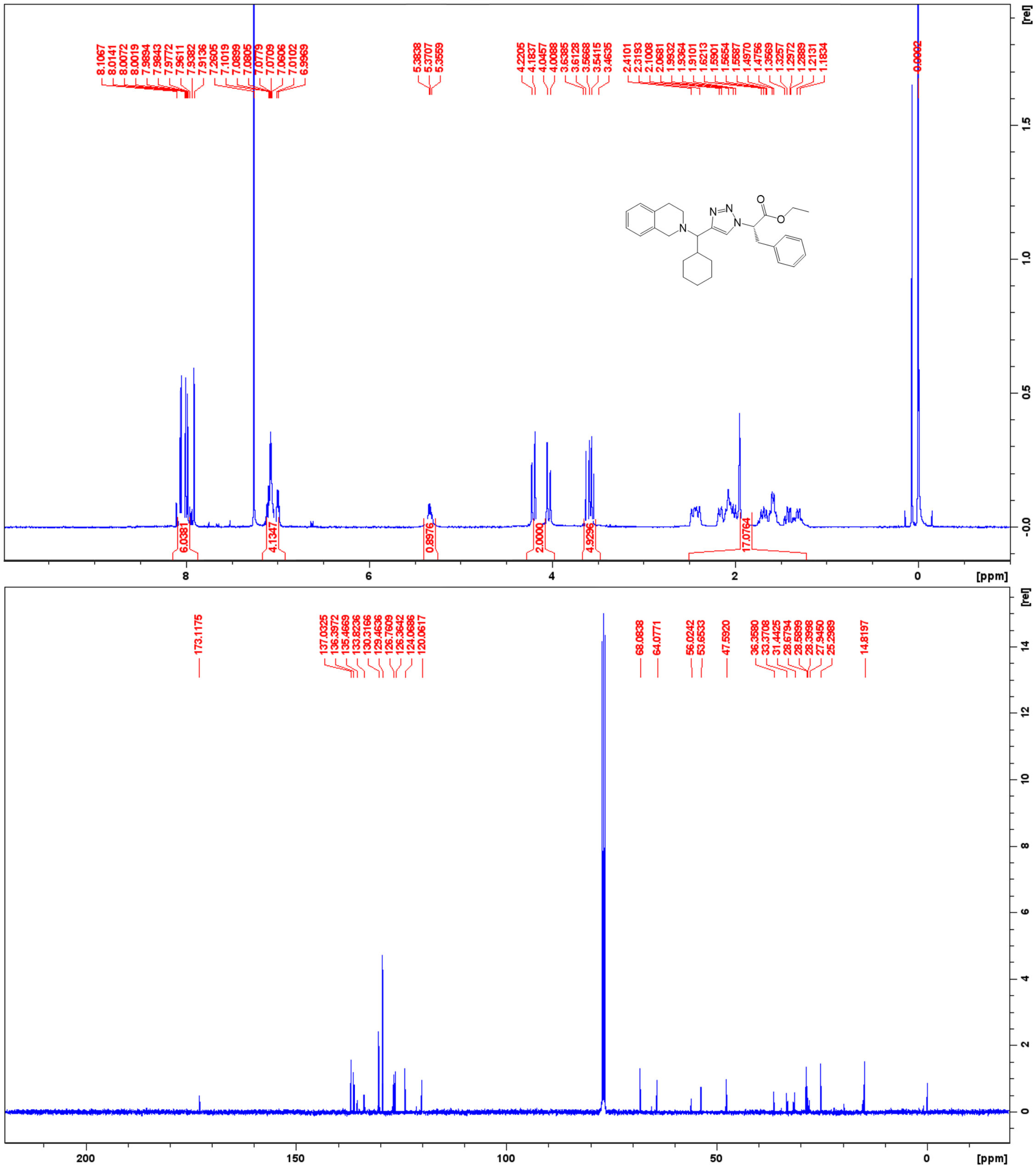

^{1}H NMR and ^{13}C NMR spectra of **Es-16**

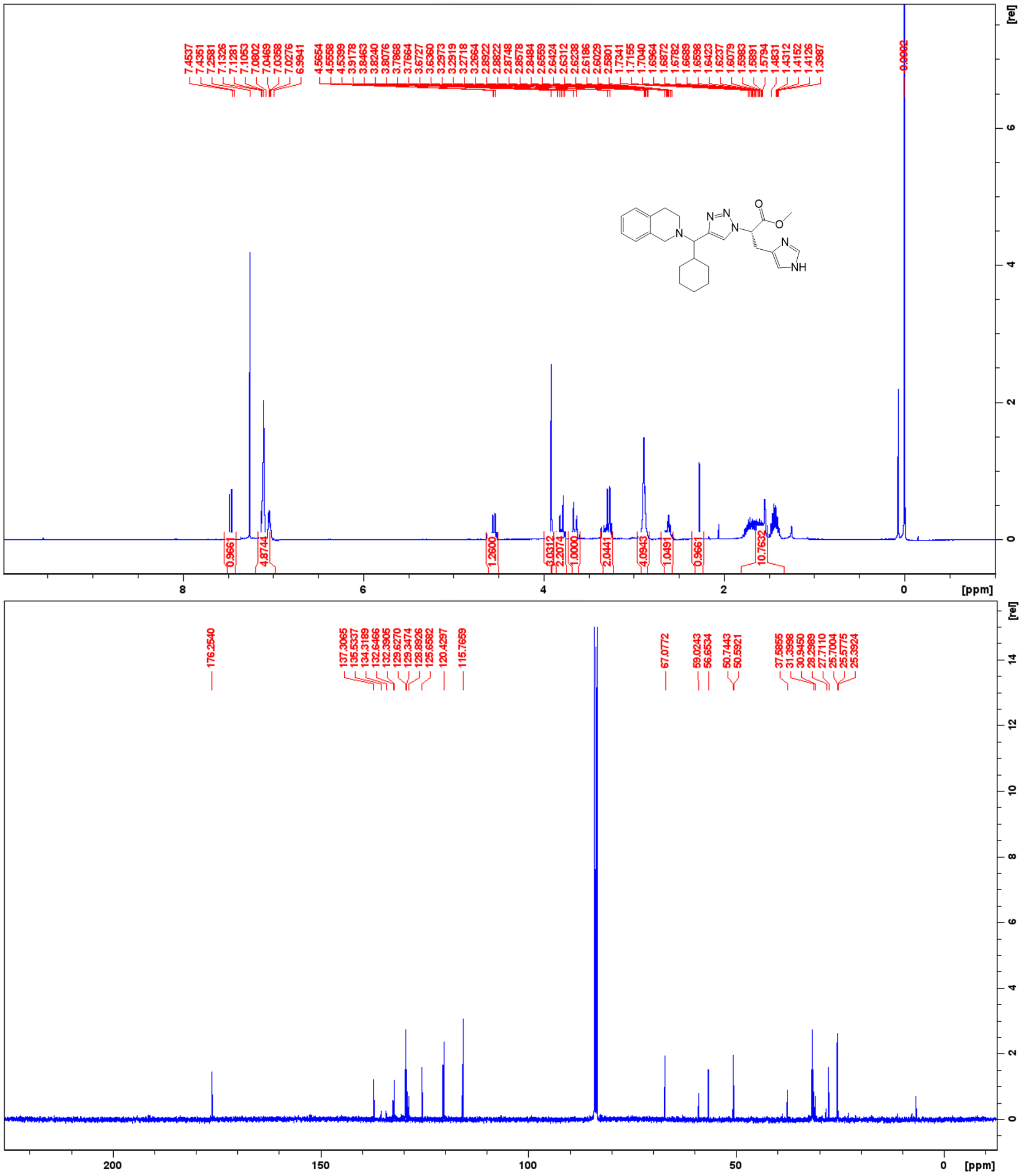

^{1}H NMR and ^{13}C NMR spectra of **Es-17**

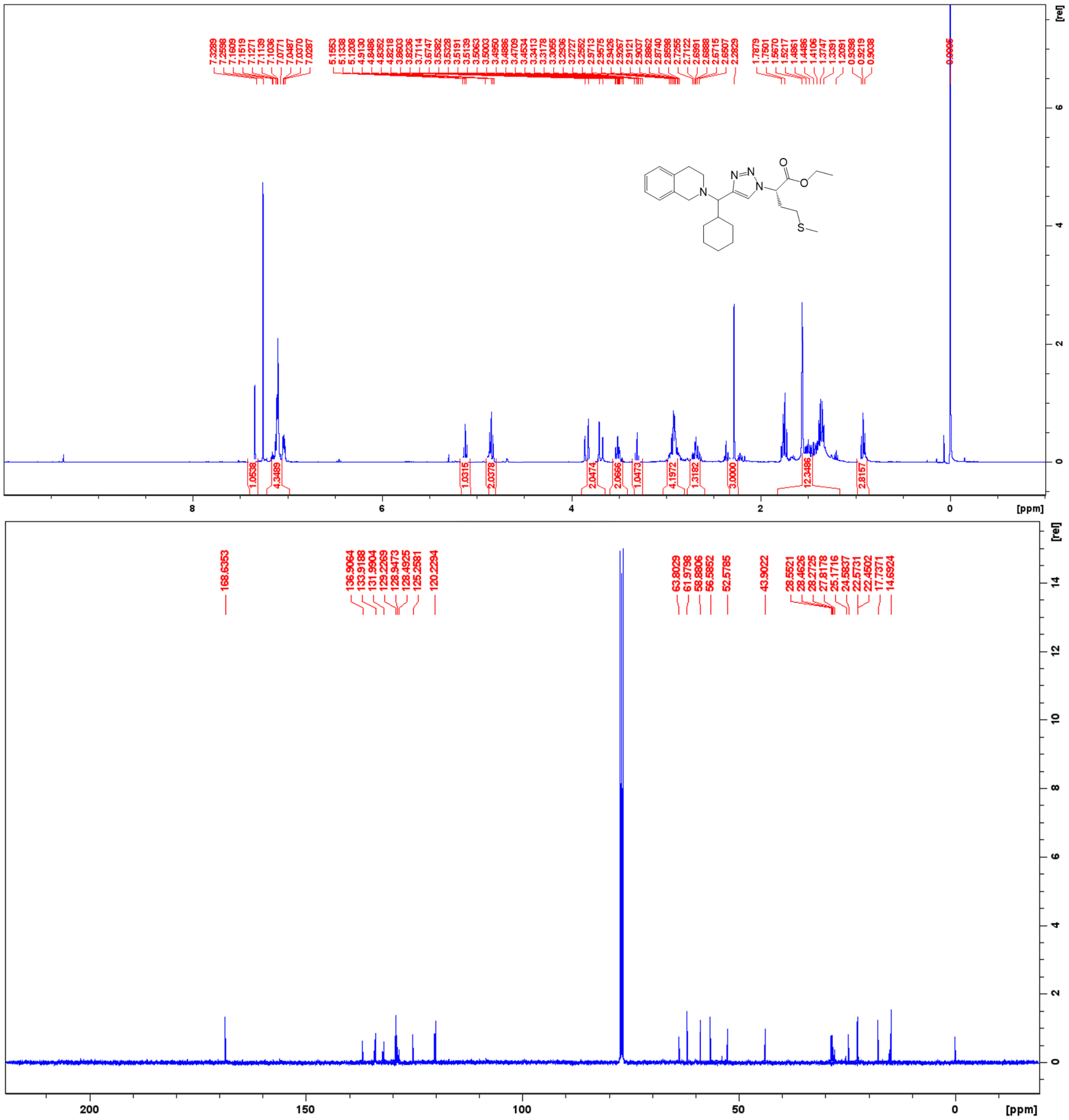

^{1}H NMR and ^{13}C NMR spectra of **Es-18**

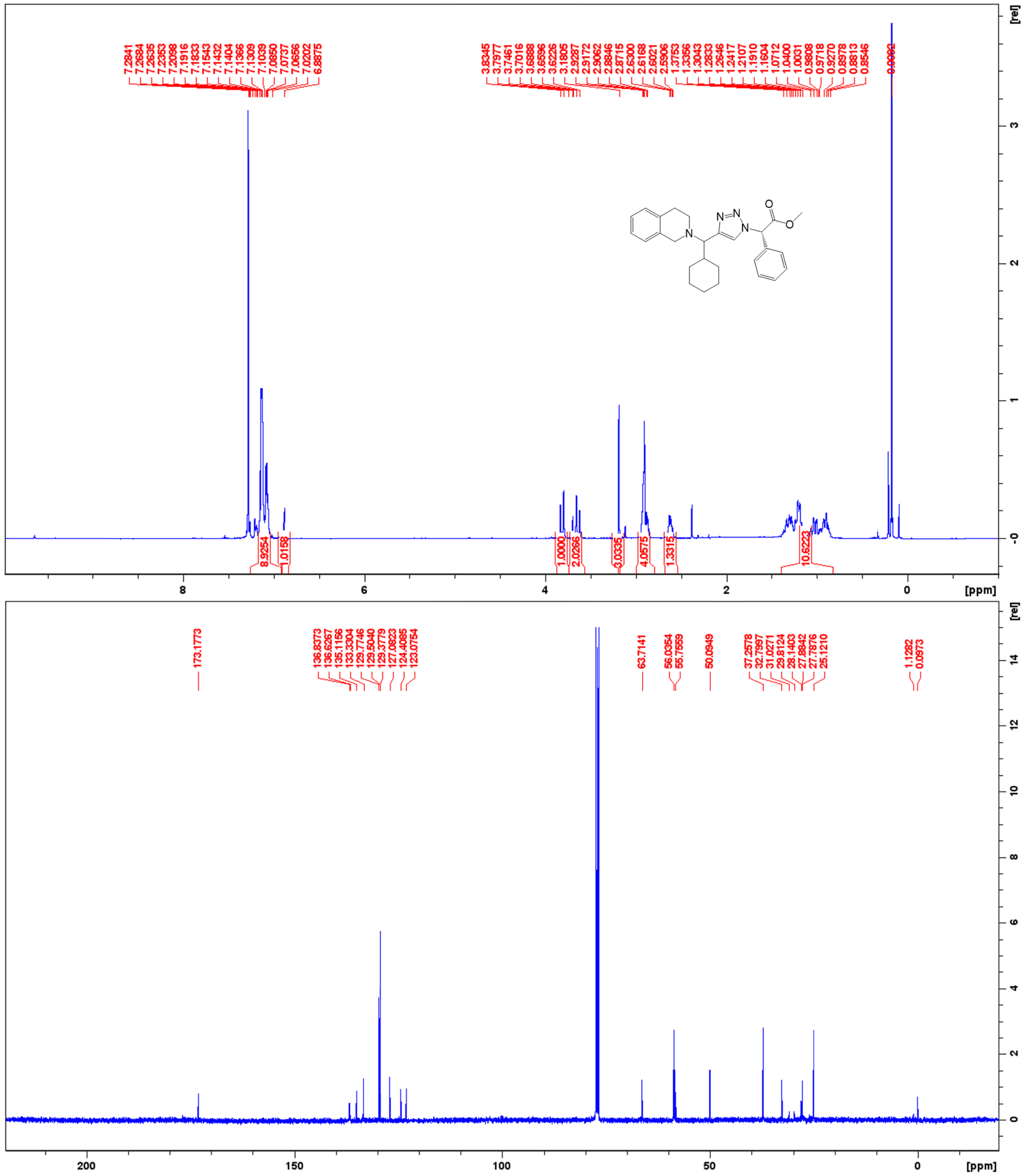

^{1}H NMR and ^{13}C NMR spectra of **Es-19**

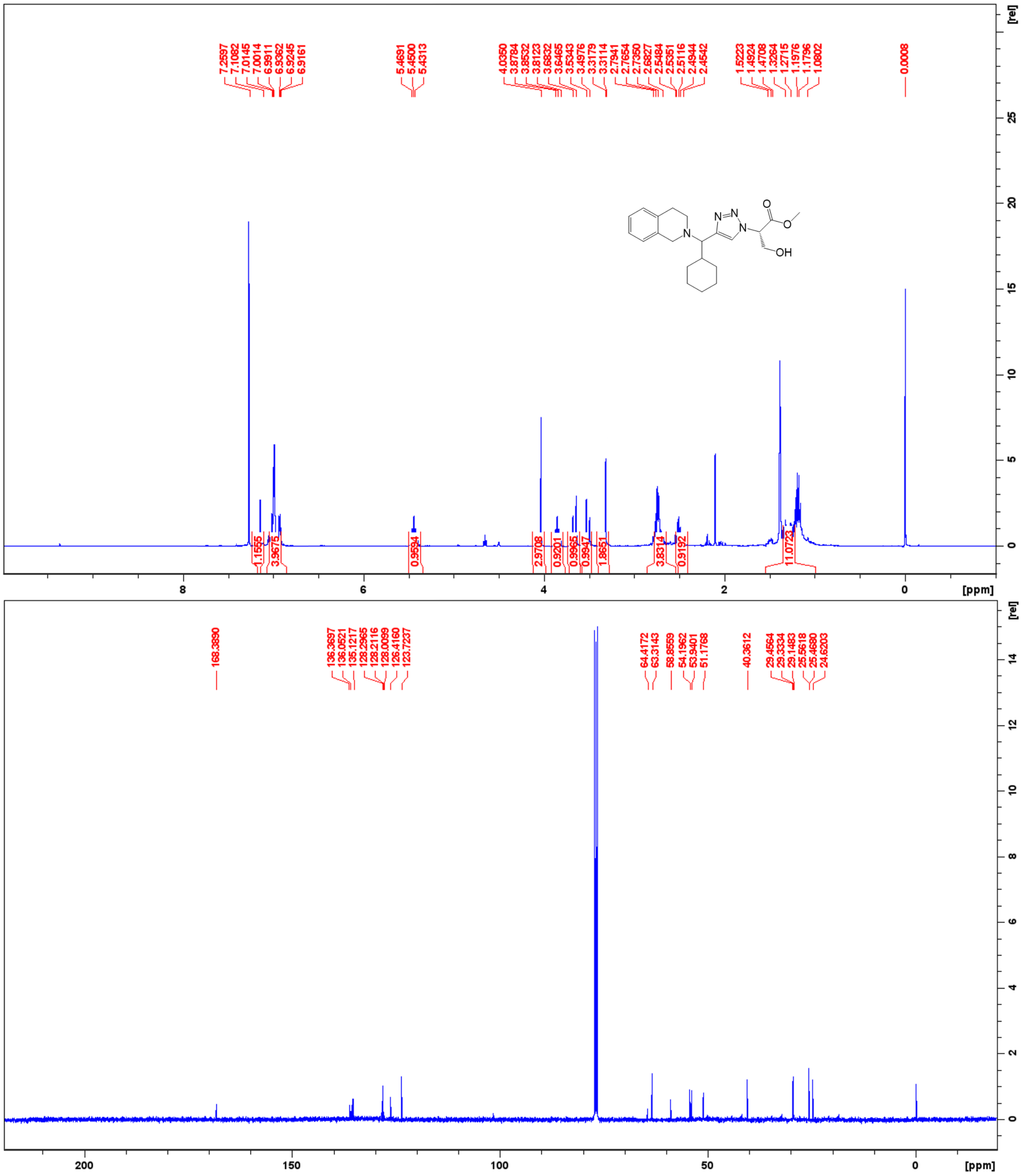

^{1}H NMR and ^{13}C NMR spectra of **Es-20**

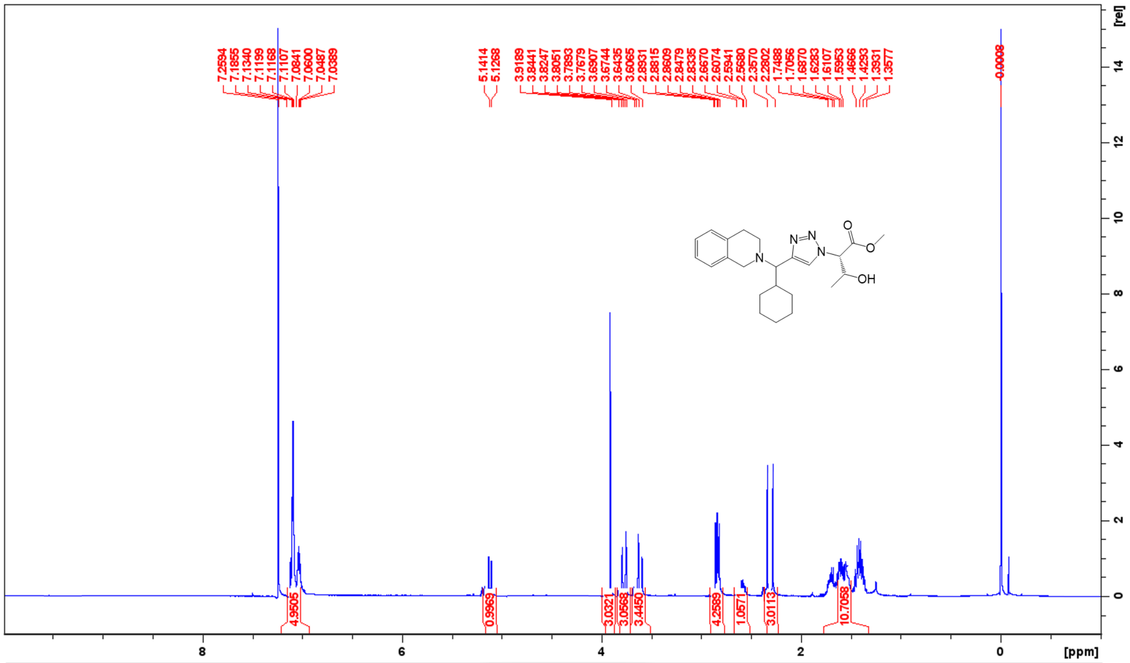

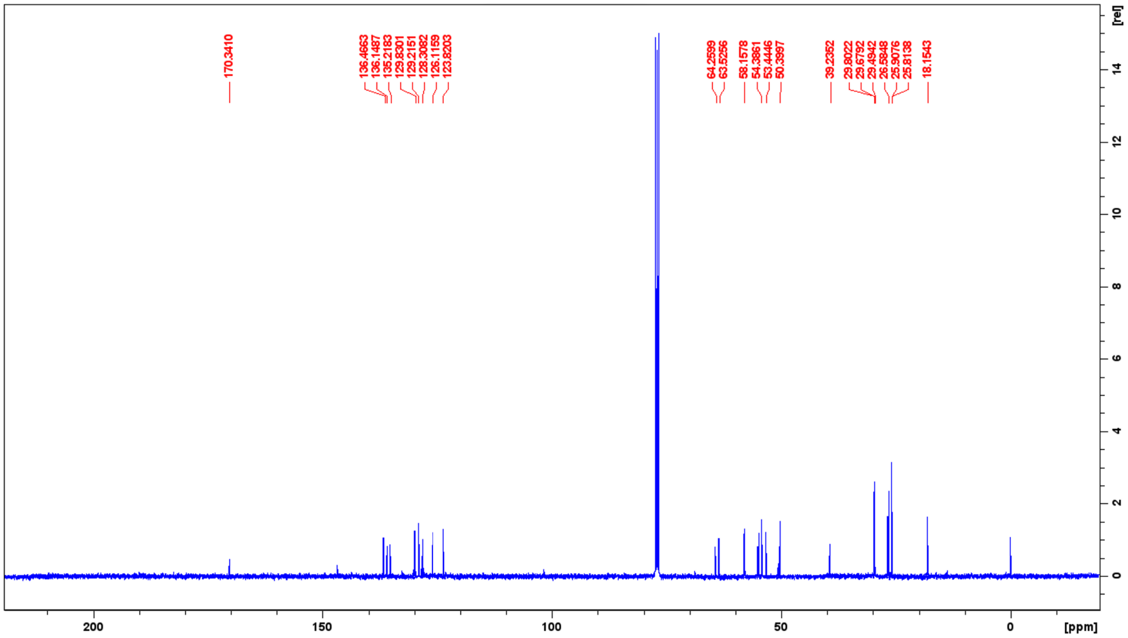

^{1}H NMR and ^{13}C NMR spectra of **Es-21**

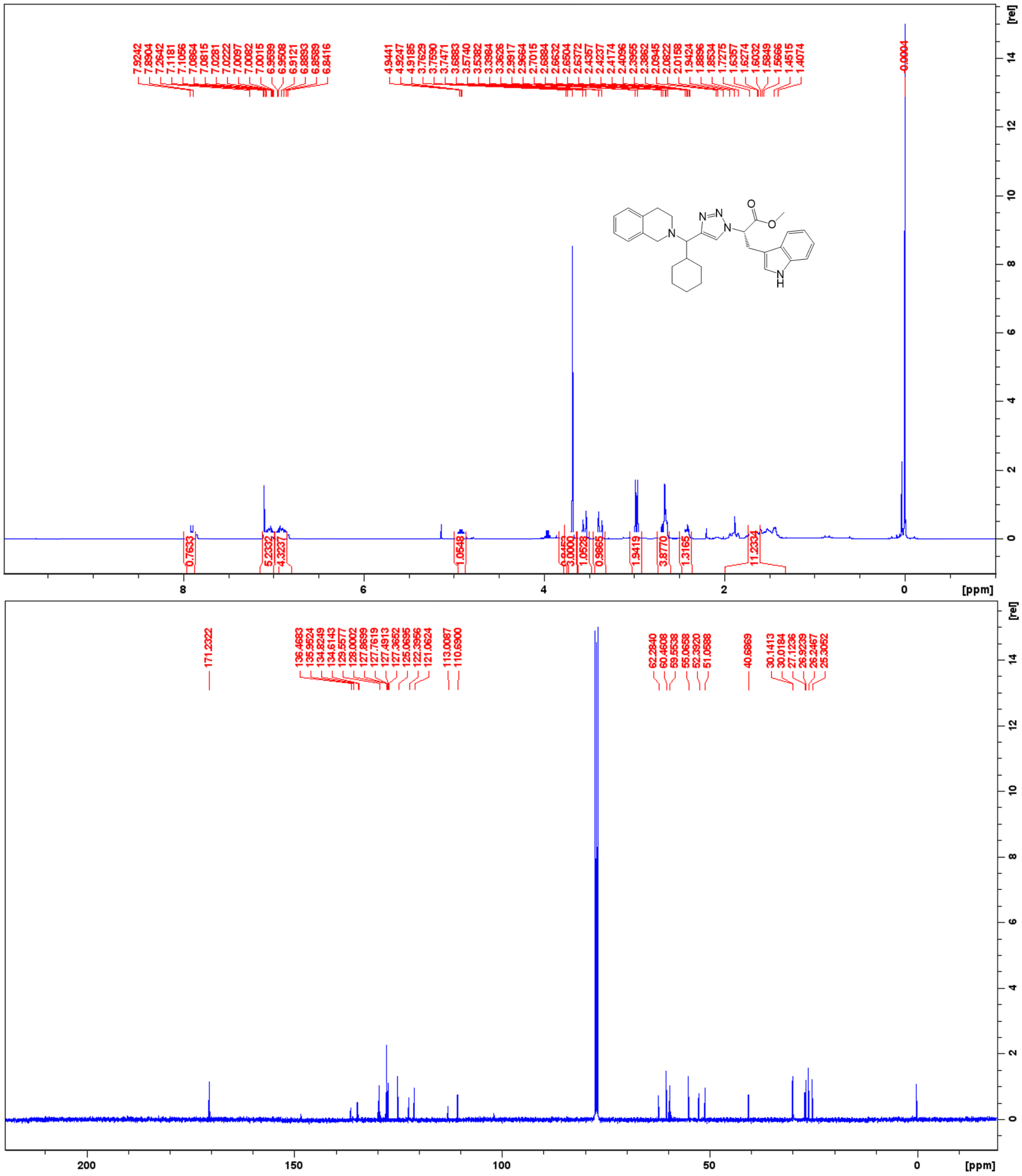

^{1}H NMR and ^{13}C NMR spectra of **Es-22**

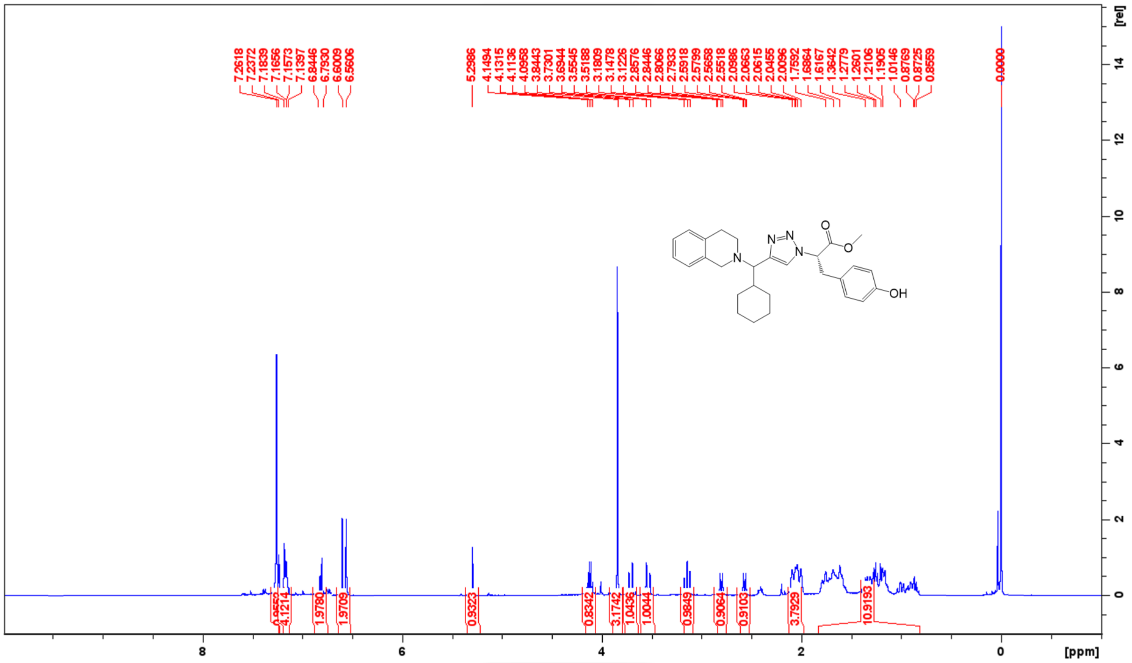

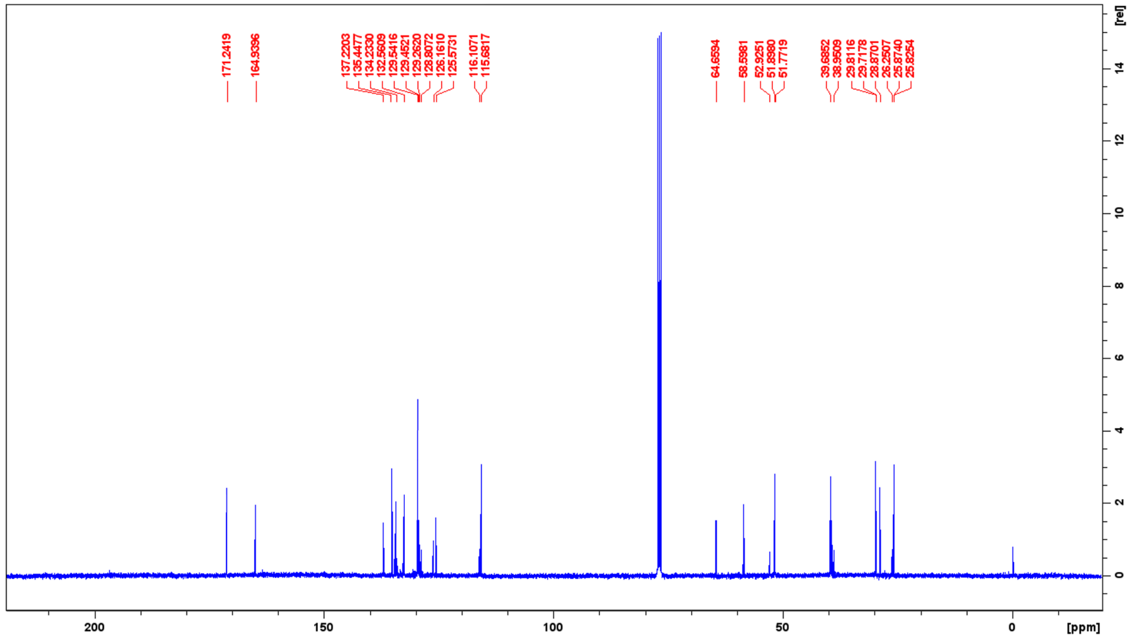

^{1}H NMR and ^{13}C NMR spectra of **Es-23**

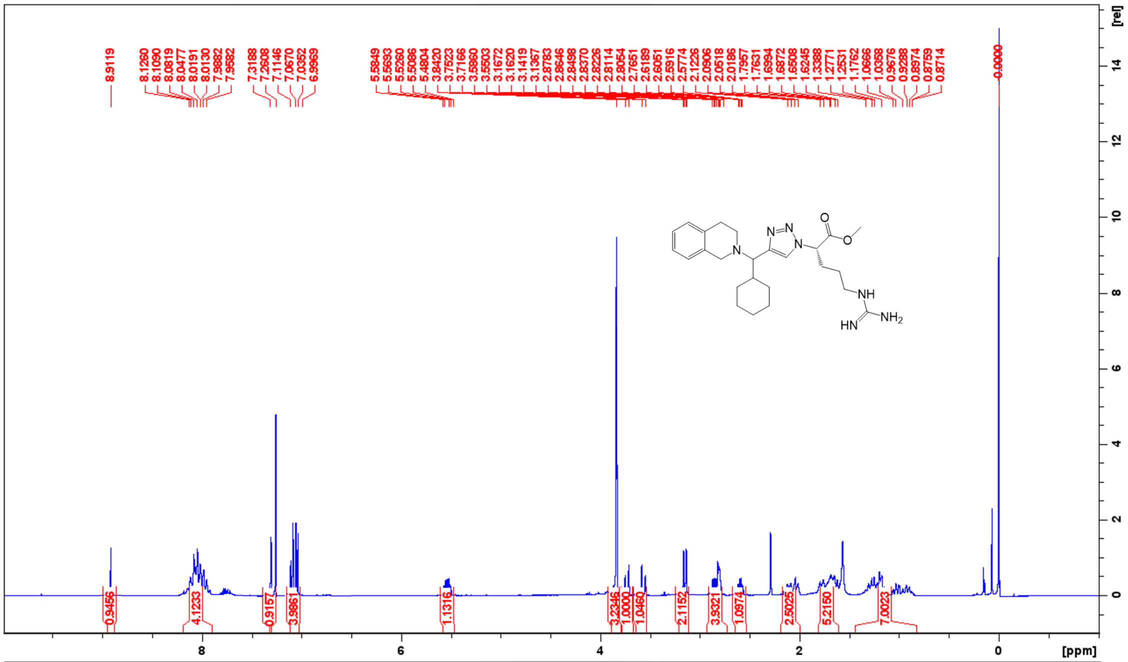

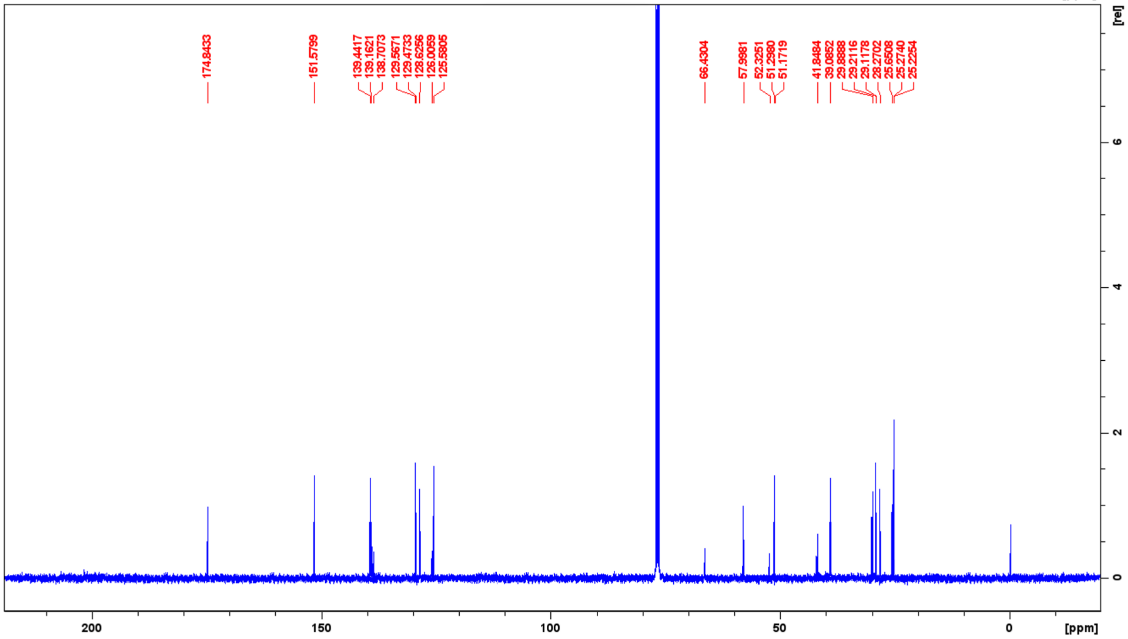

^{1}H NMR and ^{13}C NMR spectra of **Es-24**

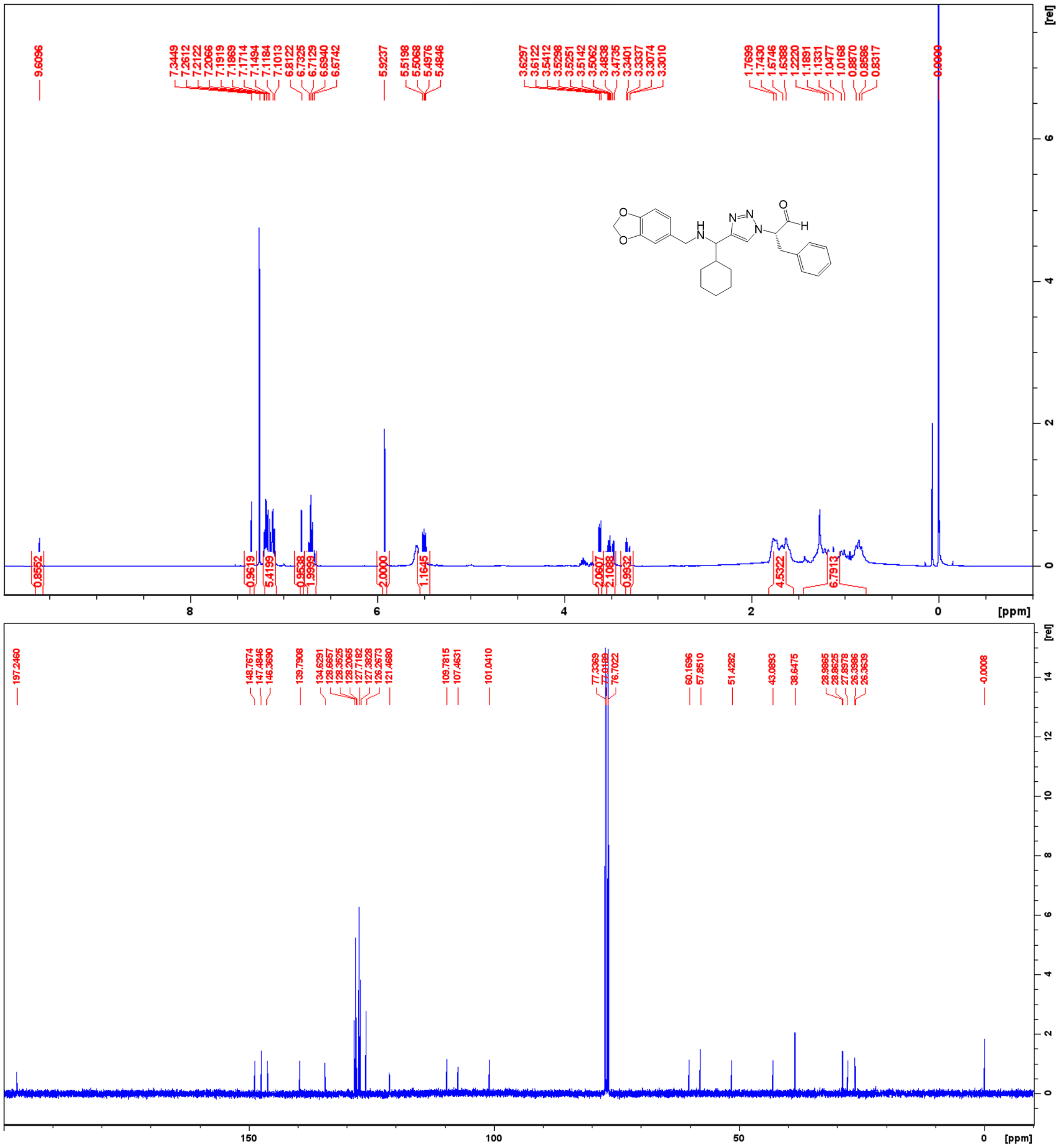

^{1}H NMR and ^{13}C NMR spectra of **Ald-1**

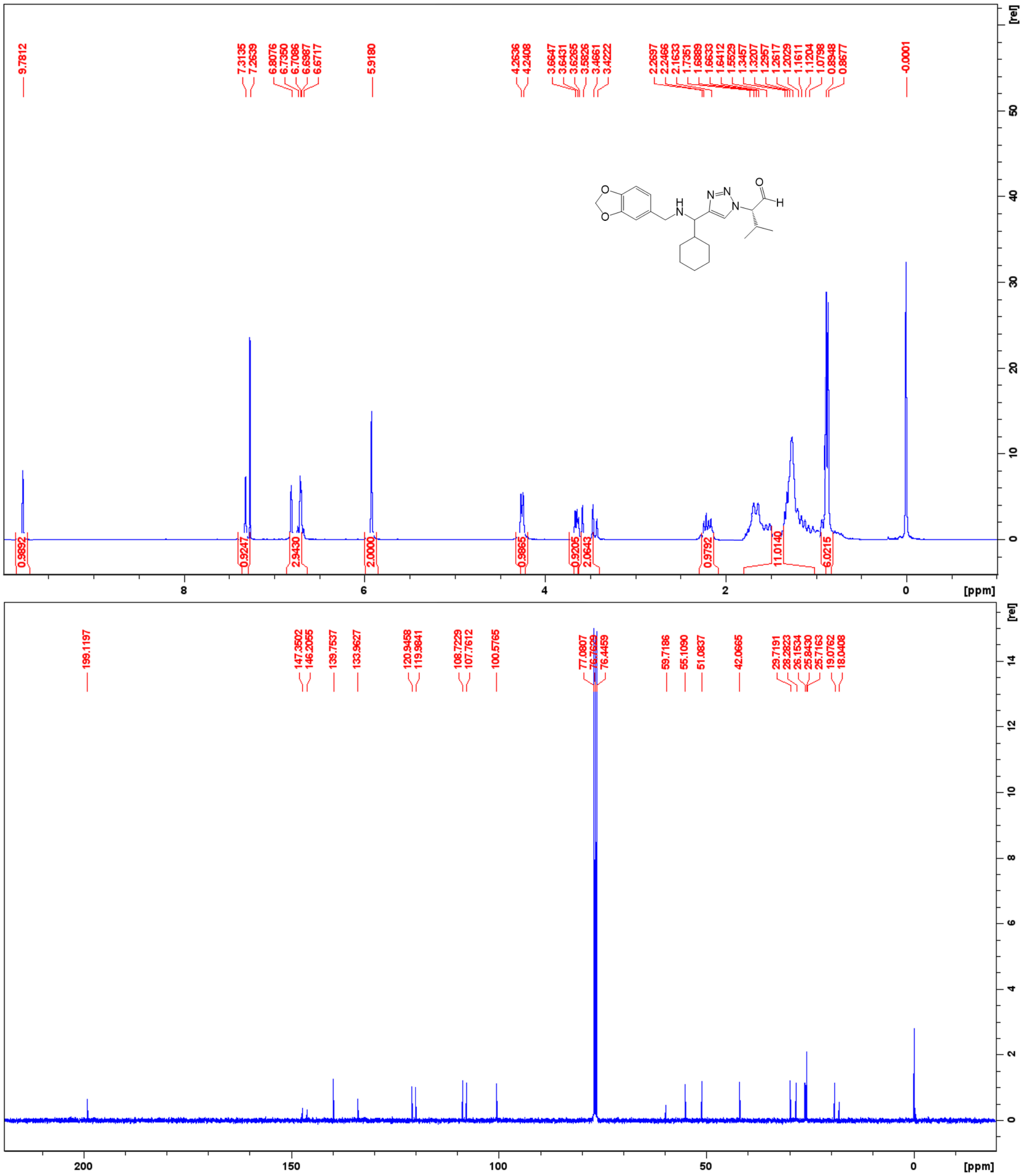

^{1}H NMR and ^{13}C NMR spectra of **Ald-2**

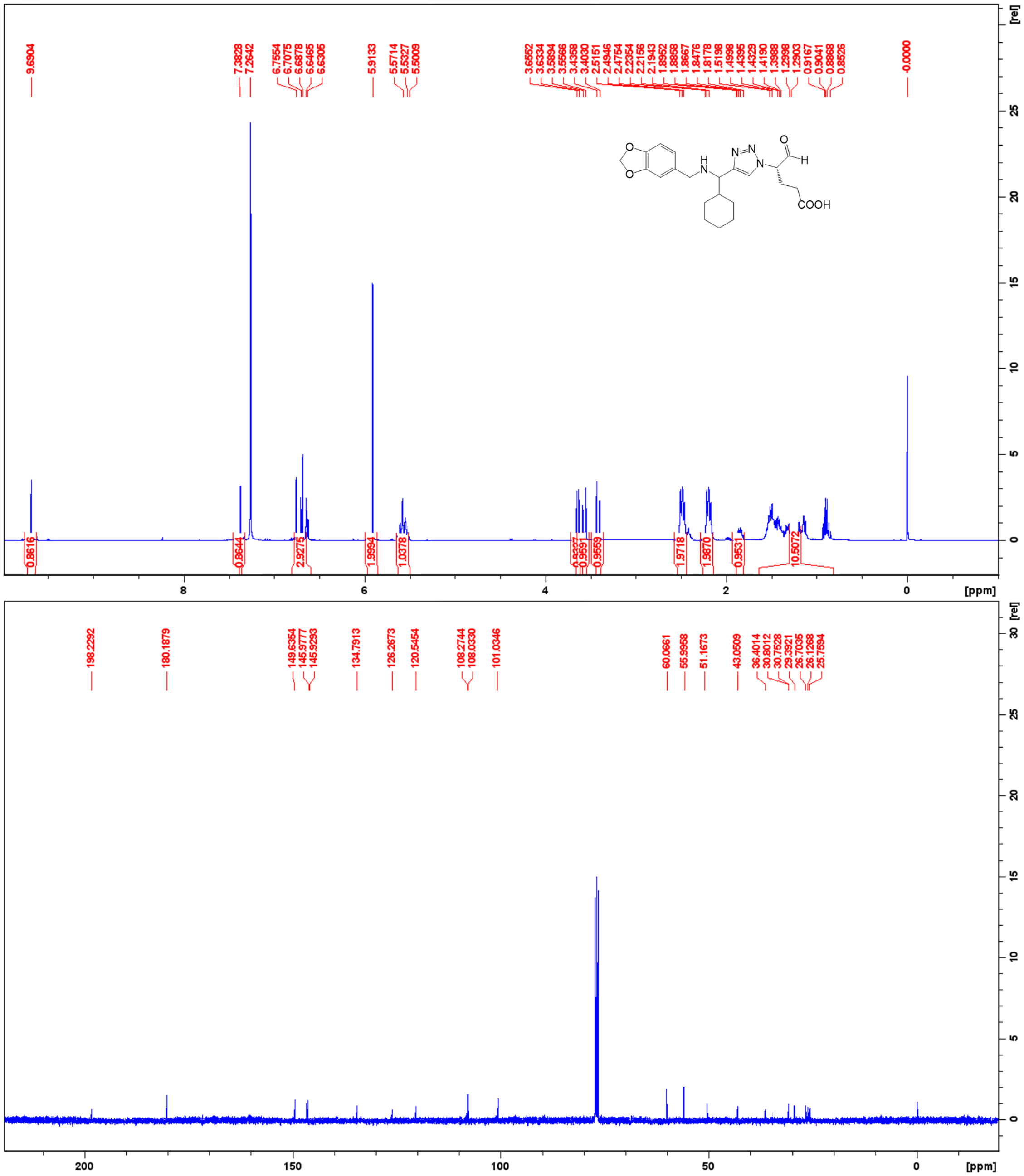

^{1}H NMR and ^{13}C NMR spectra of **Ald-3**

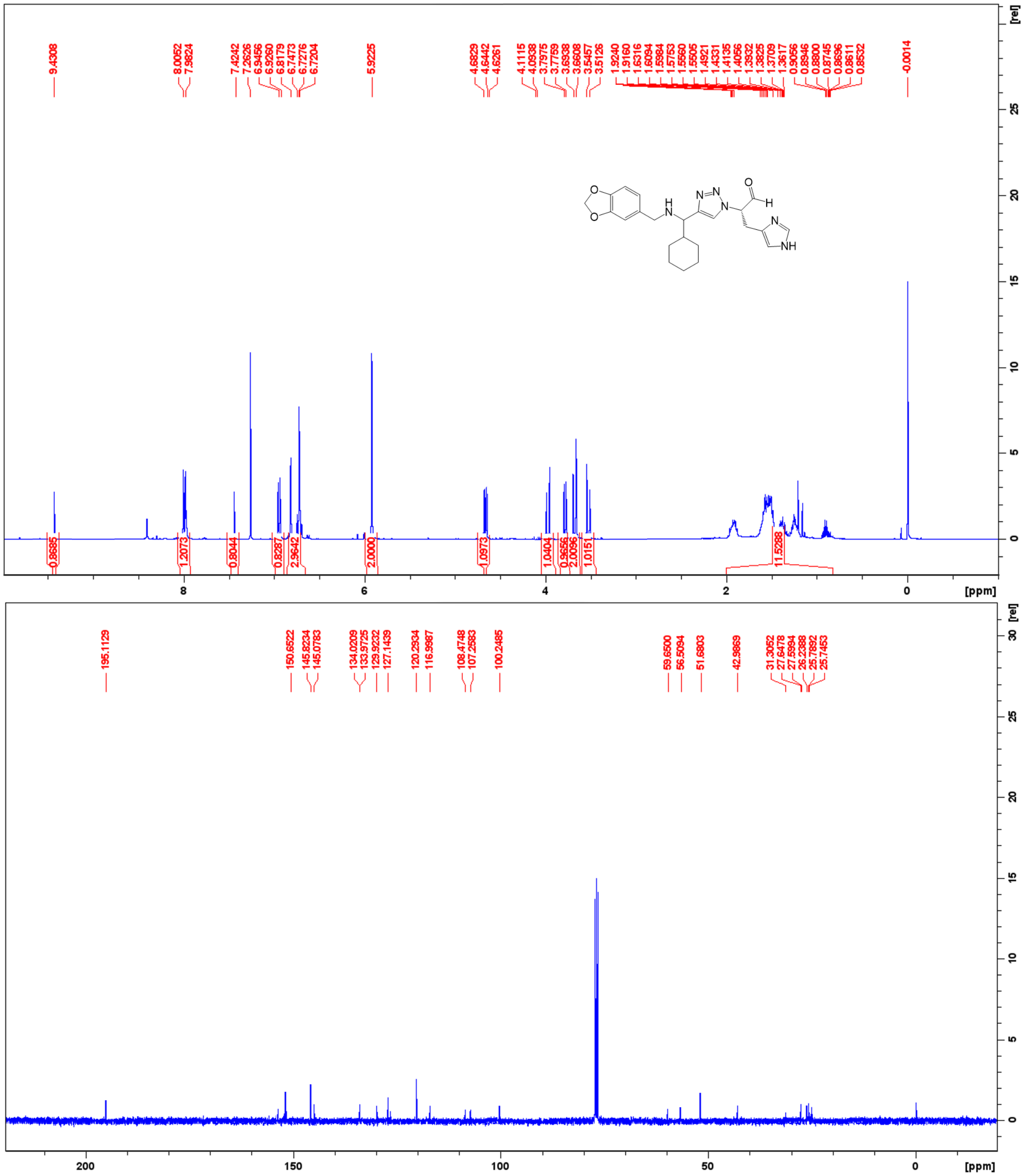

^{1}H NMR and ^{13}C NMR spectra of **Ald-4**

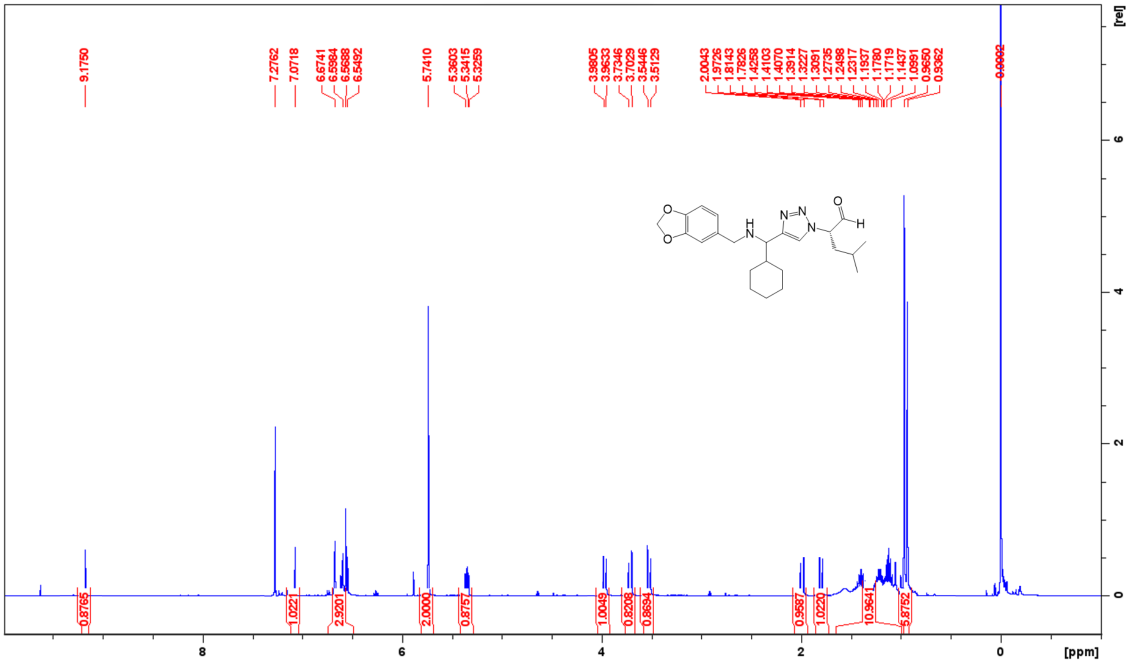

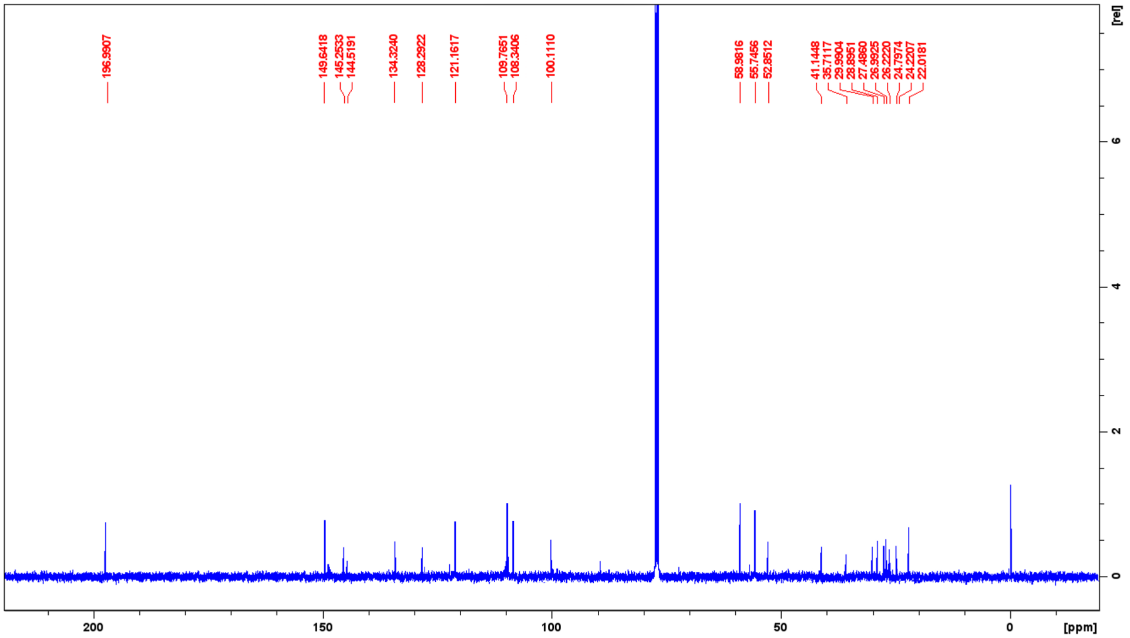

^{1}H NMR and ^{13}C NMR spectra of **Ald-5**

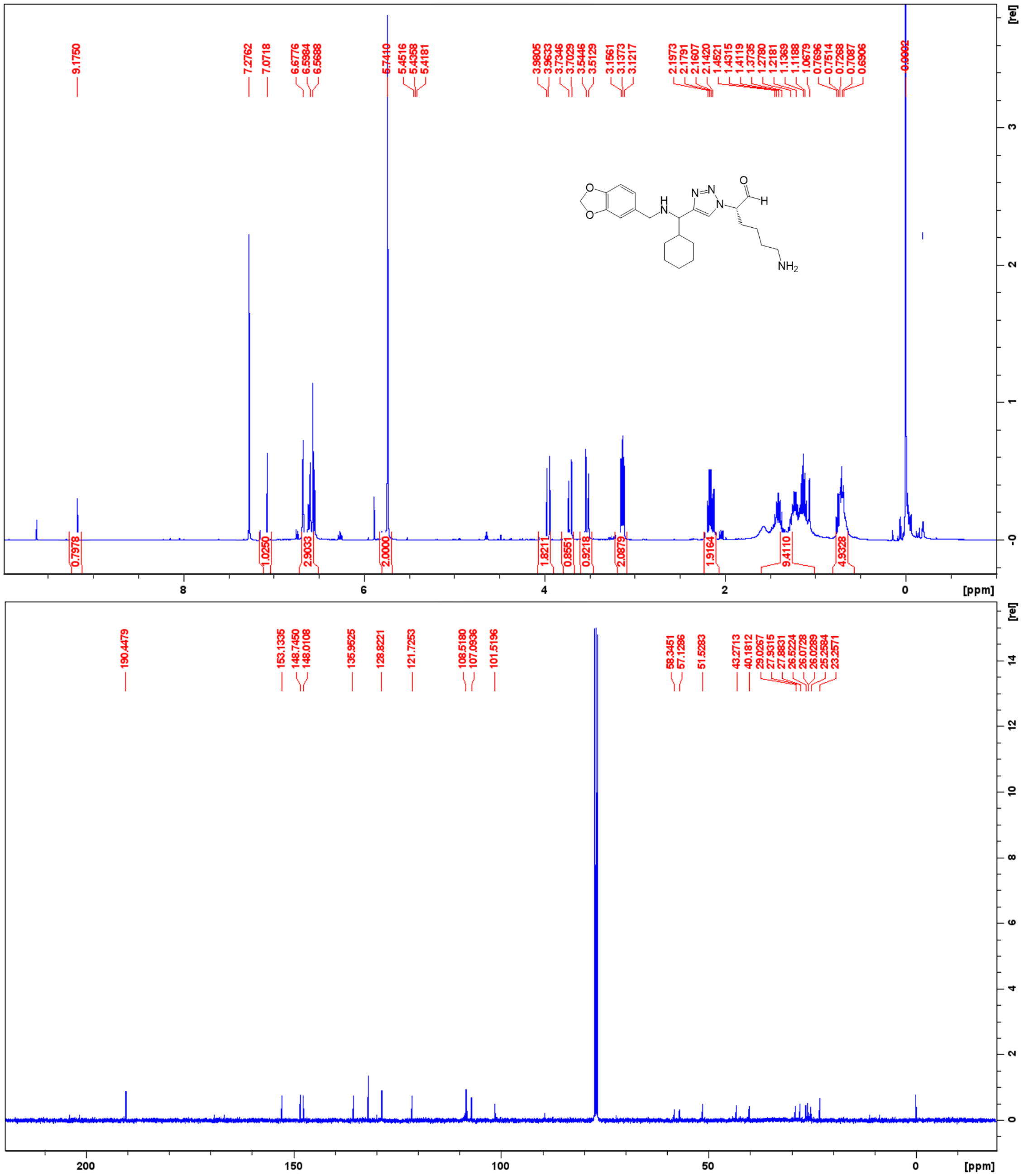

^{1}H NMR and ^{13}C NMR spectra of **Ald-6**

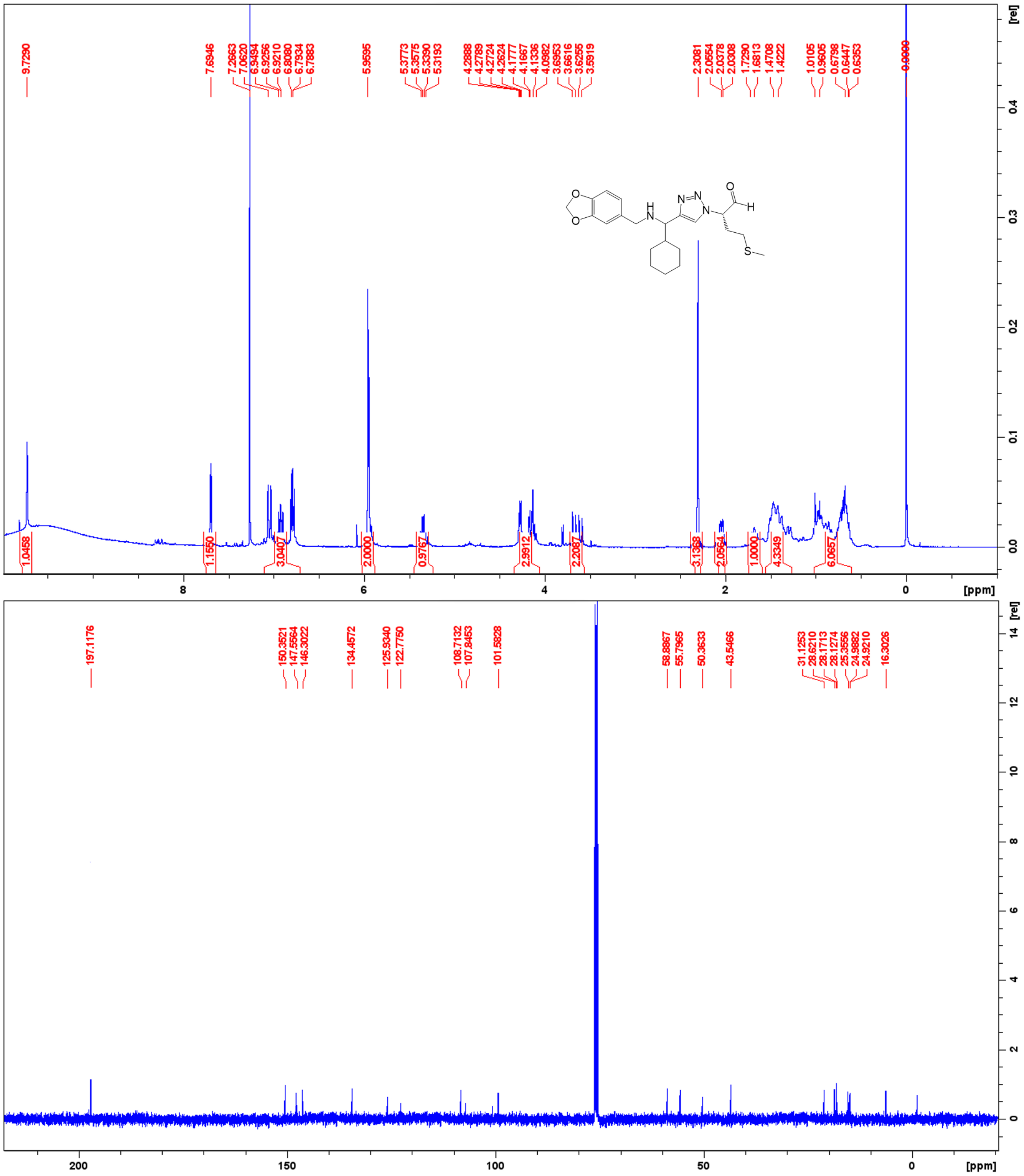

^{1}H NMR and ^{13}C NMR spectra of **Ald-7**

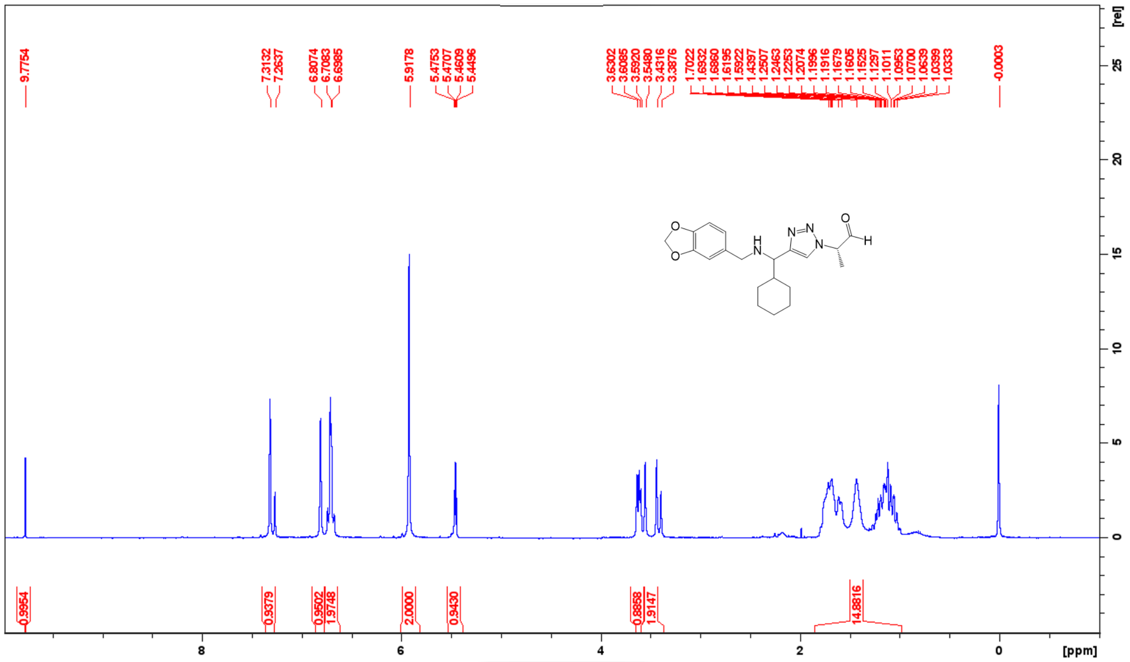

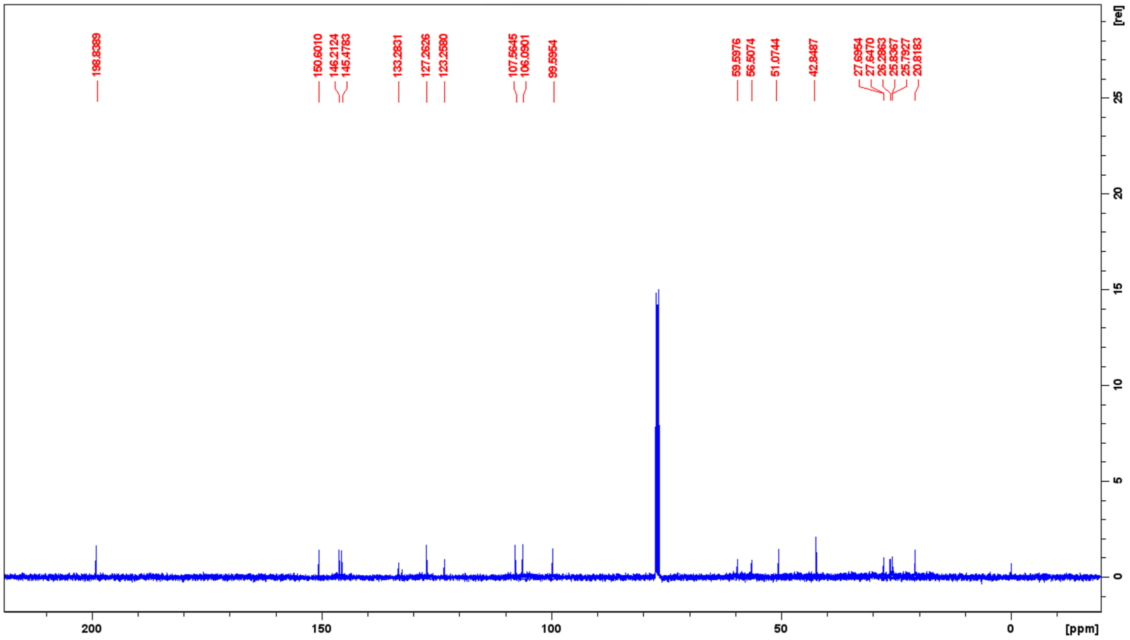

^{1}H NMR and ^{13}C NMR spectra of **Ald-8**

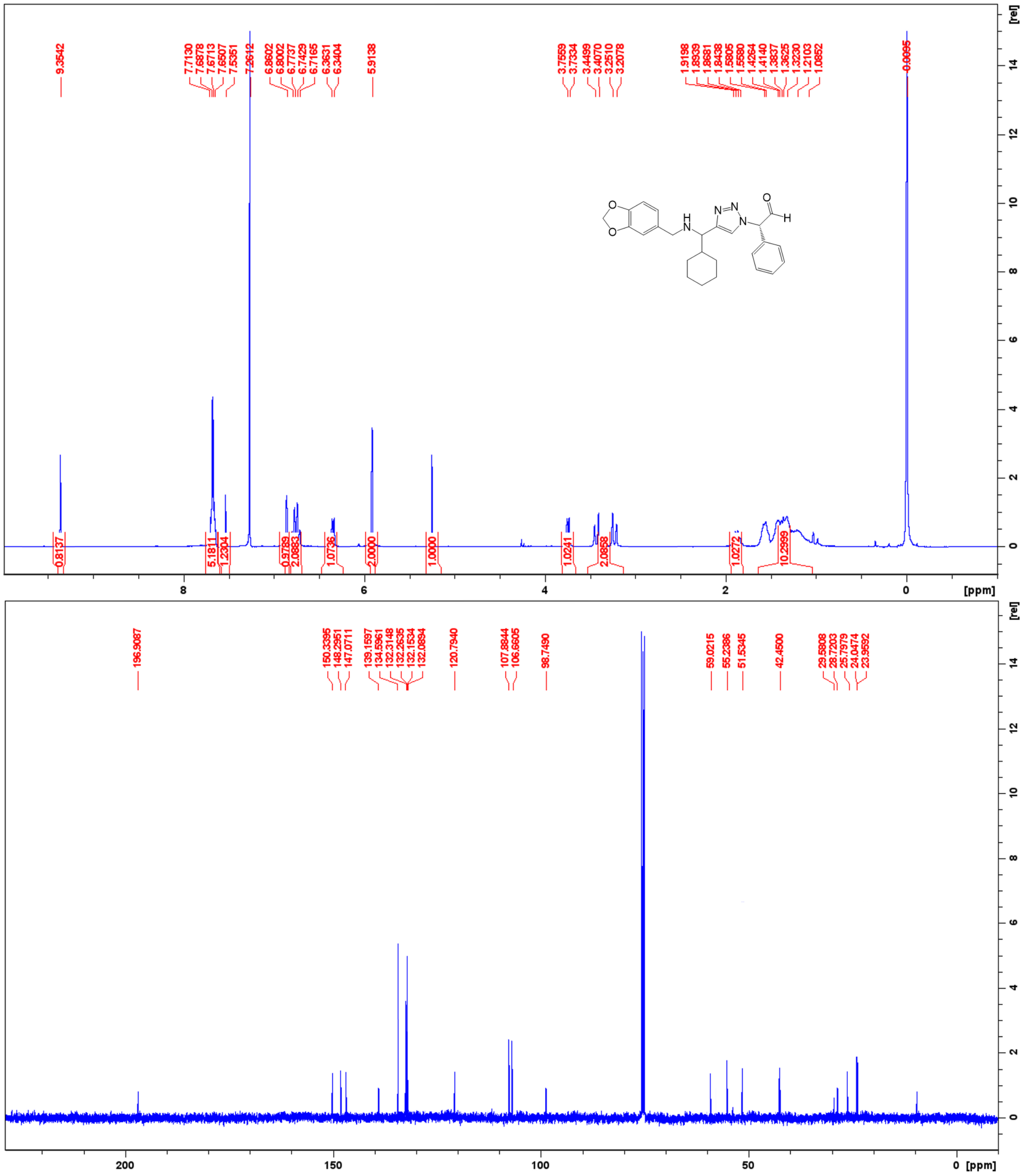

^{1}H NMR and ^{13}C NMR spectra of **Ald-9**

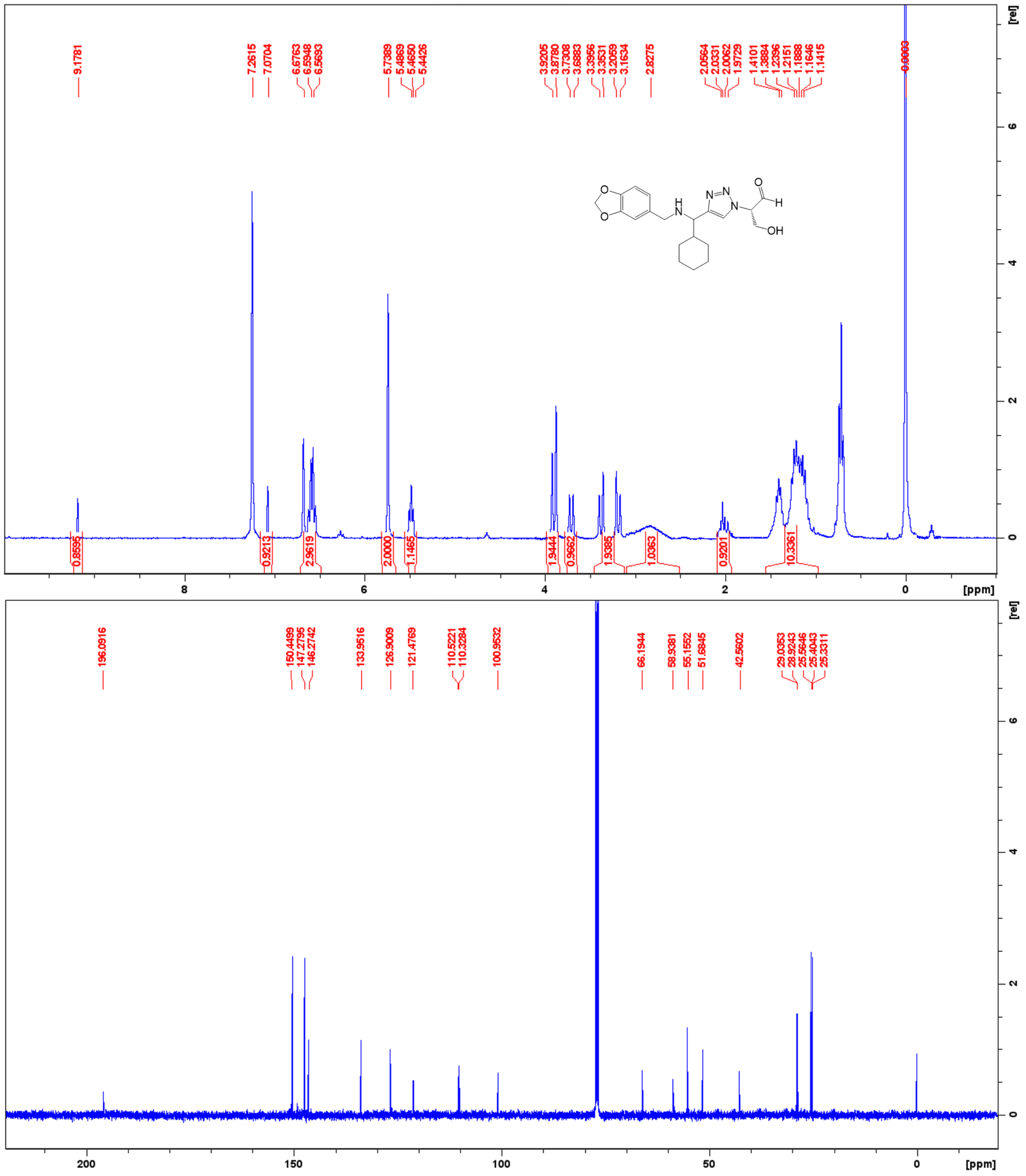

^{1}H NMR and ^{13}C NMR spectra of **Ald-10**

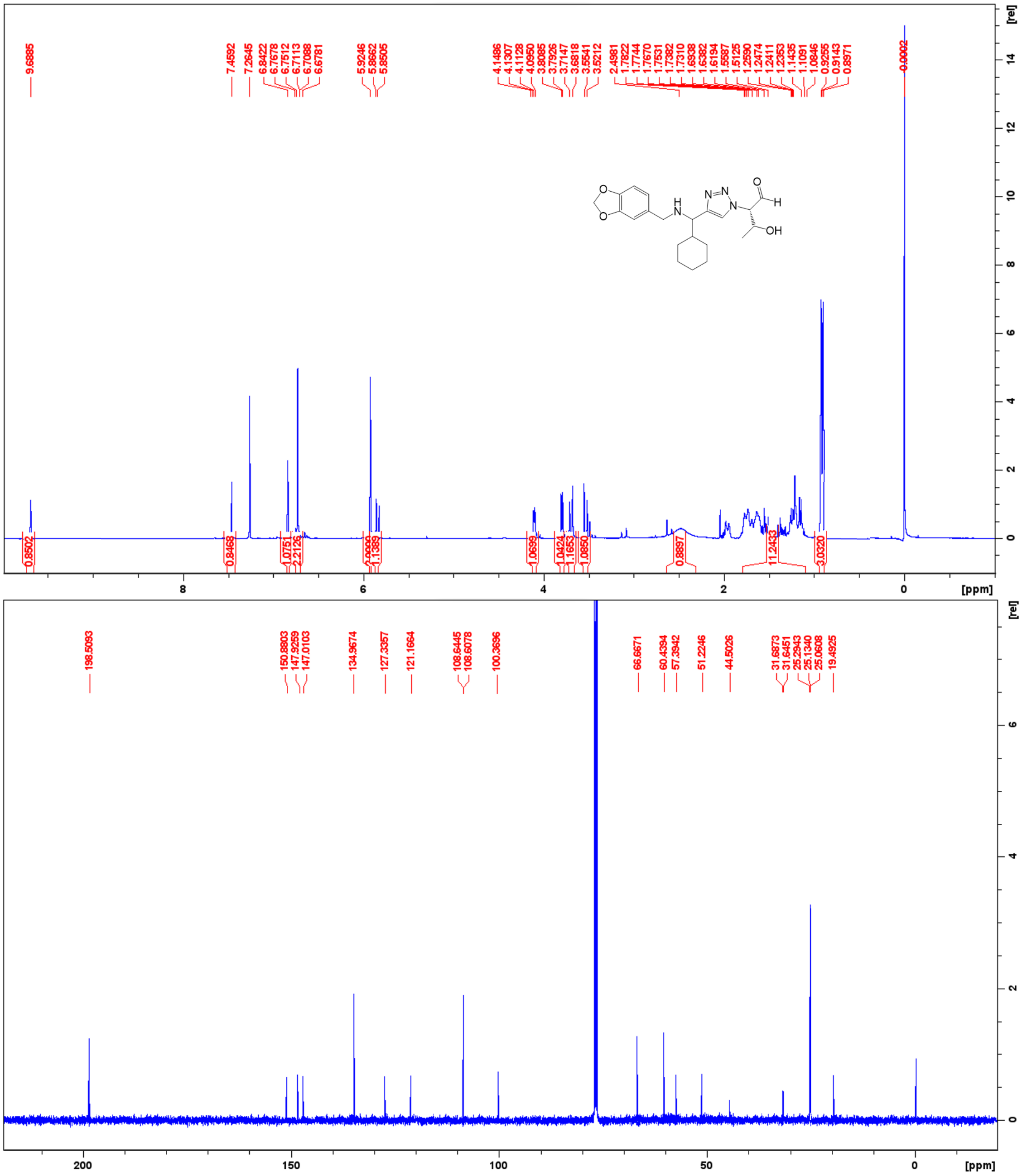

^{1}H NMR and ^{13}C NMR spectra of **Ald-11**

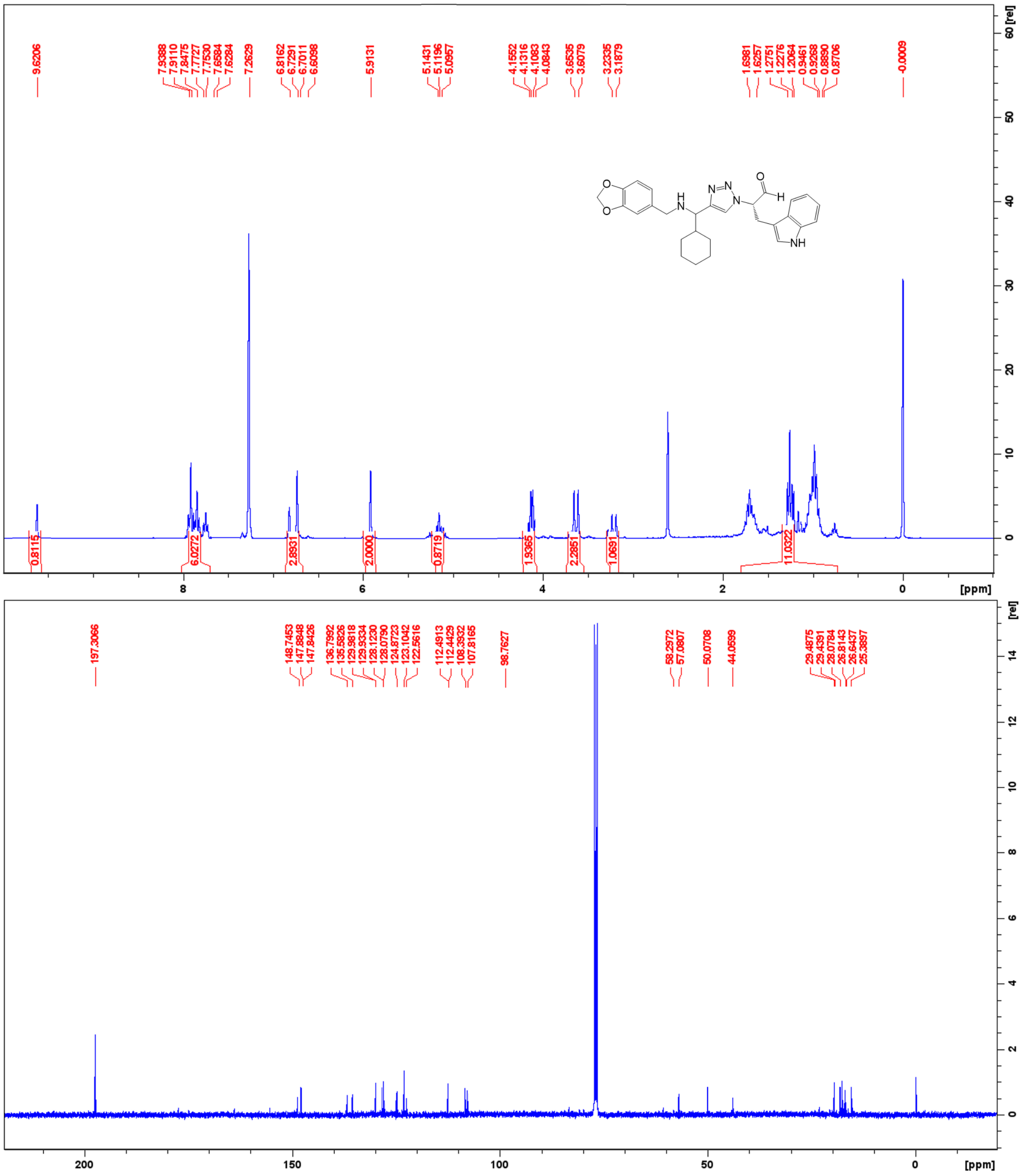

^{1}H NMR and ^{13}C NMR spectra of **Ald-12**

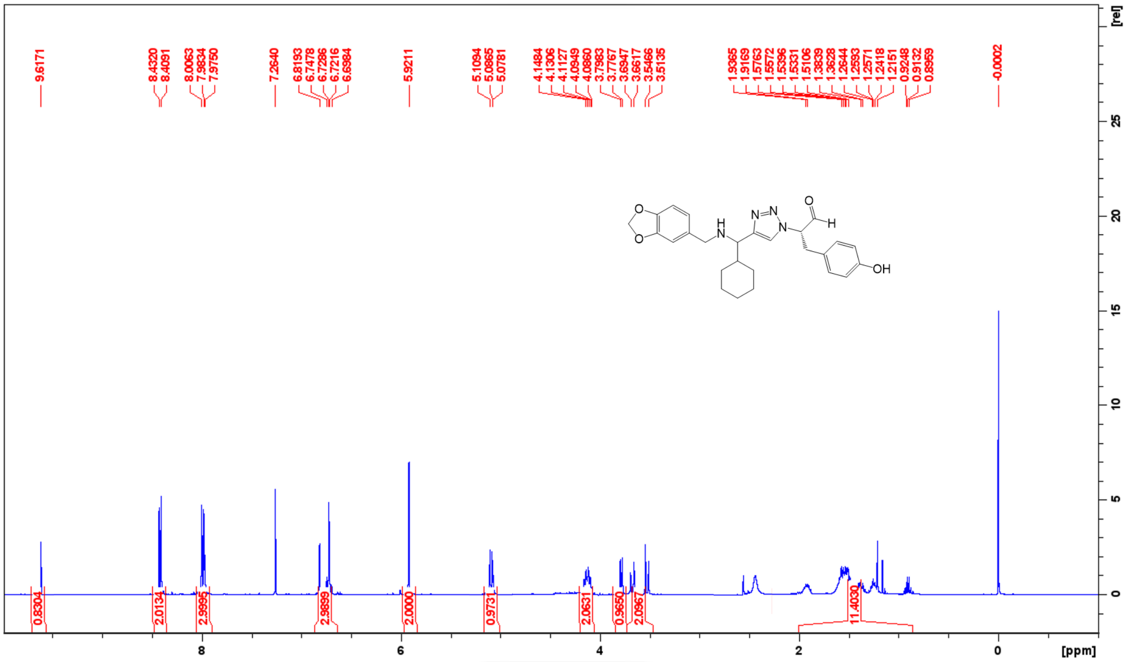

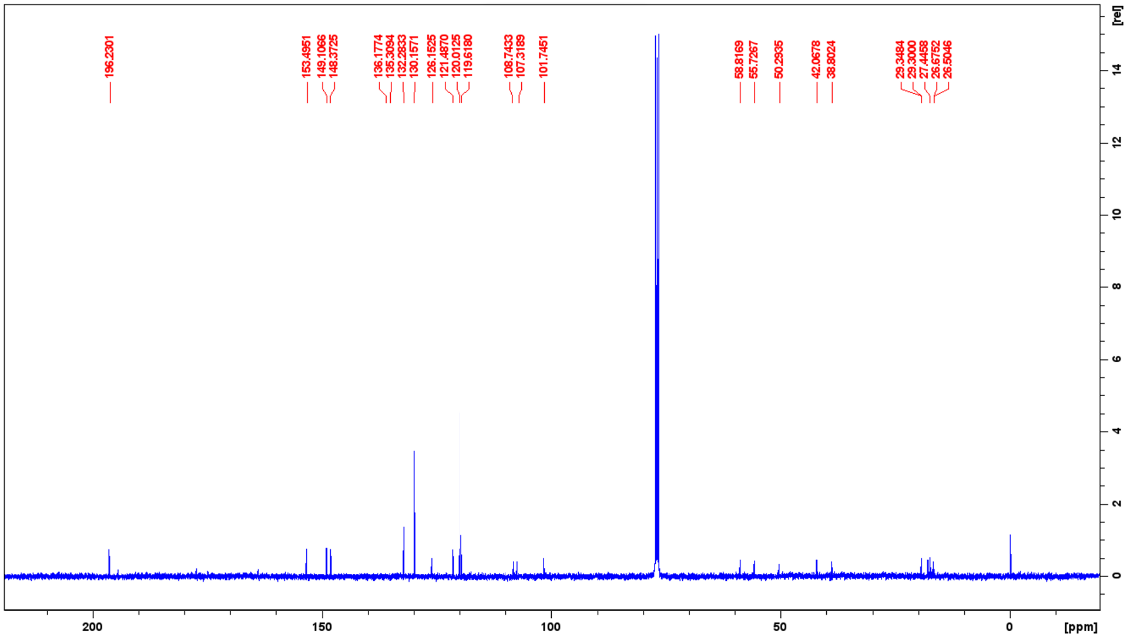

^{1}H NMR and ^{13}C NMR spectra of **Ald-13**

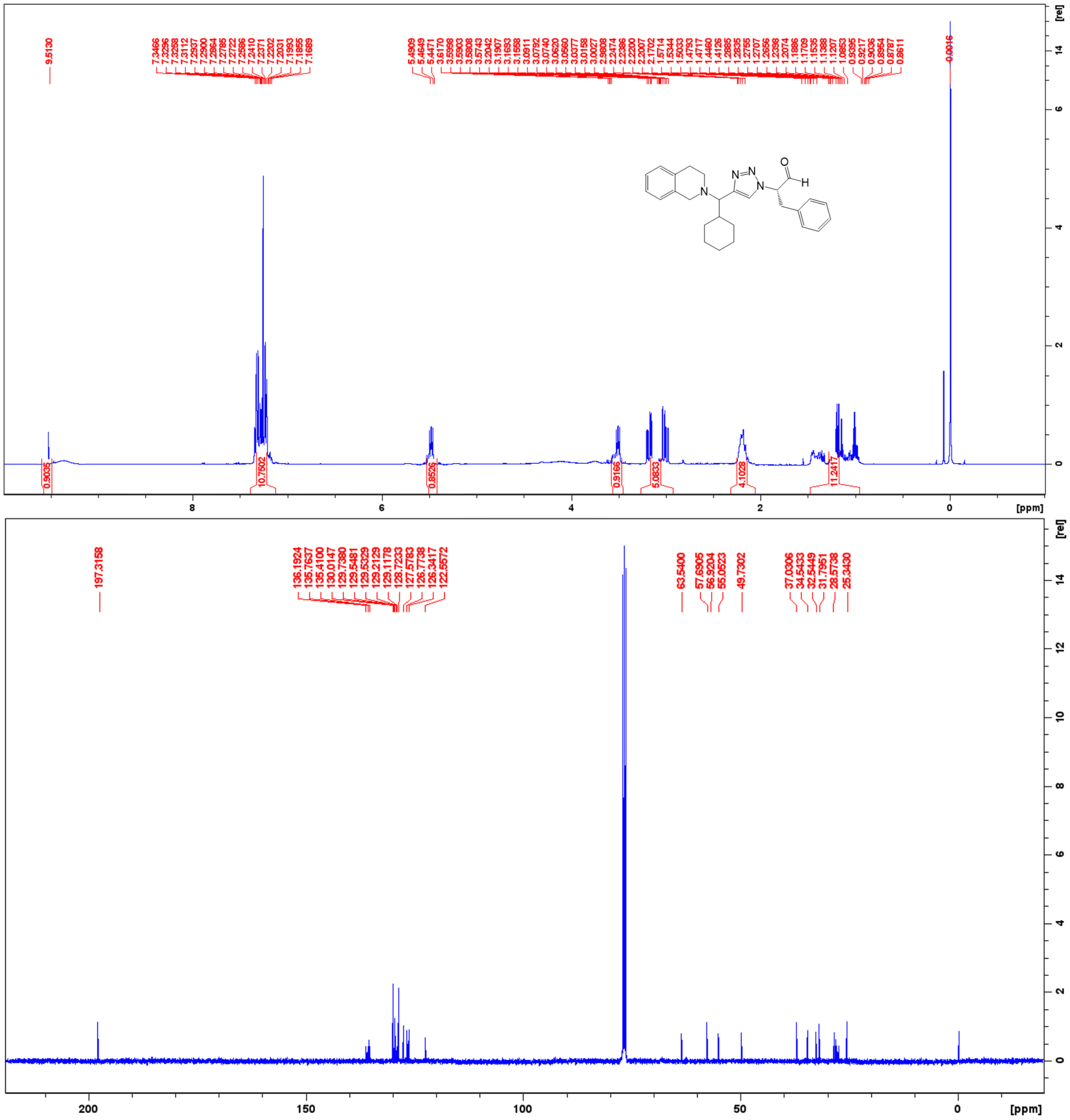

^{1}H NMR and ^{13}C NMR spectra of **Ald-16**

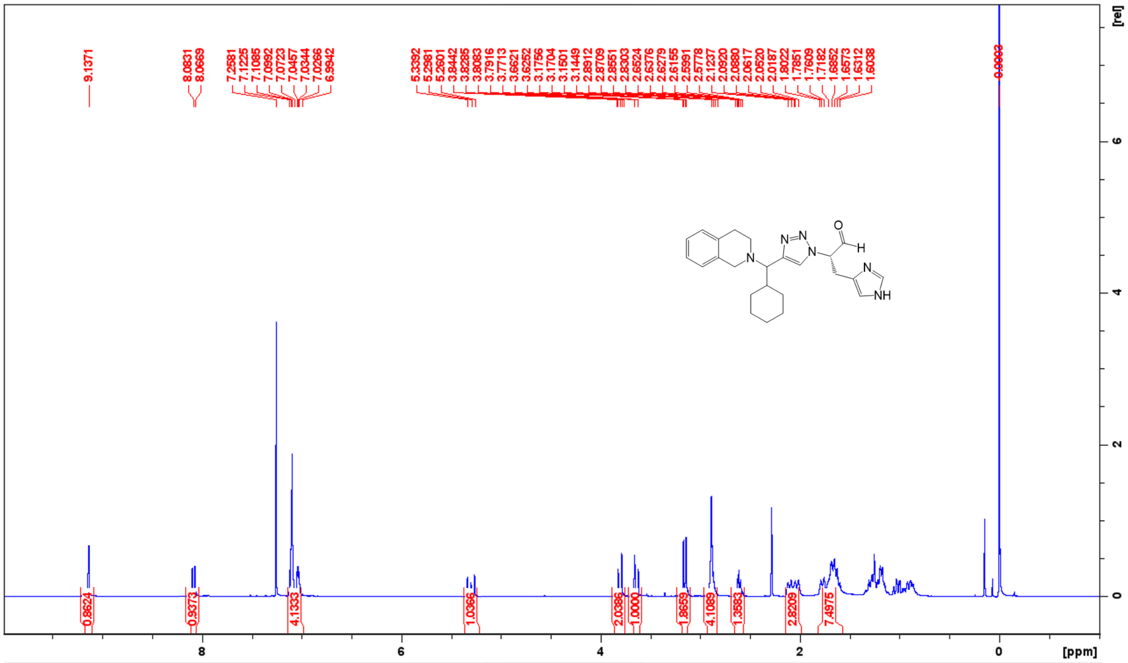

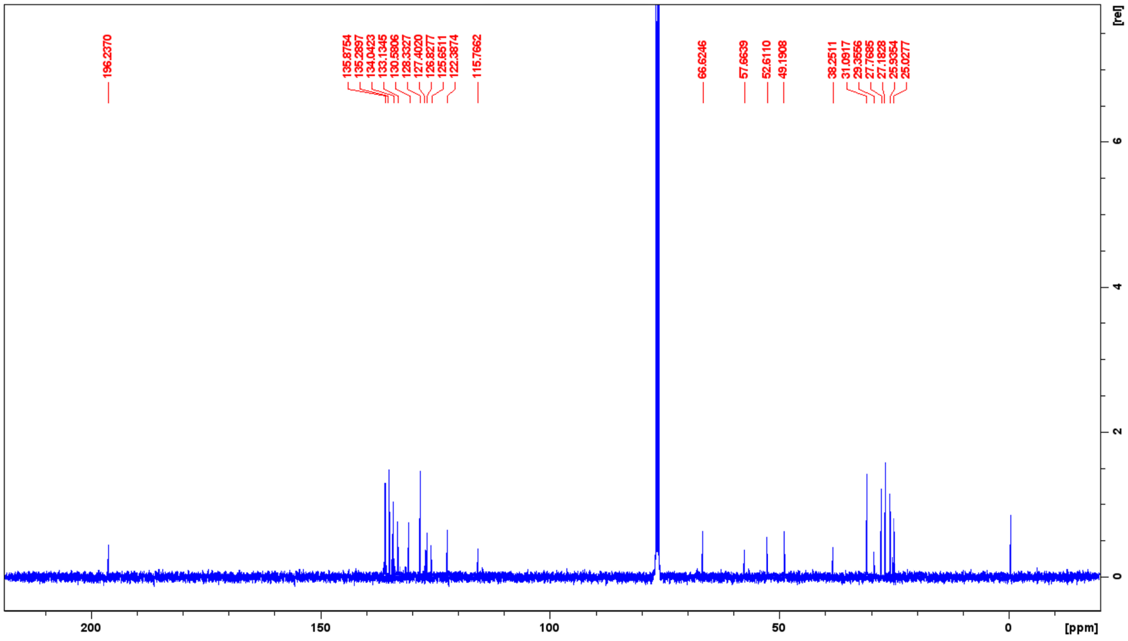

^{1}H NMR and ^{13}C NMR spectra of **Ald-17**

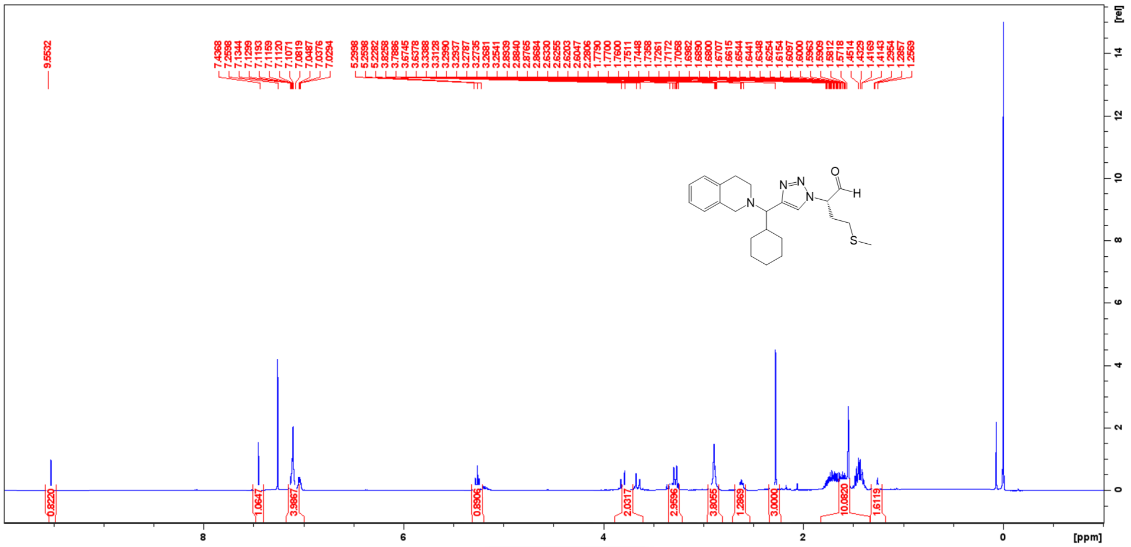

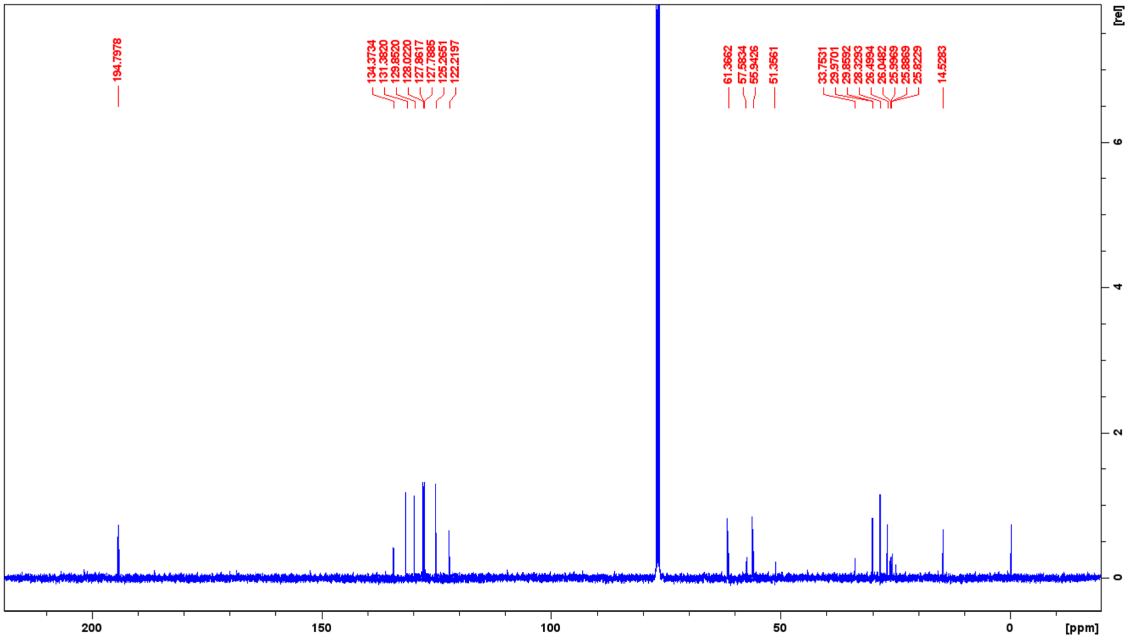

^{1}H NMR and ^{13}C NMR spectra of **Ald-18**

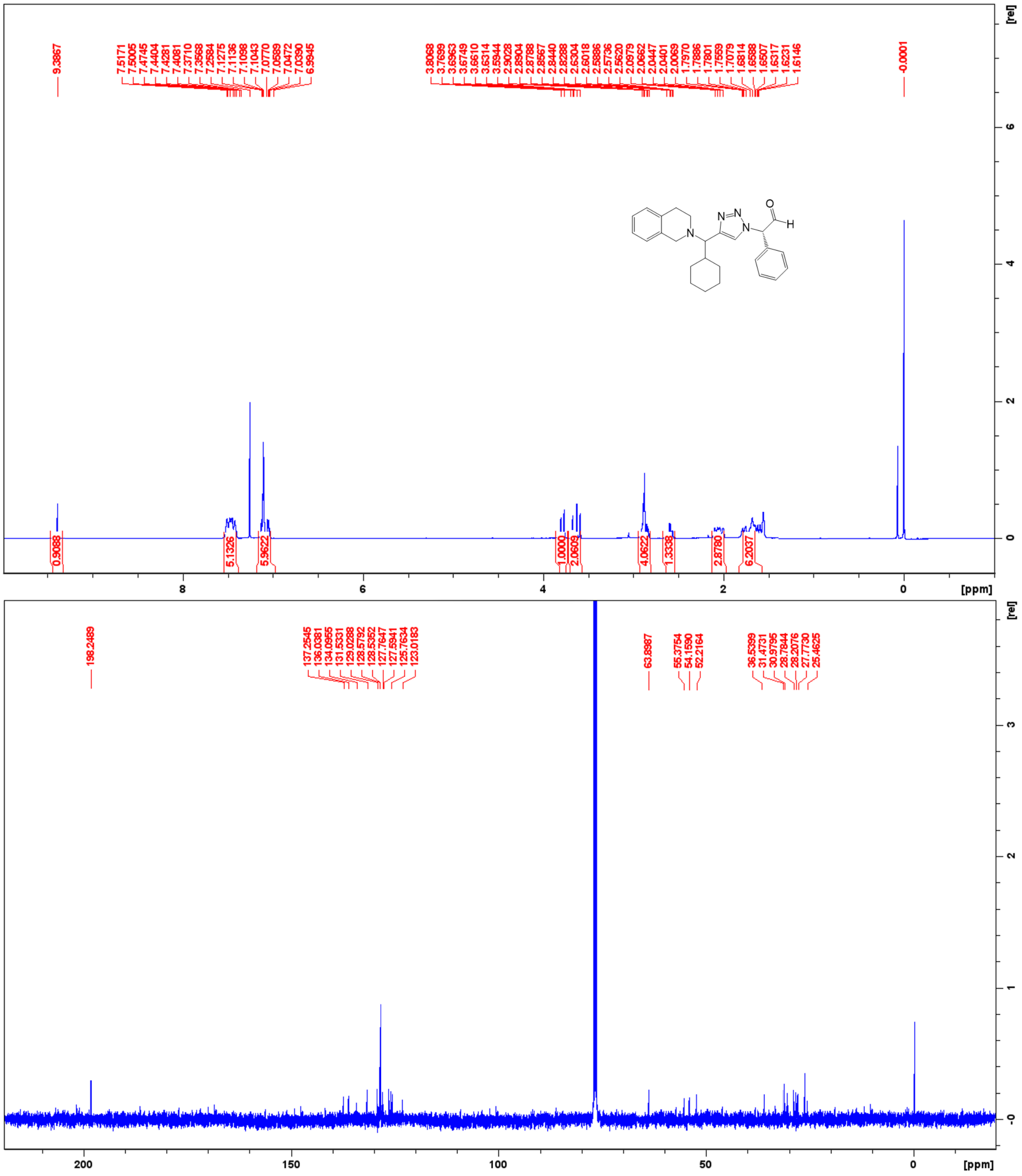

^{1}H NMR and ^{13}C NMR spectra of **Ald-19**

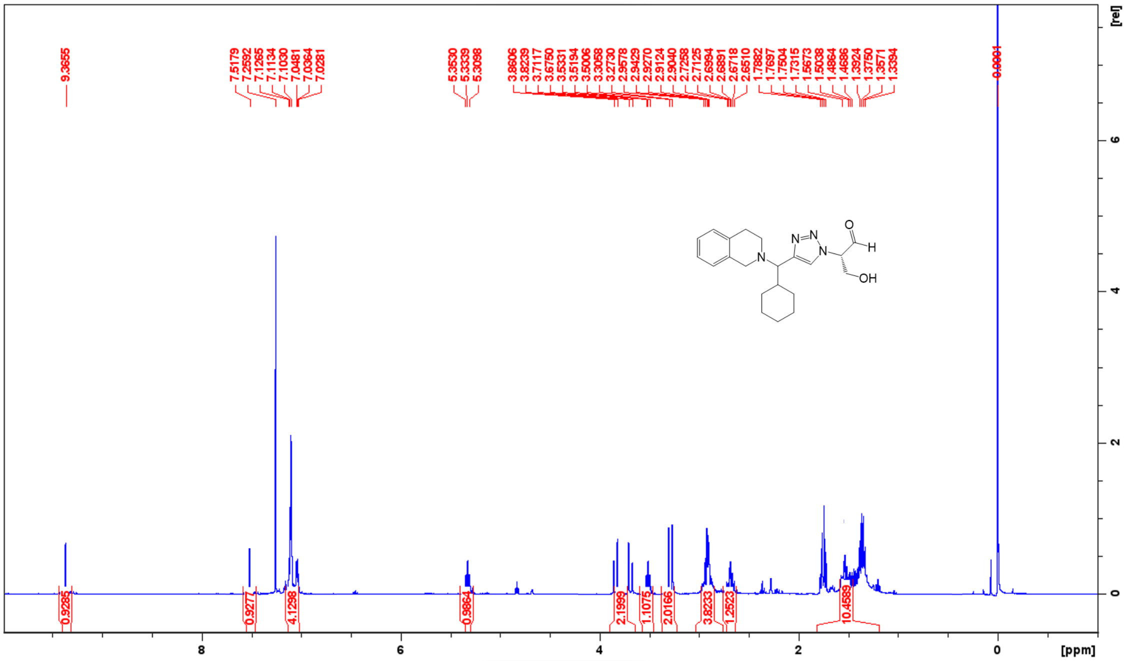

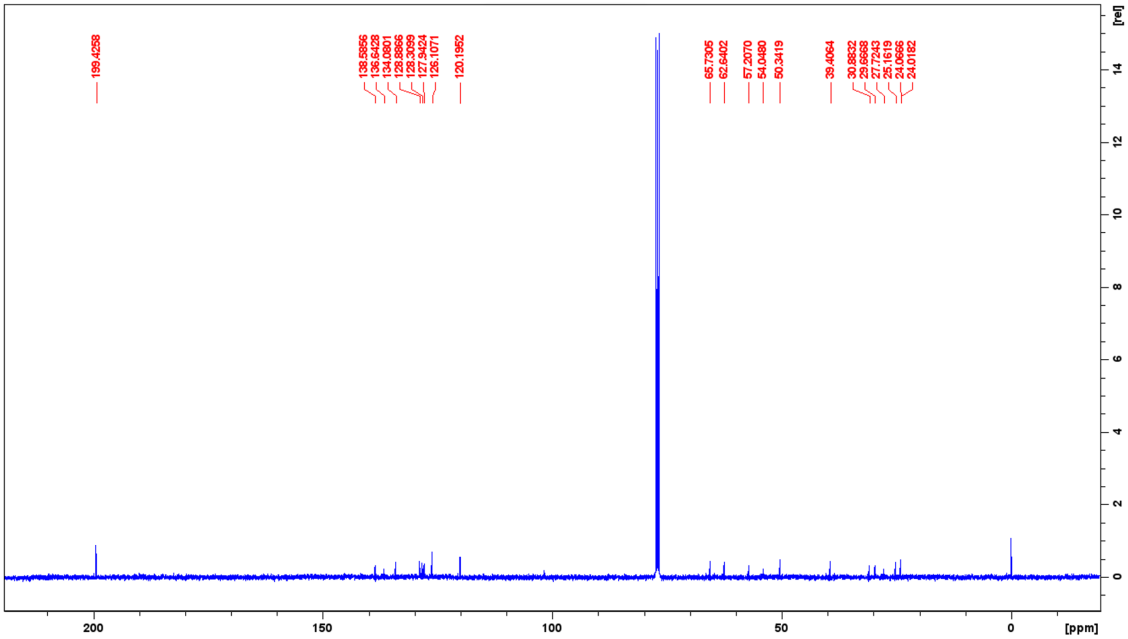

^1H NMR and ^{13}C NMR spectra of **Ald-20**

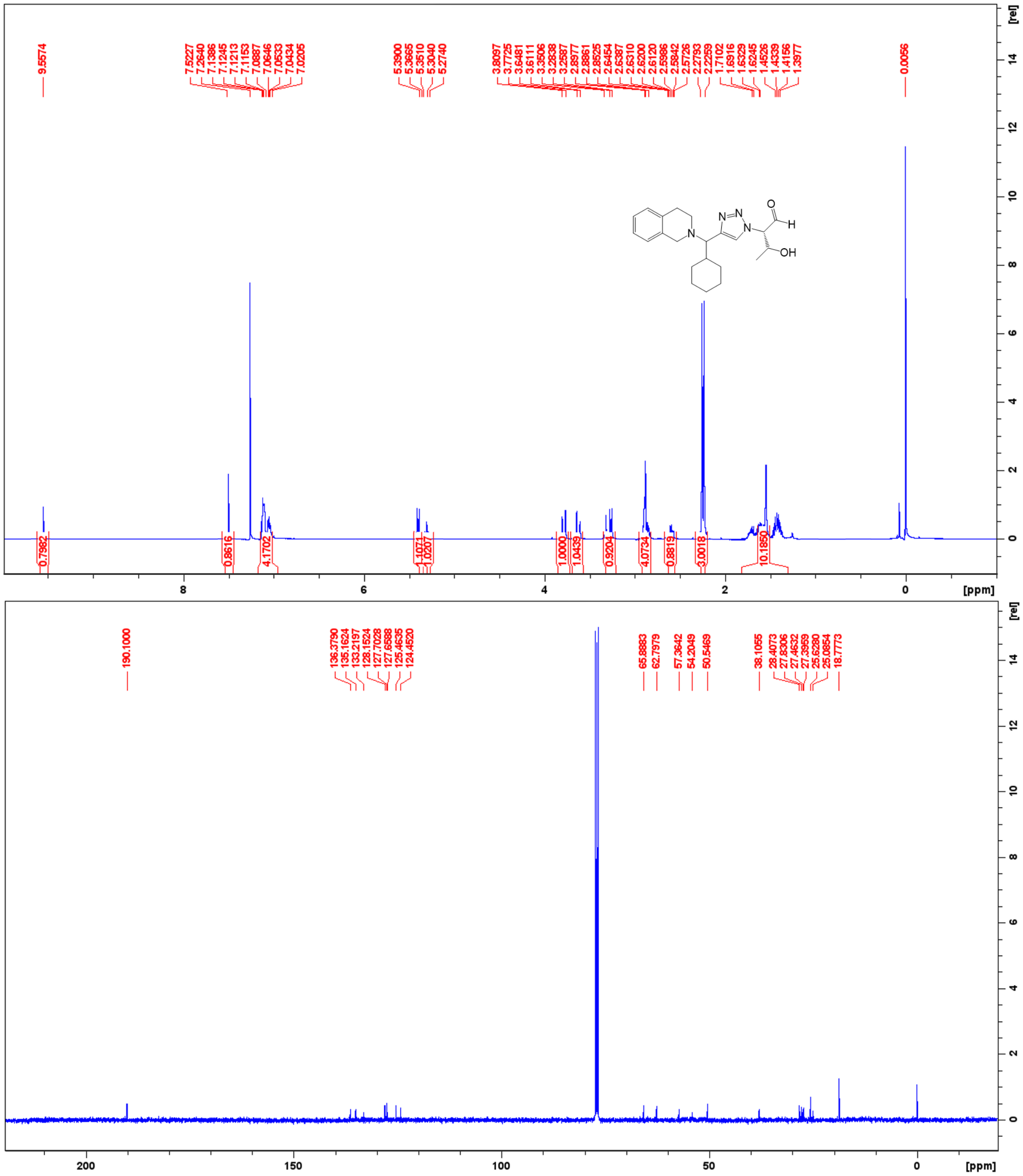

^{1}H NMR and ^{13}C NMR spectra of **Ald-21**

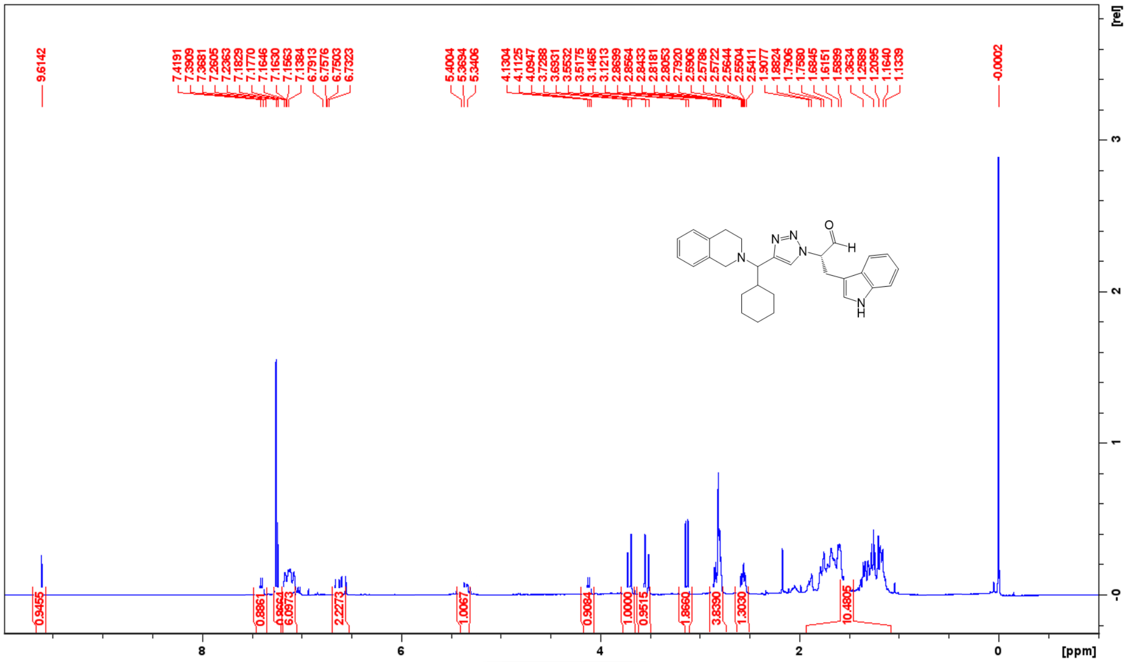

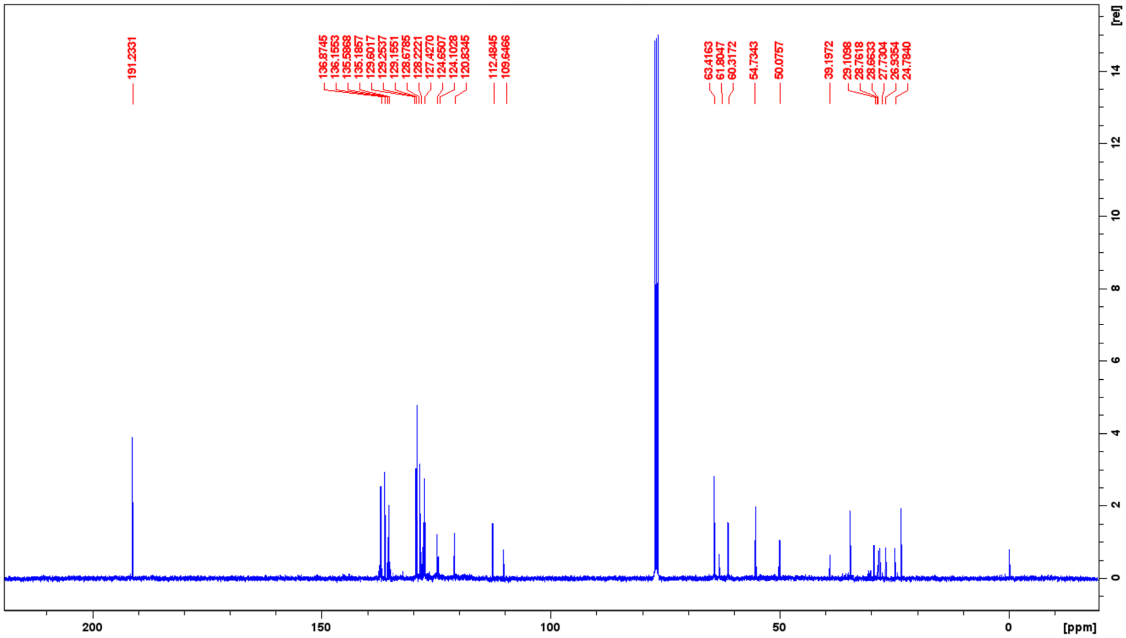

^{1}H NMR and ^{13}C NMR spectra of **Ald-22**

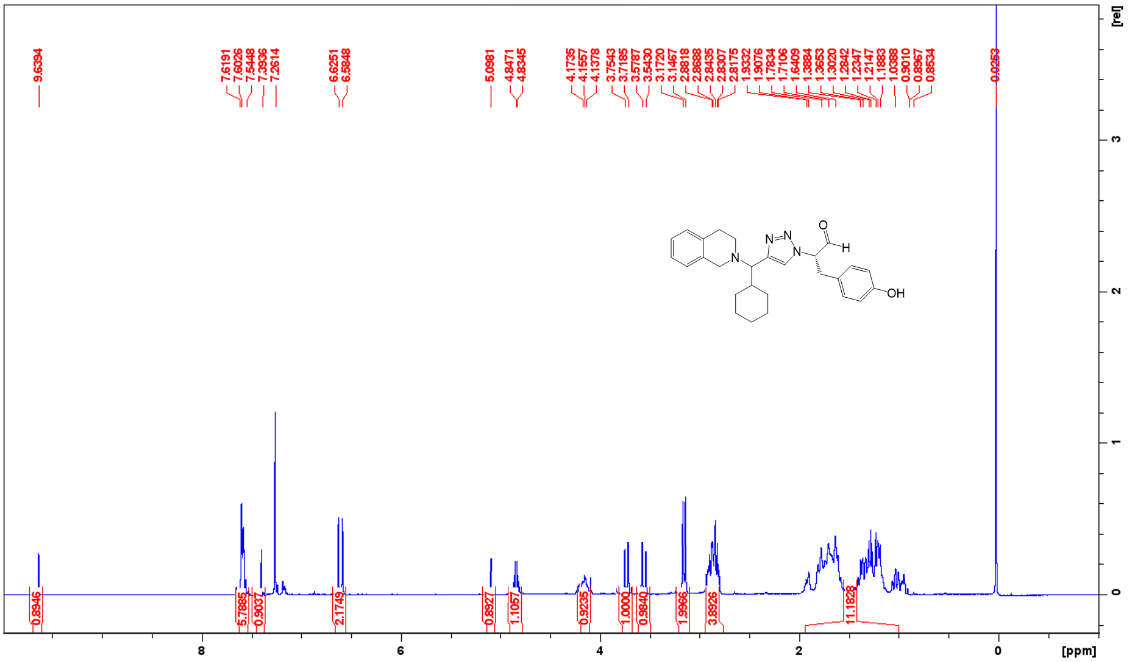

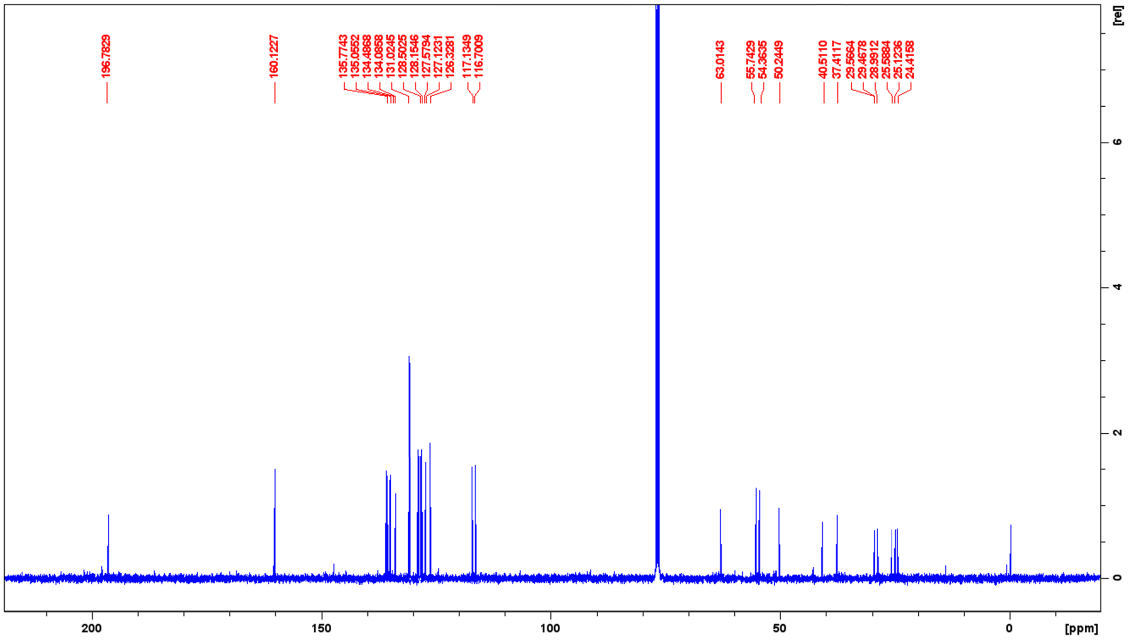

^{1}H NMR and ^{13}C NMR spectra of **Ald-23**

References

(1) Neitz, R.; Bryant, C.; Chen, S.; Gut, J.; Caselli, E.; Ponce, S.; Chowdhury, S.; Xu, H.; Arkin, M.; Ellman, J.; Renslo, A. Tetrafluorophenoxymethyl ketone cruzain inhibitors with improved pharmacokinetic properties as therapeutic leads for Chagas' disease. *Bioorganic & medicinal chemistry letters* **2015**, *25*, 4834–4837.

(2) Chen, Z.; Sun, T.; Qing, G. cAMP-modulated biomimetic ionic nanochannels based on a smart polymer. *Journal of Materials Chemistry B* **2019**, *7*, 3710–3715.

(3) Ermolat'ev, D.; Feng, H.; Song, G.; Van der Eycken, E. Copper (I)-Catalyzed Decarboxylative Coupling of Propiolic Acids with Secondary Amines and Aldehydes. *European Journal of Organic Chemistry* **2014**, *2014*, 5346–5350.
